# Deciphering deterioration mechanisms of complex diseases based on the construction of dynamic networks and systems analysis

**DOI:** 10.1038/srep09283

**Published:** 2015-03-19

**Authors:** Yuanyuan Li, Suoqin Jin, Lei Lei, Zishu Pan, Xiufen Zou

**Affiliations:** 1 School of Mathematics and Statistics, Wuhan University, Wuhan 430072, China; 2 State Key Laboratory of Virology, College of Life Sciences, Wuhan University, Wuhan 430072, China; 3 School of Science, Wuhan Institute of Technology, Wuhan 430074, China

## Abstract

The early diagnosis and investigation of the pathogenic mechanisms of complex diseases are the most challenging problems in the fields of biology and medicine. Network-based systems biology is an important technique for the study of complex diseases. The present study constructed dynamic protein-protein interaction (PPI) networks to identify dynamical network biomarkers (DNBs) and analyze the underlying mechanisms of complex diseases from a systems level. We developed a model-based framework for the construction of a series of time-sequenced networks by integrating high-throughput gene expression data into PPI data. By combining the dynamic networks and molecular modules, we identified significant DNBs for four complex diseases, including influenza caused by either H3N2 or H1N1, acute lung injury and type 2 diabetes mellitus, which can serve as warning signals for disease deterioration. Function and pathway analyses revealed that the identified DNBs were significantly enriched during key events in early disease development. Correlation and information flow analyses revealed that DNBs effectively discriminated between different disease processes and that dysfunctional regulation and disproportional information flow may contribute to the increased disease severity. This study provides a general paradigm for revealing the deterioration mechanisms of complex diseases and offers new insights into their early diagnoses.

Many complex diseases, including cancer, depression, inflammatory bowel disease, diabetes, obesity and heart disease, are caused by perturbations of complex intracellular and intercellular networks that link tissue and organ systems^1^,^2^. Recent studies have demonstrated that many complex diseases, such as asthma attacks^3^, prostate cancer^4^ and depression^5^, have tipping points, at which the diseases are irreversible. The detection of the pre-disease state of complex diseases is important for preventing qualitative deterioration through appropriate intervention actions. However, prevention is a very challenging task because pre-disease states are usually viewed as the limit of the normal state immediately before a critical transition occurs^6^,^7^,^8^.

Network-based approaches have recently emerged as powerful tools for the study of complex diseases^2^. One novel concept is the dynamical network biomarkers (DNBs) (i.e., a group of genes or proteins), which serve as a general early warning signal of a sudden deterioration before a critical transition occurs during disease initiation and progression. During this transition, the biological system is steered from a normal (or stable) state to a disease state. Previously, a composite index (*CI*) was theoretically derived to identify DNBs^9^. This index was subsequently applied to the quantification of edge biomarkers in corresponding edge-based networks^10^. Many complex diseases can use the *CI* or its derivational form to detect sudden deteriorations and discover underlying mechanisms during disease progression, such as lung injury disease^9^, diabetes mellitus^11^,^12^ and influenza^10^.

The occurrence and development of complex diseases change dynamically, and dynamic networks can more accurately simulate disease processes associated with gains, losses or modifications of gene functions through evolutionary time^13^,^14^. The existing methods for the identification of DNBs were based on dynamic networks, but these methods viewed networks as being fully connected without considering the actual network connections or merely downloaded biomolecular interaction networks from various databases. The DNBs that were identified using these rough networks usually contained hundreds of genes. For example, the identified DNBs included 220 genes for acute lung injury^9^, 143 genes for H3N2 influenza^15^ and 104 genes for breast cancer^15^. The DNBs are usually a dominant module^11^. Previous studies have shown that biologically relevant functional modules of the desired size contained 10–100 genes, whereas very large modules were highly redundant^16^. Therefore, it is unrealistic to use these DNBs for clinical diagnoses.

To the best of our knowledge, few studies have constructed dynamic PPI networks using time-course microarray data^17^,^18^,^19^,^20^,^21^,^22^,^23^. No study has reported the combination of prior knowledge regarding network topologies with optimization algorithms for identifying dynamic PPI networks. This methodology may limit the effectiveness of network-based strategies for the characterization and early diagnosis of complex diseases. The present study addresses these limitations by combining microarray data, novel computational methods and metrics-based interfering DNBs to construct and quantitatively analyze dynamic PPI networks. We propose a general paradigm for making an early diagnosis and unveiling the deterioration mechanisms of complex diseases (Figure 1). Furthermore, we apply this paradigm to four complex diseases, including influenza caused by the H3N2 virus^24^, influenza caused by the H1N1 virus^25^, lung injury induced by carbonyl chloride inhalation exposure^26^ and type 2 diabetes in rat adipose tissues^27^. These model-based computations highlight novel DNB modules and their critical role in disease deterioration. Our findings suggest a hypothesis for clinical diagnosis and a novel therapeutic strategy for complex diseases.

## Results

**Figure 1:**
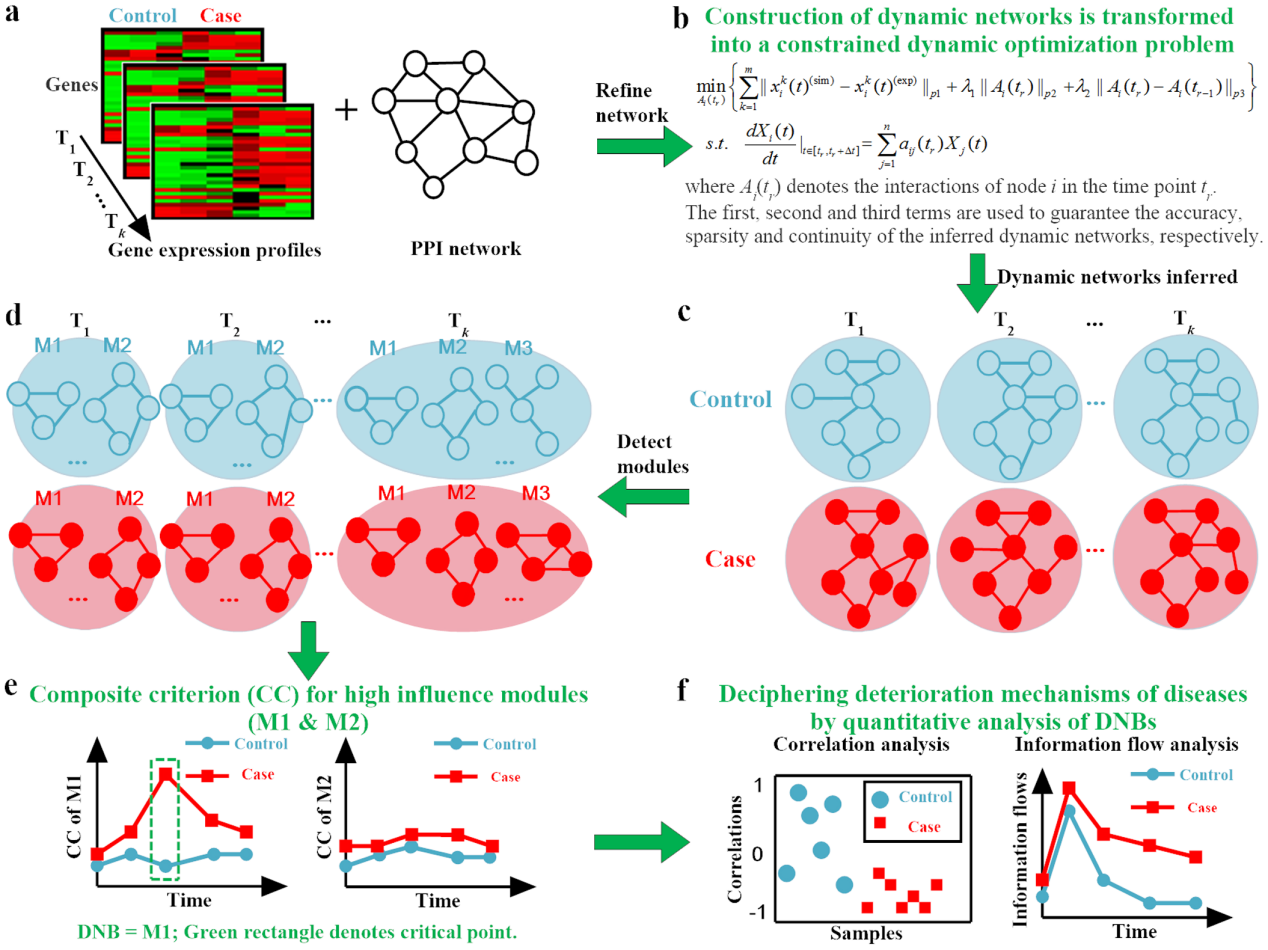
Overview of the proposed paradigm for making early diagnoses and unveiling the deterioration mechanisms of complex diseases. (a) Comparative time-series gene expression profile of binary conditions (Control vs. Case) was generated using the high-throughput technologies. Rows are genes and columns samples. Prior knowledge of PPI network was integrated with the high-throughput data to construct dynamic networks. (b) The further network inference using the ODE-based dynamic optimization method. The framework of network construction is depicted in Supplementary Figure S1. (c) Comparison of the inferred dynamic networks between the control and case conditions. (d) Modules were detected in the temporary network. (e) High-influence modules that appeared in each time point for both the control and case conditions were selected to identify DNBs using the composite criterion (CC). The framework of DNB identification is shown in Supplementary Figure S2. (f) Quantitative analyses of DNBs, including the correlation and information flow analysis, were employed to decipher the deterioration mechanisms of diseases.

We established a general paradigm to make an early diagnosis and reveal the deterioration mechanisms of complex diseases by constructing dynamic networks and performing system analyses. The proposed paradigm is depicted in Figure 1. We applied our proposed methodology to the high-throughput real microarray data of four diseases, namely live influenza infection (humans) caused by the H3N2 (GSE30550) and H1N1 (GSE52428) viruses, acute lung injury (mice) induced by carbonyl chloride inhalation exposure (GSE2565) and type 2 diabetes in rat adipose tissue (GSE13268).

### Construction of dynamic networks for control and case conditions

In the present study, we developed a model-based framework for the construction of dynamic regulatory networks using the integration of gene expression profiles with a prior knowledge of PPI networks (see Methods and Supplementary Figure S1). First, the initial PPI network was constructed for each dataset using PPI databases. Next, based on the initial PPI network and each dataset, the MI that measured the non-linear dependence between paired nodes was used to filter the highly noisy interactions, allowing us to obtain a series of time-sequenced refined networks. We then built ordinary differential equation (ODE) models for these time-sequenced networks and used optimization algorithms to construct cell-specific regulatory networks. We also further removed the redundant regulations in a series of time-sequenced rewired networks to identify the parameters in the models. Finally, we determined whether the interactions between two proteins were significant by setting a threshold value for the optimized parameters.

We applied the above framework to four datasets containing control (normal) and case (disease) samples. Therefore, we could construct 3 (diseases) × 2 (conditions) = 6 context-based dynamic networks for the control and case conditions of the four complex diseases. The dynamic networks in the case conditions are displayed in Supplementary Figures S10–S13. The basic information for the dynamic networks of the four diseases, including the number of nodes and edges in each time point, is listed in Supplementary Tables S4–S7.

To quantify the accuracy of the constructed dynamic networks, we defined two types of errors: average absolute error (AAE) and average relative error (ARE) (Supplementary text). Table 1 summarizes the average values of the AAEs and the AREs for the control and case dynamic networks. The lower AAEs and AREs indicate higher accuracy. The average values of AAEs for the four datasets were less than 0.4, and those of AREs were less than 0.04. These results showed the accuracy of the constructed dynamic networks.

Furthermore, leave-one-out cross-validation (LOOCV) (Supplementary text) was performed to assess the reliability of the constructed dynamic networks. The performances of our method with respect to Sensitivity (SN), Specificity (SP), False positive rate (FPR), Accuracy (ACC) and Matthews coefficient constant (MCC) for the four datasets are shown in Table 2. Table 2 shows that all ACCs were higher than 0.99. These results showed the reliability of the constructed dynamic networks.

### Identification of DNBs for complex diseases

We first used the ClusterONE algorithm^28^ based on the constructed dynamic networks described above to detect protein modules at each time point in the control and case networks (Supplementary Tables S8–S15). The conserved protein modules that appeared in both the control and case networks were obtained for the four datasets according to the defined similarity between two given modules (Supplementary Tables S16–S19). Moreover, we proposed a new concept, ''high influence modules'', to further quantify the importance of a module in a network (See Methods). We used the influence index of a module (*IIM*) to calculate the influences of these conserved modules for the four datasets. Comparisons of the influence index for all the conserved modules for the four datasets are depicted in Supplementary Figures S14 and S15. Table 3 lists the four high influence modules (HM1, HM2, HM3 and HM4) in the control and case networks for the four datasets.

**Figure 2:**
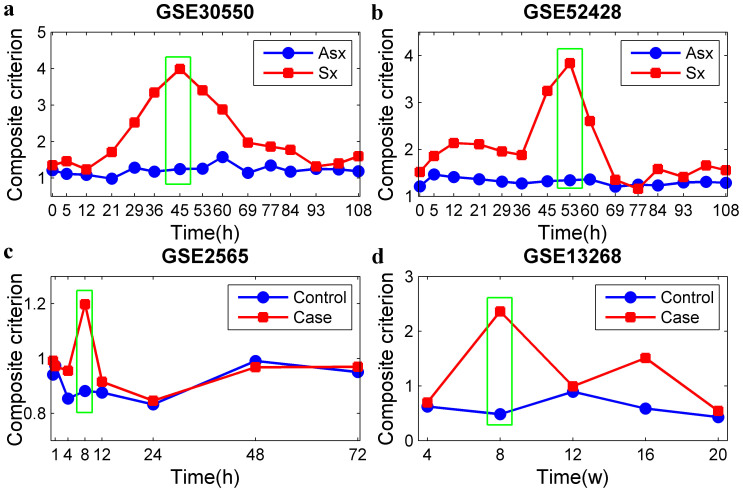
Comparisons of composite criterions (CCs) of HM1, HM2, HM3 and HM4 between the control and case networks for the four high-throughput experimental datasets. (a) HM1 for GSE30550 dataset; (b) HM2 for GSE52428 dataset; (c) HM3 for GSE2565 dataset; (d) HM4 for GSE13268 dataset. The green rectangle indicates the time of the pre-disease state. Blue line with circle markers and red line with square markers represent the composite criterion of the control (asymptomatic (Asx)) and case (symptomatic (Sx)) networks, respectively.

We then calculated the composite criterions (*CCs*) of all identified modules. The comparisons of *CCs* between the control and case networks for high influence modules (HM1, HM2, HM3 and HM4) (Table 3) for the four datasets are shown in Figure 2, and the *CCs* for other conserved modules (Supplementary Tables S16–S19) are shown in Supplementary Figure S16. These results indicated that the *CCs* of the high influence modules (HM1, HM2, HM3 and HM4) were obviously different from the other modules in the control and case networks.

Biological experiments for human influenza infection caused by the H3N2 virus (GSE30550) indicated that symptom onset began at an average of 49.3 hours after inoculation and patients who became ill experienced maximal symptoms at an average of 90.6 hours after inoculation^24^. Biological experiments for human influenza infection caused by the H1N1 virus (GSE52428) demonstrated that symptom onset began at an average of 61.3 hours after inoculation and patients who became ill experienced maximal symptoms at an average of 102.7 hours after inoculation^25^. Figure 2a and Figure 2b show that the *CCs* of the HM1 and HM2 modules exhibited almost no changes during the time points corresponding to the normal state, but the *CCs* of these two modules abruptly increased and then decreased at 45 and 53 hours post-inoculation, respectively. These results indicated that *CCs* reflect pre-disease states prior to critical deteriorations, which was consistent with the observed biological phenotypes of datasets GSE30550 and GSE52428. Therefore, the detected HM1 and HM2 modules may be useful as potential DNBs for human influenza infection.

Biological experiments (GSE2565)^26^ revealed that the most prominent physiological effects occurred within the first 8 hours after exposure, resulting in the increase of pulmonary edema and ultimately a decrease in survival rates. In mice with acute lung injury induced by carbonyl chloride inhalation exposure, a 50%–60% mortality was routinely observed after 12 hours, and a 60%–70% mortality was observed after 24 hours^26^. Figure 2c shows an obvious rise in *CCs* at 8 hours, indicating that the prediction based on the DNBs coincides with the actual disease development.

In rats at the age of 8 weeks, there was a critical transition period for adipose in the disease evolution of T2DM (GSE13268) (Figure 2d). This finding was consistent with the development of T2DM in GK rats according to the experimental data provided previously^27^. These results demonstrated that the high influence modules mentioned above (Table 3) were the DNBs, and their *CC*s can be early warning signals for an early diagnosis of the four complex diseases. Descriptions of the DNBs for these diseases are presented in Supplementary Tables S20–S23.

### Module-based DNBs provide superior performance in early diagnosis

We used DAVID^29^ to perform functional enrichment analysis of the identified DNBs for the four diseases to evaluate the relevance of DNBs during the early stages of disease progression. Gene Ontology functional annotation of the DNB genes showed that these genes were significantly enriched in diseases-related biological processes (Supplementary Tables S24–S27, P-values < 0.001), indicating that DNB genes significantly overlapped with disease-associated genes.

**Figure 3:**
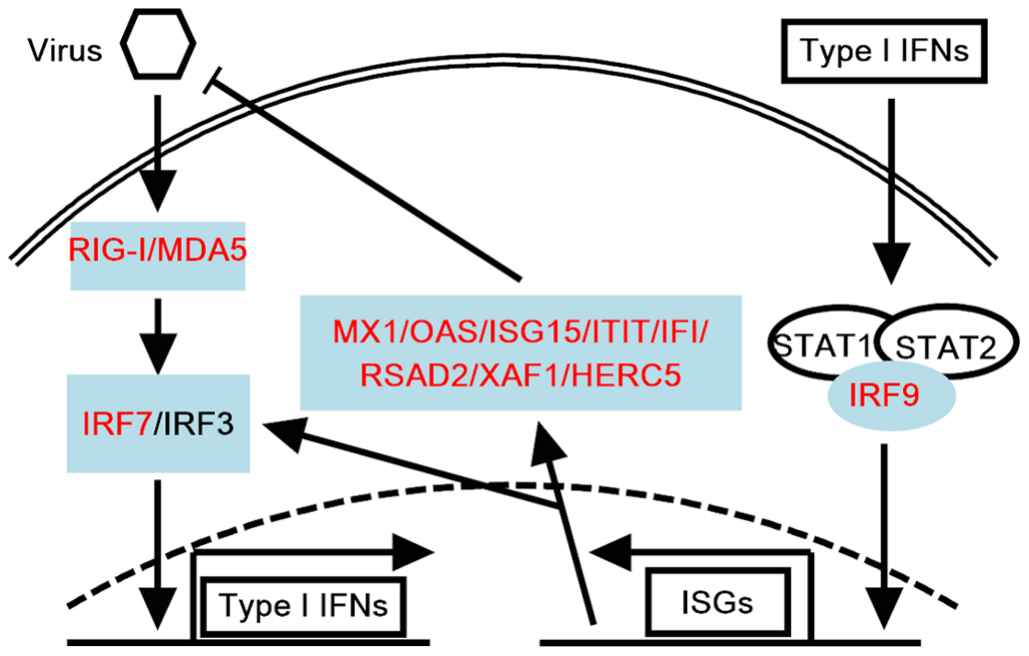
Schematic diagram of virus-triggered innate immunity pathway. DNBs are highlighted in red font and soft blue background. Virus activates RIG-I- or MDA5-mediated activation of NF-κB (not shown) and IRF3 or IRF7, which leads to transcription of the type I interferon (IFN) gene. Type I IFNs induce the expression of ISGF3 complex (consisting of STAT1, STAT2, and IRF9)-mediated IFN-stimulated genes (ISGs), which generates a large number of ISGs and antiviral proteins that can inhibit the viral replication.

We analyzed the functional enrichment of the high influence modules on a pathway level, as shown in Table 3 for the case (symptomatic) networks. The pathway enrichment analysis for the influenza virus infections showed that seven genes, including DDX58, MX1, OAS1, OAS2, OAS3, IFIH1 and RASD2, were observed in the Influenza A pathway (Supplementary Figure S17). We further conducted a comprehensive analysis of the literature for the DNBs. Notably, all of the DNBs participated in the innate immune response (Figure 3). RIG-I (retinoic acid-inducible gene I; encoded by DDX58) and MDA5 (melanoma differentiation-associated gene 5; encoded by IFIH1) were the dominant cellular pathogen-recognition receptors (PRRs). These proteins recognize viral nucleic acids and trigger an innate immunity response that includes the activation of transcription factors, such as IRF3, IRF7 and NF-κB, which induce the production of type I IFNs and IFN-stimulated genes (ISGs), such as OAS, ISG15, MX1 and IFITs^30^,^31^,^32^,^33^,^34^. This cascade normally results in an innate antiviral response that controls infection, but the excessive production of these proteins elicits an aberrant or disproportional response that results in immunopathology^32^,^35^. Supplementary Figures S18 and S19 reveal that the DNBs exhibited significantly higher expression levels in symptomatic H3N2 and H1N1 infections than in asymptomatic infections with these strains (P-values < 0.0001). The disproportional induction of DNBs may contribute to the severity of clinical symptoms.

Six genes in the Glycolysis or Gluconeogenesis pathway (Hk2, Aldoa, Ldha, Pgk1, Pkm2 and Tpi1) were observed in acute lung injury (Supplementary Figure S20). Eleven genes in the Glutathione metabolism pathway (Gpx7, Gss, Gsta3, Gstm2, Gstm5, Gstm7, Gsto1, Gstp1, Gstt1, Gstt2 and Mgst2) were observed in type 2 diabetes mellitus (Supplementary Figure S21). Diabetic subjects had lower concentrations of these genes compared to the control subjects at most time points (Supplementary Figure S22). This result is consistent with a previous study in which diminished glutathione synthesis was a hallmark of uncontrolled diabetes in patients^36^. Altogether, these related biological experiments confirmed that the identified DNBs were significantly enriched in key events during early disease development, which may improve early disease diagnosis.

There are also several methods for identifying DNBs^9^,^10^. For each disease, we detected one critical point before clinical symptoms appeared. This critical point was the same as previous studies for acute lung injury and T2DM. Two critical points for influenza infection have been found previously, but the one critical point identified by our method was nearly identical to the first critical point. Most of the genes that were identified by our method were also detected by the other two methods (common genes are noted in bold in Table 3), which indicates the reliability of our method. Our method detected a smaller number of DNBs, which is different than the hundreds of genes in the DNBs identified using other methods (Table 4). These results showed the power of our method because it simultaneously detected the critical points while also identifying DNBs with relatively small sizes. Generally speaking, the DNBs were a functional module, and biologically relevant functions were best described by modules ranging from 10–100 genes in size. Moreover, the size of a module influenced the probability of the module being a false positive^16^. Therefore, it is unrealistic for the DNBs that were identified using other two methods to be used for clinical diagnosis. In contrast, the DNBs discovered using our method are of great importance for clinical applications in realistic cases. In addition, the DNB genes found in previous studies were scattered in many different pathways^9^,^10^, and the genes were not connected into a molecular module. However, the DNB genes found by our method were actually densely connected and enriched in key pathways during disease progression. For example, the identified DNBs for influenza are critical to the innate immune response pathway. These results demonstrated that the module-based DNBs derived from the constructed dynamic networks provided superior performance in early diagnoses, and these genes and proteins can be used for clinical applications.

### DNBs are negatively correlated with the S1P1R protein in case networks

**Figure 4:**
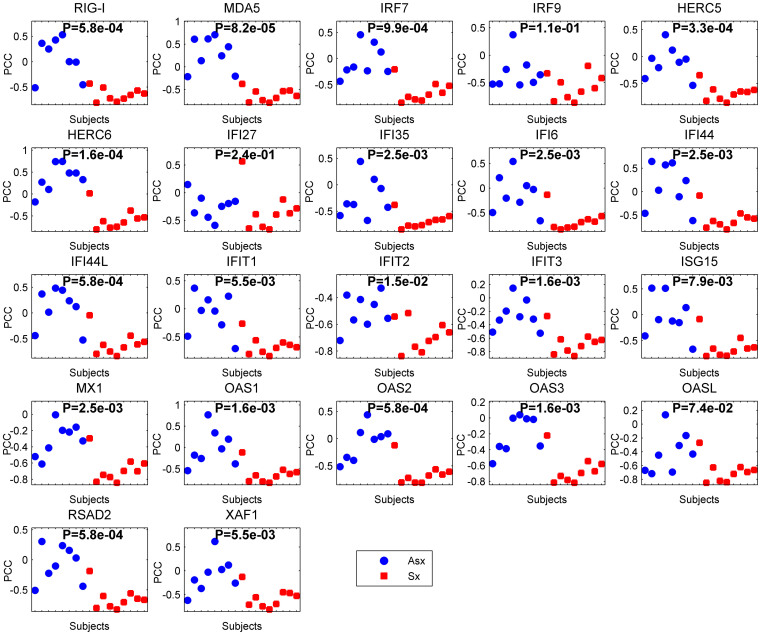
Comparison of the correlation distributions for the H3N2 strain. Blue circles and red squares indicate Pearson correlation coefficients (PCCs) between the S1PR1 expression values and DNBs in the asymptomatic infection (Asx) and symptomatic infection (Sx), respectively. X-axis represents the Asx or Sx subjects, namely control or case groups. Each data point corresponds to a correlation in one subject. P-values are from a two-tailed Wilcoxon rank-sum test.

The innate immune response is important for the regulation of viral infections. Therefore, there is considerable interest in investigating the underlying regulatory mechanisms of these DNBs. Earlier studies documented that sphingosine-1-phosphate receptor 1 (S1PR1) signaling suppresses detrimental innate immune responses and global cytokine-chemokine amplification, which plays an essential role in the clinical outcome and pathogenesis of influenza virus infection^37^,^38^. The Pearson correlation coefficients (PCCs) of expression levels of S1PR1 and key proteins of the innate immune response were computed to further assess the correlation between S1PR1 signaling and the innate immune response. Figure 4 shows that DNB gene expressions were inversely correlated with S1PR1 in most symptomatic subjects infected by the H3N2 strain. However, this correlation was not observed in asymptomatic subjects. Furthermore, significant differences were observed in the correlation distributions between the two different clinical outcomes (Figure 4, P-values < 0.05). The results of the H1N1 strain confirmed our findings (Supplementary Figure S23). These results suggested that there may be negative regulatory interactions between these DNBs and S1PR1 in symptomatic infections and different regulatory mechanisms in these two different clinical outcomes.

The relationships between S1PR1 and 14 other key proteins (IRF3, NFκB, IFN-α, IFN-β, TNF-α, IL6, TLR3, MyD88, IKKα, IKKβ, IKKε, TBK1, MAPK and IFN-γ) were not obvious in both clinical outcomes. Notably, the difference between the correlation distributions of the two clinical outcomes was not significant for the H3N2 and H1N1 strains (Supplementary Figures S24 and S25, P-values > 0.05). The detailed correlations between S1PR1 and these key proteins are presented in Supplementary Tables S28–S31. Altogether, the correlations between S1PR1 and these DNBs can discriminate symptomatic infections from asymptomatic infections, but other key proteins in the virus-induced innate immune response were ineffective. In addition, dysfunctional regulations between these DNBs and S1PR1 may underlie the increased disease severity.

### DNBs exhibit different information flow between normal and disease networks

**Figure 5:**
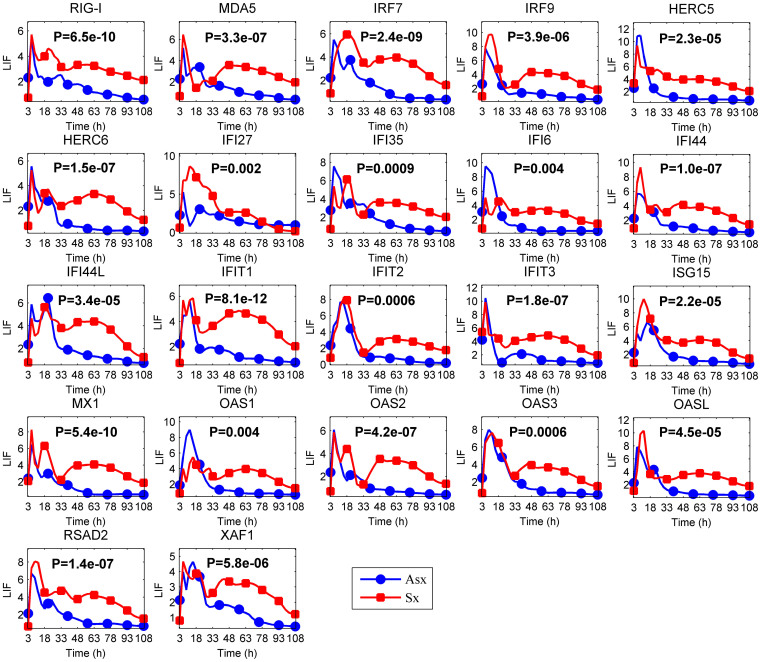
Comparison of local information flow (LIF) transmission from virus to DNBs in the symptomatic subjects (Sx) and asymptomatic subjects (Asx) infected by the H3N2 influenza strain. Blue and red lines denote local information flow in Asx and Sx, respectively. Two-sample t-tests showed that local information flows in the symptomatic subjects exhibited significantly higher values than in the asymptomatic subjects (P-values < 0.005).

**Figure 6:**
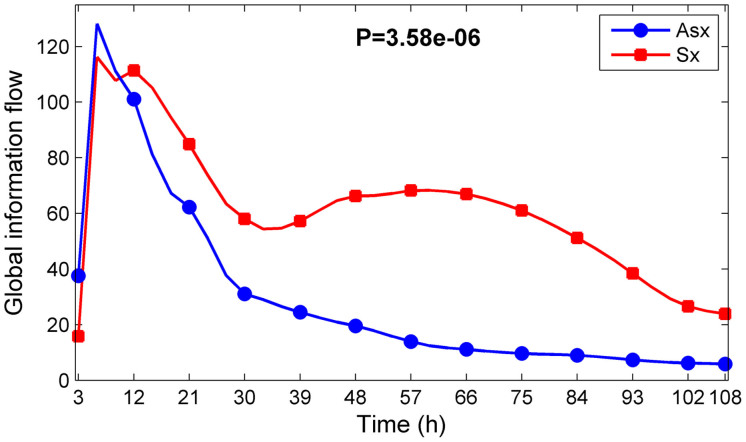
Comparison of global information flow transmission from the virus to DNBs in the symptomatic subjects (Sx) and asymptomatic subjects (Asx) infected by the H3N2 influenza strain. P-value is from a two-sample t-test. Blue and red lines denote the global information flows in Asx and Sx, respectively.

A better understanding of the transmission of information flows and how they affect cellular responses may provide new strategies to alter the outcome of complex diseases. Previous studies revealed that cells encode and decode cellular information to control the temporal behavior of their signaling molecules^39^,^40^,^41^. Therefore, we investigated whether the information flow transmission from the influenza virus to DNBs correlated with disease severity. We proposed a definition of information flow to test this hypothesis (see Methods). The experimental data had only fifteen time points. Cubic spline interpolation (using the Matlab toolbox) was used to obtain the interpolated time points at each half hour between 0 h and 108 h, which increased the accuracy of the information flow calculations. We calculated the information flow every three hours using the first few interpolated data before the current time point. Calculations and comparisons demonstrated that local information flows in the symptomatic subjects (disease network) exhibited significantly higher values than in the asymptomatic subjects (normal network) (Figure 5, P-values < 0.005). The global information flows of the disease networks were also significantly higher than normal networks (Figure 6, P-value = 3.58e − 06). These results indicated that the information flows of DNBs provided good discrimination between normal and disease networks, which generated different patterns of genetic expression.

The previous section showed that DNBs were enriched in virus-induced innate immune pathways. Therefore, we chose 14 vital proteins based on the literature^31^,^32^,^35^,^42^,^43^ and calculated information flow transmission from the virus to these proteins to investigate whether information flows of other important proteins of the innate immune pathways are also discriminated in the disease networks. Supplementary Figures S26 and S27 show that no significant differences were observed in local and global information flows between normal and disease networks. Taken together, these data demonstrated that the DNBs performed better than other proteins in characterizing the disease networks. The disproportional information flows of DNBs and the resulting immunopathology may also underlie the increased severity of symptoms in people during influenza virus infection.

## Discussion

This study developed a new method to construct dynamic networks based on the combination of high-throughput gene expression data, a prior knowledge of network topology and ODEs-based optimization. We also presented a novel computational framework for the detection of a critical stage and key DNBs during disease occurrence and progression from the constructed dynamical networks. The successful application of the framework in four real datasets demonstrated the effectiveness of our method in the identification of early warning signals of complex diseases. The application of our framework also provided a powerful way to capture deterioration mechanisms during disease development from information flows and statistical analyses.

Our study provides three main contributions. First, we proposed an efficient model-based framework to construct time-evolving networks, which is of practical relevance and importance to network analyses in diverse contexts ranging from biology to the social sciences. Dynamic network analysis provides a valuable model for biological functioning that reveals more disease-related information than static networks^13^,^44^,^45^. Second, the module-based method for detecting DNBs is a powerful tool for dynamically modeling the early development of complex diseases. Notably, the sizes of the identified DNBs using our method were of the desired module sizes and avoided redundancy, which can best describe the biologically relevant functions. Smaller module sizes are of great importance for clinical application in realistic cases because they provide a chemically tractable approach for effectively controlling control strategies of complex diseases. Third, we presented a new definition of global information flow and found that disease networks possessed more information, especially DNB information flows, that can provide good discrimination between normal and disease networks. These results further confirmed that the identified DNBs can be considered to be warning signals of diseases from the viewpoint of information transmission. This provides an interesting approach for revealing the deterioration mechanisms of diseases.

We applied our methodology to real data from four diseases, and the results provided new insights into deterioration mechanisms and early diagnosis of these diseases. Notably, we discovered that all of the DNBs for the two groups of influenza data were located in the virus-induced innate immunity signaling pathway. Furthermore, correlation analysis of S1PR1 and these DNBs indicated that dysfunctional regulations between these factors may lead to different clinical outcomes. Experimental and clinical validation of these predictions and hypotheses are required to further estimate their potential value, but our findings provide a significant foundation for further exploration of the molecular mechanisms of infectious diseases and the development of control strategies.

In summary, we established a paradigm for revealing the deterioration mechanisms of complex diseases by constructing dynamic networks and systems analysis and also provided new insights into the early diagnosis of complex diseases.

## Methods

### Data collection

Four gene expression profiling datasets were downloaded from the NCBI GEO database (GSE30550, GSE52428, GSE2565 and GSE13268)^46^. Probe sets from these datasets lacking the corresponding gene symbols were ignored in our analysis. The expression values of the probe sets that mapped to the same gene were averaged. The diseases from the first two datasets were two influenza strains, H3N2 and H1N1, whereas the other two datasets were for acute lung injury and type 2 diabetes mellitus.

The biological data GSE30550^24^ contained 17 healthy subjects who received intranasal inoculations of influenza H3N2/Wisconsin. Nine of these 17 subjects developed severe infection symptoms, and the other 8 subjects remained in good health. Gene expression profiles were measured in whole peripheral blood drawn from all subjects approximately every 8 hours post-inoculation (hpi) through 108 hpi. In total, 268 gene expression profiles were obtained for all subjects at 16 time points, including baseline (−24 hpi). The gene expression profiles of subject 8 at 21 hpi, subject 13 at baseline and 36 hpi, and subject 17 at 36 hpi were missing.

The biological dataset GSE52428^25^ contained 24 healthy subjects who received intranasal inoculations of influenza H1N1/Brisbane. 12 of these 24 subjects developed severe infection symptoms and 11 subjects remained in good health. One subject was excluded from all analyses because the symptoms began late and were thought to be related to infection acquired in the facility rather than from the primary infection related to inoculation. Gene expression profiles were measured as described for the biological dataset GSE30550.

The biological dataset GSE2565^26^ contained 6 control samples (control group) and 6 case samples (case group). CD-1 male mice were divided into two groups that were exposed to air or phosgene. Lung tissues were collected from air- or phosgene-exposed mice at 0.5, 1, 4, 8, 12, 24, 48 and 72 hours (h) after exposure.

In the biological dataset GSE13268^27^, 50 adipose tissue samples were collected from GK (GotoKakizake) rats fed a normal diet (ND) or a high-fat diet (HFD) that were sacrificed at 5 different ages: 4, 8, 12, 16 and 20 weeks (w). Thus, each time point contained 5 GK samples with ND and 5 GK samples with HFD.

### Protein selection

Student's t-test with a significance level p-value < 0.05 was used to choose genes that showed significant expression changes between asymptomatic (or control) subjects and symptomatic (or case) subjects at each time point. The false discovery rate adjusted p-value < 0.05 was used to correct multiple comparisons or multiple Student's t-tests for the genes that were selected by Student's t-test at each time point (note that this significance level, 0.05, is frequently used in differential expression analysis^47^). The selected proteins for the four datasets are presented in Supplementary Tables S1–S3.

### Construction of dynamic networks

The framework used in construction the dynamic network is shown in Supplementary Figure S1 and mainly included three steps.

#### Construction of an initial network

A rough PPI network was constructed from three PPI databases: Search Tool for the Retrieval of Interacting Genes/Proteins (STRING)^48^, Human Protein Reference Database (HPRD)^49^ and Biological General Repository for Interaction Datasets (BioGRID)^50^. After self-interactions and repeated interactions were filtered, the largest connected component of the rough network was considered the initial network. Finally, we obtained an initial PPI network for the selected proteins in each dataset (Supplementary Figures S3–S5).

#### Detection of noisy interactions

Two interacting proteins always have a temporal relationship between their expression profiles. To filter the interactions with high amounts of noise, we calculated the mutual information (*MI*) for interacting proteins based on their gene expression data. *MI* provides a natural generalization of correlation due to its capability of characterizing non-linear dependency^51^,^52^. For two paired proteins *X* and *Y*, *MI* can be defined as follows^53^:
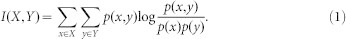


With the widely adopted hypothesis of Gaussian distribution for protein expression, equation (1) can be easily calculated using the following equivalent equation^54^.
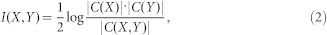
where *C* is the covariance matrix of variable *X* and |*C*| is the determinant of matrix *C*.

A high *MI* score indicates a close relationship between paired proteins, while a low MI score implies protein independence. At each time point, we calculated the *MI*s of the interactions in the initial network based on their gene expression samples respectively for control and case conditions. If the *MI* score is below a given threshold *θ*, the interaction is regarded as a noisy interaction and is deleted for further analysis. Because the *MI* value was only the first discrimination parameter in the overall procedure of gradual refinement, it is necessary to avoid missing any possible PPI pairs during this early stage. The primary aim in this step is to delete only the highly unlikely PPIs. After selecting the possible PPIs, we obtained the refined network in the control and case conditions, respectively.

#### Optimization of the dynamic network

Generally, the dynamic network can be described by the following nonlinear ordinary differential equations:
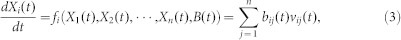
where 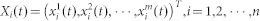 is a continuous vector about time *t*, representing the expression level of a protein *i* (*i* = 1,2,···,*n*) at time *t*, *n* is the number of proteins, and *m* is the number of samples. *v_ij_*(*t*) and *b_ij_*(*t*) denote the reaction and reaction rate (the interaction ability), respectively, from the *j*-th protein to *i*-th protein at time point *t*. Therefore, *B*(*t*) = {b*_ij_*(*t*),*i*,*j* = 1,2,···,*n*} is a parameter set.

The construction of dynamic regulatory networks is to search the optimal to find the set of parameters Ω = {*B*(*t*),*t* = *t*_1_,*t*_2_,···,*t_K_*} in equation (3). This problem can be transformed into the following optimization problem used in finding the set of parameters Ω = {*B*(*t*),*t* = *t*_1_,*t*_2_,···,*t_K_*} to make the simulation results fit the experimental data:
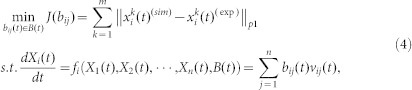
where 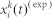 and 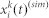 are the experimental and simulation data, respectively, at time point *t* for the *k*-th sample. ||·||*_p_*_1_ is 1-norm or 2-norm.

The optimization problem (4) is a nonlinear dynamic optimization problem (DOPs), which is one of the most challenging problems in the optimization and engineering fields^55^,^56^. For simplicity, we assumed that the interactions among proteins are linear over each time interval [*t_r_*,*t_r_* + Δ*t*] and we used the piecewise linearization to approximate equation (3).
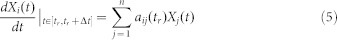
where *a_ij_*(*t_r_*) denotes the interaction ability from the *j*-th protein to *i*-th protein at time point *t_r_*.

In general, the PPI networks are sparse. Therefore, most parameters in equations (5) are zero. Moreover, there are slight changes between two consecutive networks for different time points. Therefore, optimization problem (4) was changed into the following problem:
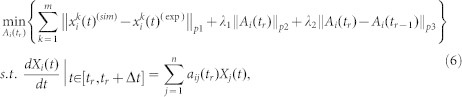
where *A_i_*(*t_r_*) = (*a_i_*_1_(*t_r_*),*a_i_*_2_(*t_r_*),···,*a_in_*(*t_r_*))*^T^* and, ||·||*_p_*_2_ and ||·||*_p_*_3_ are 1-norm or 2-norm. In equation (6), the first term is used to guarantee the accuracy of the optimal parameters of networks, the second term is used to guarantee the sparsity of networks and the third term is used to guarantee the continuity of the dynamic network. *λ*_1_ and *λ*_2_ are regularization parameters that are used to balance the accuracy, sparsity and continuous terms in the objective function. Here, we set ||·||*_p_*_1_, ||·||*_p_*_2_ and ||·||*_p_*_3_ at 1-norm.

Cubic spline interpolation was applied to the microarray data of each sample to obtain data containing the desired samples. Then, the derivative values could be substituted with the central difference for convenience.

By letting 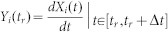, the problem (6) could be approximately transformed into the following problem:
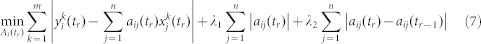


Let
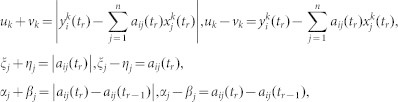
where *k* = 1,2,···,*m*, *j* = 1, 2,···,*n*. *u_k_*, *v_k_*, *ξ_j_*, *η_j_*, *α_j_*, *β_j_* ≥ 0. Then, problem (7) can be written as a standard linear programming (LP) problem.
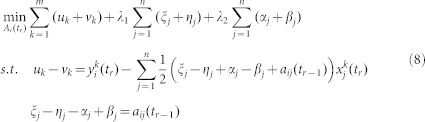


The above LP problem (8) could be solved using MATLAB's linprog function.

#### Selection of regularization parameters λ_1_ and λ_2_

The regularization parameters *λ*_1_ and *λ*_2_ in model (8) trade off sparseness and continuity versus the precision of the resulting dynamic networks. On the one hand, setting *λ*_1_ to zero most likely results in a complete network. As *λ*_1_ increases, fewer edges are recovered until an empty network is reached for larger values of *λ*_1_.Therefore, small values of *λ*_1_ favor recall whereas larger values favor precision of the recovered edges. On the other hand, larger values of *λ*_2_ degenerate the problem of constructing a dynamic network into a static network in which all parameters are equal to each other at every time point. By setting *λ*_2_ to zero, model (8) is transformed into a set of independent *l*_1_-regularized logistic regression problems^57^. Therefore, it is important to select proper *λ*_1_ and *λ*_2_ from the different candidates. Here, we used the Bayesian information criterion (*BIC*) score to optimize *λ*_1_ and *λ*_2_. The optimization problem can be formulated as follows:
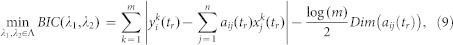

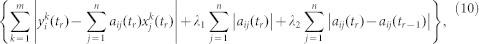
where Λ = {*α*_0_, *α*_1_,···, *α_L_*},*α_i_* = *α*_0_*ρ*,0 < *ρ* < 1, *Dim*(·) denotes the dimensionality of the estimated values. We adopted the following definition, which counts the number of runs of nonzero parameter values:
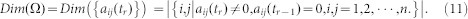


The whole procedure for solving this optimization problem is presented as follows:

First, for a given *λ*_1_,*λ*_2_∈Λ, *a_ij_*(*t_r_*) is identified by solving optimization problem (10). Second, by substituting the identified *a_ij_*(*t_r_*) into (9), we could obtain a *BIC* score. Finally, we obtained the optimal *λ*_1_,*λ*_2_ by minimizing *BIC*.

The detailed steps for calculating the two parameters *λ*_1_ and *λ*_2_ are presented as follows:

Step 1. For each protein in the network, the regularization parameters *λ*_1_ and *λ*_2_ were selected from the set {10^−5^, 10^−4^, 10^−3^, 10^−2^, 10^−1^} using the BIC score.

Step 2. The average values of *λ*_1_ and *λ*_2_ of all proteins were calculated and denoted by 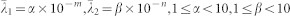


Step 3. For every protein in the network, the regularization parameters *λ*_1_ and *λ*_2_ were again selected from the set {1 × 10^−*m*^, 2 × 10^−*m*^, 3 × 10^−*m*^,···,9 × 10^−*m*^ } for *λ*_1_ and {1 × 10^−*n*^, 2 × 10^−*n*^, 3 × 10^−*n*^,···,9 × 10^−*n*^} for *λ*_2_ using the BIC score.

Step 4. The average values for the two parameters were selected.

#### Determination of significant interactions

The selection of the significant interactions in the resulting dynamic networks is very important. A large threshold value *θ* of the variable parameter *a_ij_*(*t_r_*) will result in dynamic networks with fewer nodes and edges. This study investigated the relationship between the number of nodes and edges and the threshold value *θ* at the first time point for control (asymptomatic) and case (symptomatic) samples from four datasets, which are displayed in Supplementary Figures S6–S9. If the model residual was no longer significantly reduced when *θ* was less than θ*, we chose the threshold value *θ* as θ*. Therefore, we chose a value of θ* of 0.001 for GSE30550 and GSE52428, and 0.01 for GSE2565 and GSE13268.

### Identification of dynamical network biomarkers

Different from previous studies on the identification of DNBs^9^,^10^,^11^,^12^, we aimed to develop a new strategy for detecting the critical transition and its DNB (or pre-disease module) for a complex disease by combining the constructed dynamic networks and the identified dynamic network modules. The computational framework of DNB identification is shown in Supplementary Figure S2, which mainly included four steps.

#### Detection of dynamic network modules

First, we identified the dynamic network modules from the constructed dynamic network using module detection methods, such as the ClusterONE algorithm^28^. Specifically, we employed this algorithm to identify significantly inter-connected clusters of nodes in the control (asymptomatic) and case (symptomatic) networks at each time point (Figure 1d).

#### Discovery of conserved modules

Network modules showing conservation through evolutionary time are likely to reflect well preserved 'core' functions that are maintained by natural selection^58^,^59^. We defined the similarity score of two modules, *M_i_* and *M_j_*, denoted by *SS*(*M_i_*, *M_j_*), to identify conserved modules (i.e., permitting the rewired structural difference of a module possibly representing the same biological function) across multiple conditions and time points as follows:
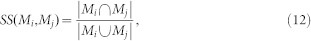
where 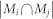 is the number of elements in the intersection of two modules *M_i_* and *M_j_*, and 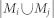 is the number of elements in their union set. We considered two modules as the same module when the *SS* was larger than a threshold *p*. As the functional modules were highly overlapped, *p* was generally set from 0.5 to 0.8^28^,^60^,^61^. The discovery of conserved modules was only the first step in the overall procedure. Therefore, it was necessary to avoid missing any possible conserved modules at this early stage, and we set *p* to 0.5.

If *T* = {*t*_1_,*t*_2_,···,*t_K_*} contained the time points we considered, then the frequency *f_T_*(*M_i_*) of a module *M_i_* was defined as the number of time points at which module *M_i_* appeared. We called module *M_i_* a conserved module if *f_T_*(*M_i_*) = *K*, i.e., the module appeared in all time points. In other words, conserved modules were those that appeared in each time point for both the control and case conditions.

#### Identification of high influence modules

Based on the identified conserved modules, we proposed the new concept of ''high influence modules'' to further quantify the importance of a module in a network. We defined an influence index of a module (*IIM*) as follows:
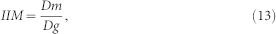
where *Dm* and *Dg* are the average degrees of the module and whole network, respectively. If *IIM* was larger than 1, the module was taken to be a high influence module. In other words, the average degree of the global network was concentrated in this module.

#### Identification of DNBs by composite criterion

According to the previously proposed composite criterion of DNB^9^, we defined the composite criterion for a module as follows:
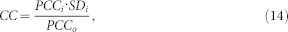
where *PCC_i_* is the average absolute value of Pearson's correlation coefficient (*PCC*) between the expression of molecules within the module across samples within a phenotype; *PCC_o_* is the average absolute value of *PCC* between the expression of molecules inside and outside the module; and *SD_i_* is the average standard deviation (*SD*) of the molecules inside the module across samples within a phenotype. This composite criterion still satisfies the three previously proposed criteria^9^ when the system approaches the critical transition: correlations between the expression of proteins in the same module become stronger; correlations between the expression of proteins from different modules become weaker; and the average standard deviation of protein expressions in one module become larger. Therefore, we could identify the DNBs from high influence modules when the three criteria are satisfied and the composite criterion achieved its largest value in the case condition with no obvious changes in the control condition.

### Calculation of information flow

According to Shannon's information theory, if cellular signaling pathways are considered to be communication channels, the amount of information flow can be quantified by mutual information^40^,^41^. In this study, we defined information flow as global or local.

#### Local information flow

The local information flow (*LIF*) was formulated the same as in equation (1).

#### Global information flow

When cells process information, information is carried through multiple paths. To determine the global influence of input signal on the network response, we defined a global information flow (*GIF*). *GIF* is the weighted sum of *LIF* through each path, where the weight measures the contribution of information transmission through each path to the *GIF*. Accordingly, *GIF* can be formulated as follows:
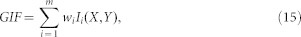
where *m* is the number of output signals, *I_i_* is the local information flow between the input signal (*X*) and the *i*-th output signal (*Y*), and *w_i_* is the weight, which measures the contribution to the global information flow. This can be quantified by the Pearson correlation coefficient (*PCC*) between the input signal and *i*-th output signal. Higher weights had greater contributions. The correlations measured the dependence between the input signal and output signals, and higher correlations indicate relatively greater path importance within the whole network. Thus, we employed the product of the correlation and *LIF* to measure the *GIF*. In this study, we calculated the information flow transmission from the influenza virus (the input signal) to DNBs and 14 other key proteins of interest (the output signals).

## Author Contributions

S.Q.J. and Y.Y.L. contributed equally to this work. Z.S.P. and X.F.Z. designed the study; S.Q.J., L.L. and Y.Y.L. collected and analyzed the data, S.Q.J. and Y.Y.L. performed the research; S.Q.J., Y.Y.L., Z.S.P. and X.F.Z. wrote and revised the manuscript. All authors reviewed the manuscript.

## Supplementary Material

Supplementary Information Supplementary Materials

## Figures and Tables

**Table 1:** Average values of AAEs and AREs for the four datasets

	Dynamic networks in the control conditions		Dynamic networks in the case conditions	
Datasets	AAE	ARE	AAE	ARE
GSE30550	0.0069	0.0009	0.0073	0.0010
GSE52428	0.0534	0.0077	0.0530	0.0077
GSE2565	0.2810	0.0337	0.2817	0.0337
GSE13268	0.3539	0.0400	0.2745	0.0316

**Table 2:** Results of cross-validation for the four datasets

	Dynamic networks in the control conditions					Dynamic networks in the case conditions				
Datasets	SN	SP	FPR	ACC	MCC	SN	SP	FPR	ACC	MCC
GSE30550	0.7329	0.9994	0.0006	0.9987	0.7384	0.7928	0.9994	0.0006	0.9988	0.7997
GSE52428	0.7895	0.9995	0.0005	0.9989	0.7903	0.8435	0.9995	0.0005	0.9990	0.8495
GSE2565	0.9067	0.9984	0.0016	0.9964	0.9147	0.9051	0.9984	0.0016	0.9964	0.9139
GSE13268	0.9298	0.9981	0.0019	0.9965	0.9244	0.9297	0.998	0.0020	0.9965	0.9224

**Table 3:** The identified DNBs are the modules with high influence appearing in both the control and case networks at each time point for the four datasets. The common genes identified by other methods were noted in bold

Datasets	Modules	Genes in the corresponding module
GSE30550	HM1	{**DDX58**; **HERC5**; HERC6; IFI27; **IFI35**; **IFI44**; **IFI44L**; IFI6; **IFIH1**; **IFIT1**; **IFIT2**; **IFIT3**; IRF7;IRF9;**ISG15**;**MX1**;**OAS1**;**OAS2**;**OAS3**; **OASL**; **RSAD2**; XAF1}
GSE52428	HM2	{ADAR; DDX58; GBP1; GBP2; HERC5; HERC6; IFI27; IFI35; IFI44; IFI44L; IFI6; IFIH1; IFIT1; IFIT2; IFIT3; IFIT5; IFITM1; IRF1; IRF7; IRF9; ISG15; MX1; MX2; OAS1; OAS2; OAS3; OASL; RSAD2; RTP4; XAF1}
GSE2565	HM3	{Bnip3; **Esd**; Hk2; Aldoa; Ldha; **Pgd**; Pgk1; Pkm2; **Taldo1**; Tkt; Tpi1;Arhgef12; **Pard6b**; **Prkci**; Rhoj; **Rhou**; Pkp3; **Ppl**; **Scel**; **Gtf2f2**; **Pcf11**; **Papolg**; **Ptbp1**; **Thoc4**; **U2af1**}
GSE13268	HM4	{**Akr1b7**; Fah; Glrx1; Gpx7; Gss; **Gsta3**; Gstm2; **Gstm5**; **Gstm7**; **Gsto1**; **Gsto2**; **Gstp1**; **Gstt1**; **Gstt2**; Mgst2;**Nit1**; **Prdx3**; **Xdh**; **Ankrd6**; Frzb; Fzd1; **Acpl2**; **Lef1**; Nkd1; NlkSox17; C6; Ermp1; **Ppap2c**; **Sgms1**; **Smpd2**; **Smpd3**; **Cyp11a1**; **Cyp11b1**; Tmem50b; Hsd11b2; Fdx1l}

**Table 4:** Comparisons of the sizes of the DNBs identified by different methods. “—” indicates that the results were not calculated by this method. Method 1 is from the literatures^9^ and Method 2 is from the study^10^

	Size of DNBs		
Datasets	Our method	Method 1	Method 2
GSE30550	22	143	22
GSE52428	30	--	--
GSE2565	25	220	--
GSE13268	36	104	--
