# Supplementary material for: Deciphering deterioration mechanisms of complex diseases based on the construction of dynamic networks and systems analysis
Source: Sci Rep. 2015 Mar 19;5:9283. doi: 10.1038/srep09283 (PMC4365388; doi:10.1038/srep09283)
Supplement: Supplementary Information — Supplementary Materials [file srep09283-s1.pdf]

Supplementary Materials:

Deciphering deterioration mechanisms of complex diseases based on the construction of dynamic networks and systems analysis

Yuanyuan Li[1], Suoqin Jin[1], Lei Lei[2], Zishu Pan[2], Xiufen Zou[1]

[1]School of Mathematics and Statistics, Wuhan University, Wuhan 430072, China.

[2]State Key Laboratory of Virology, College of Life Sciences, Wuhan University, Wuhan 430072, China

---

Table of Contents

Supplementary Text......2
Calculation of average absolute and relative errors......2
Cross-validation......2
Supplementary Figures......4
Figure S1, Figure S2......4
Figure S3, Figure S4......5
Figure S5, Figure S6......6
Figure S7, Figure S8......7
Figure S9......8
Figure S10......9
Figure S11......10
Figure S12......11
Figure S13......12
Figure S14, Figure S15......13
Figure S16......14
Figure S17......15
Figure S18......16
Figure S19......17
Figure S20......18
Figure S21......19
Figure S22......20
Figure S23......21
Figure S24, Figure S25......22
Figure S26, Figure S27......23
Supplementary Tables......24
Table S1......24
Table S2......31
Table S3......34
Tables S4-S5......37
Tables S6-S8......38
Tables S9-S12......39
Tables S13-S15......40

Tables S16-S19..........41
Table S20..........42
Table S21..........43
Table S22..........44
Tables S23..........45
TableS24-S25..........46
Table S26..........47
Table S27..........48
Table S28..........49
Table S29..........50
Table S30..........51
Table S31..........52
Supplementary MATLAB source codes...........53

Supplementary Text:

1. Calculation of average absolute and relative errors

In the present study, we defined two types of errors: average absolute error (AAE) and average relative error (ARE), measuring how close predictions were to the experimental data, namely the accuracy of the constructed dynamic networks. Because the absolute error relates to the dimension, it is generally a reference value. The relative error is not related to the dimension, therefore, it can reflect the difference between simulations and experimental data. In scientific computing, the relative error between the simulations and real data is an important index to prove the effectiveness of the method. The absolute error is the magnitude of the difference between the experimental value and the simulation value, and the relative error is the absolute error divided by the magnitude of the experimental value. Accordingly, AAE and ARE can be defined as follows:

$$AAE = \frac{1}{n}\sum_{i=1}^{n}\frac{1}{m}\sum_{j=1}^{m}\left|X_{ij}^{\exp}(t_r+\Delta t) - X_{ij}^{sim}(t_r+\Delta t)\right|. \quad (1)$$

$$ARE = \frac{1}{n}\sum_{i=1}^{n}\frac{1}{m}\sum_{j=1}^{m}\frac{\left|X_{ij}^{\exp}(t_r+\Delta t) - X_{ij}^{sim}(t_r+\Delta t)\right|}{\left|X_{ij}^{\exp}(t_r+\Delta t)\right|}. \quad (2)$$

In the equations (1) and (2), $X_{ij}^{sim}(t_r+\Delta t)$ and $X_{ij}^{\exp}(t_r+\Delta t)$ are the simulation value and experimental value of the *i-th* protein at time point $t_r+\Delta t$ in the *j-th* sample, respectively. $X_{ij}^{sim}(t_r+\Delta t)$ is calculated according to the following formula:

$$X_{ij}^{sim}(t_r+\Delta t) = X_{ij}^{\exp}(t_r-\Delta t) + 2\cdot\Delta t\cdot\sum_{k=1}^{n}(a_{ik}(t_r)\cdot X_{kj}^{\exp}(t_r)), \quad (3)$$

where n is the number of proteins at time point t_r, m is the number of samples and $a_{ik}(t_r)$ is the optimal solution of the optimization problem (6) in the main text, which denotes the interaction ability of the *k-th* protein to *i-th* protein at time point t_r. Δt stands for the time increment.

Moreover, the average values of AAEs and AREs were computed as follows. First, according to the definitions of the AAE and ARE, i.e. the equations (1) and (2), we calculated the AAE and ARE of the temporary network at each time point. Second, the AAEs and AREs were averaged across all the time points, which gave the errors for the constructed dynamic networks in the control condition and case condition, respectively.

2. Cross-validation

For each dataset, leaving one sample $S_i, i = 1, 2, \cdots, N$ out, we used the remaining $N-1$ samples for repeating the process of the construction of dynamic networks, where N represents the number of the subjects. Based on the reconstructed dynamic networks, we carried out the validation for the excluded sample S_i. Five measures were used to evaluate the results, including Sensitivity (SN), Specificity (SP), False positive rate (FPR), Accuracy (ACC) and Matthews coefficient constant (MCC). Mathematically, they are defined as follows[1,2]:

$$SN = TP / (TP + FN) \tag{4}$$

$$SP = TN / (TN + FP) \tag{5}$$

$$FPR = FP / (FP + TN) \tag{6}$$

$$ACC = (TP + TN) / (TP + FP + TN + FN) \tag{7}$$

$$MCC = \frac{TP \cdot TN - FP \cdot FN}{\sqrt{(TP + FP)(TP + FN)(TN + FP)(TN + FN)}} \tag{8}$$

where TP (true positive) is the number of edges of the reconstructed dynamic network matched by the edges of the original dynamic network, FP (false positive) equals the total number of edges of the reconstructed dynamic network minus TP, TN (true negative) is the number of edges not in both the reconstructed dynamic network and the original dynamic network, and FN (false negative) is the number of edges of the original dynamic network that are not matched by the reconstructed dynamic network.

1. Fawcelt, T. An Introduction to ROC Analysis. *Pattern Recogn Lett* **27,** 861–874 (2006).
2. Zhang, X. *et al.* NARROMI: a noise and redundancy reduction technique improves accuracy of gene regulatory network inference. *Bioinformatics* **29**, 106-113 (2013).

Supplementary Figures:

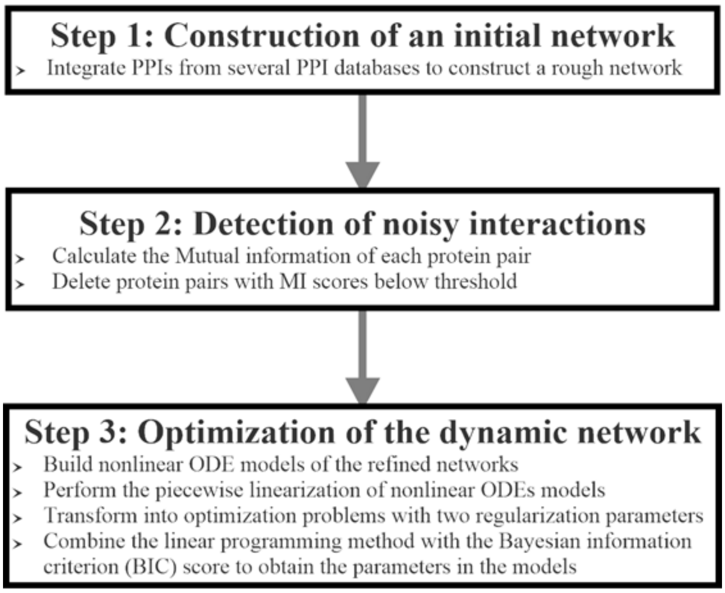

Figure S1 | The framework of the network construction.

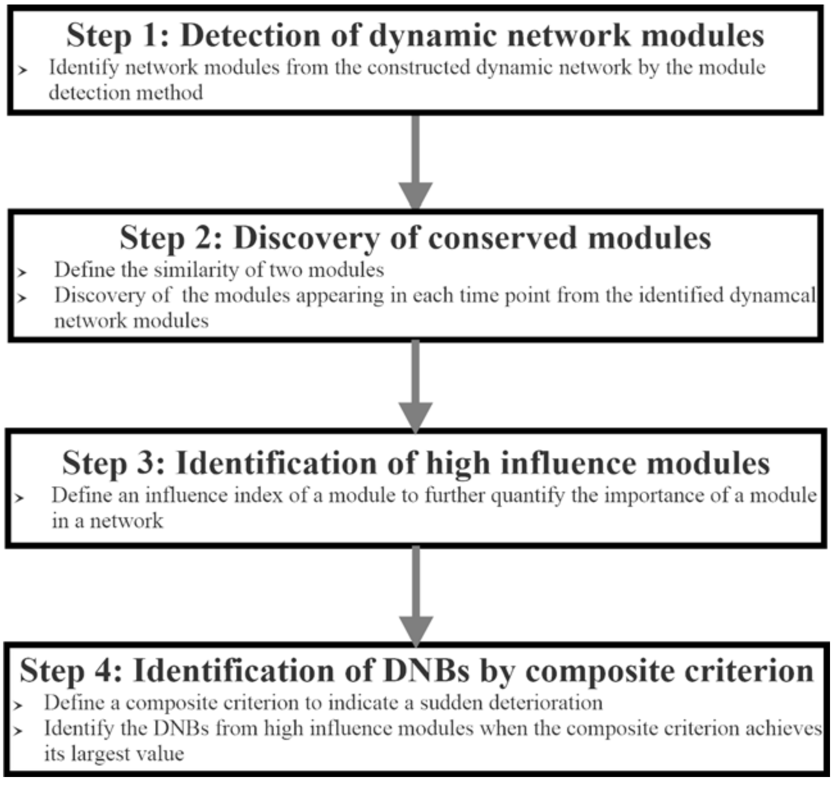

Figure S2 | The framework of dynamic network biomarker identification.

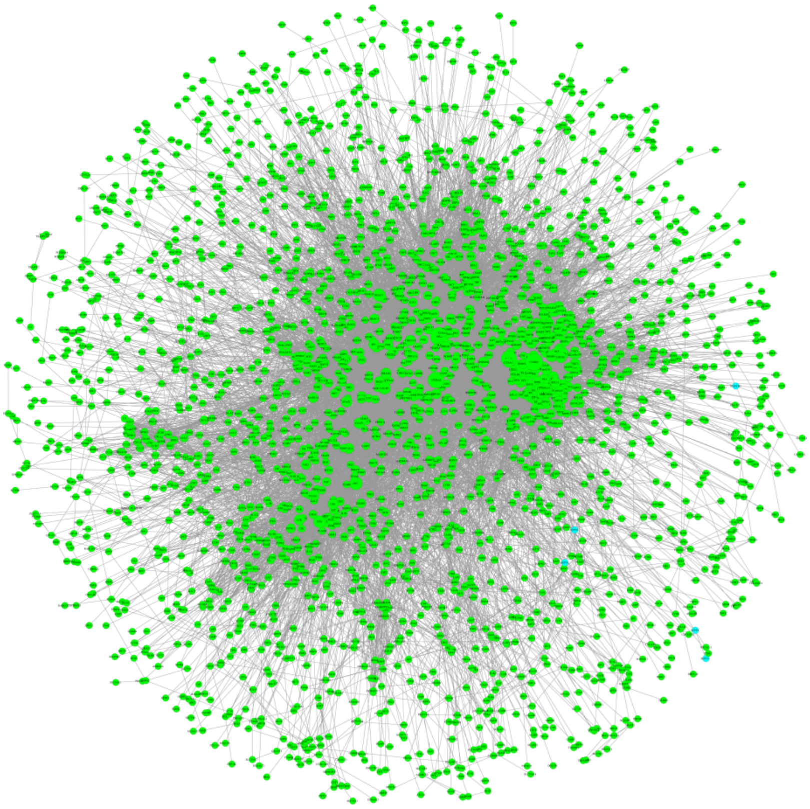

Figure S3 | An initial PPI network collected from three PPI databases (HPRD, BioGRID and STRING) for the datasets GSE30550 and GSE52428. We extracted high confidence (>0.7) interactions from the STRING database. In this network, there are 2400 nodes and 16498 edges. The node sizes are proportional to the degree of the nodes.

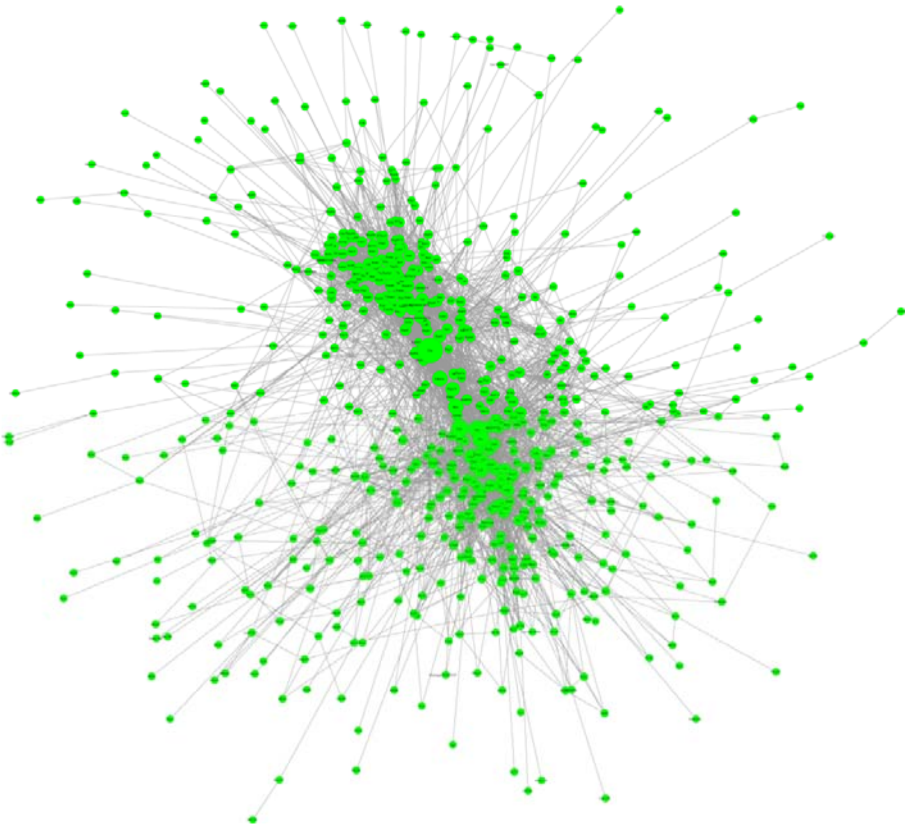

Figure S4 | An initial PPI network collected from three PPI databases (HPRD, BioGRID and STRING) for the dataset GSE2565. We extracted high confidence (>0.7) interactions from the STRING database. In this network, there are 770 nodes and 5890 edges. The node sizes are proportional to the degree of the nodes.

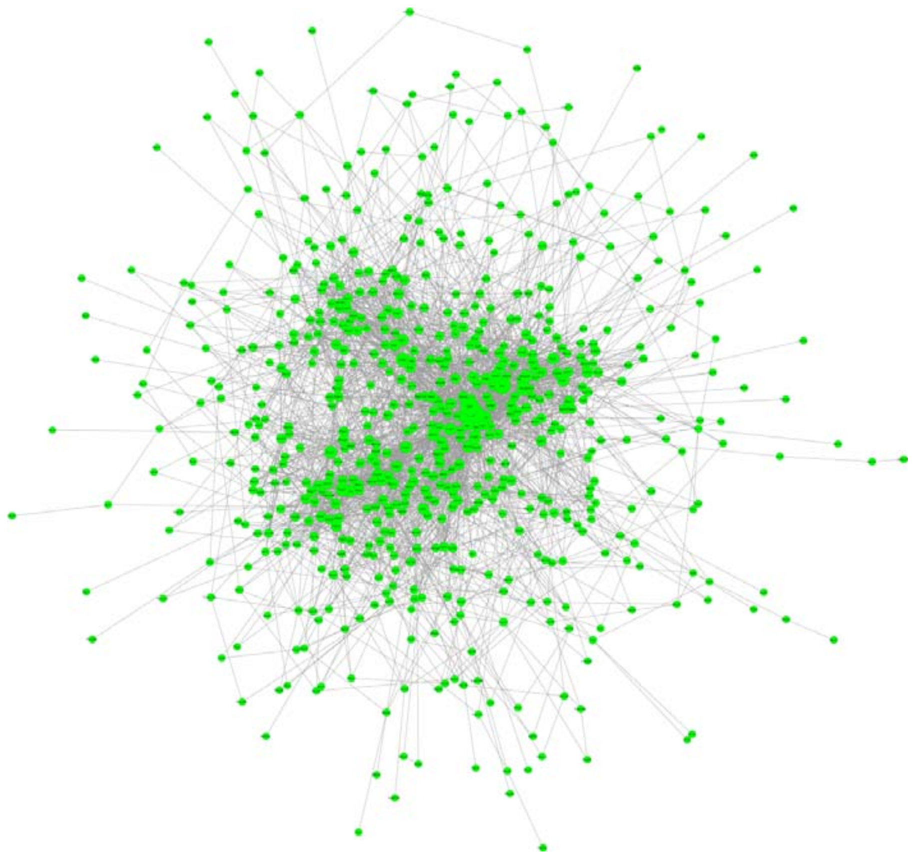

Figure S5 | An initial PPI network collected from three PPI databases (HPRD, BioGRID and STRING) for the dataset GSE13268. We extracted high confidence (>0.7) interactions from the STRING database. In this network, there are 755 nodes and 4054 edges. The node sizes are proportional to the degree of the nodes.

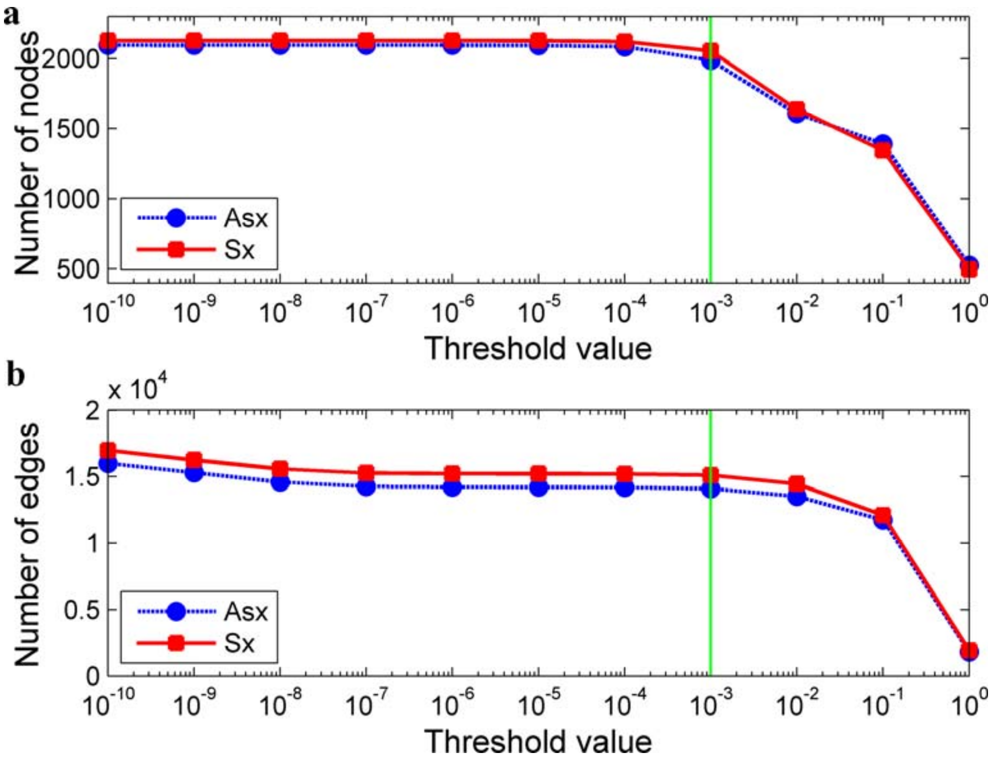

Figure S6 | The number of nodes and edges for various thresholds for the dataset GSE30550. Blue dashed line with circle markers and red solid line with square markers represent the number of nodes (a) and edges (b) in the asymptomatic (Asx) and symptomatic (Sx) networks, respectively. The green line indicates the threshold we chose in our study.

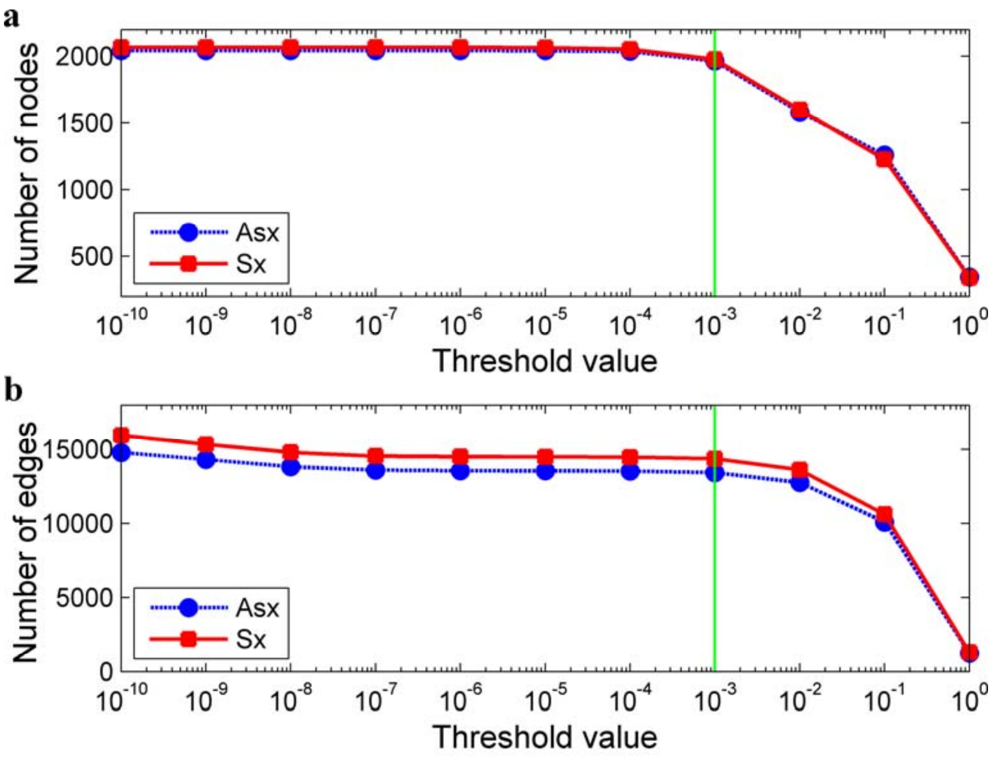

Figure S7 | The number of nodes and edges for various thresholds for the dataset GSE52428. Blue dashed line with circle markers and red solid line with square markers represent the number of nodes (a) and edges (b) in the asymptomatic (Asx) and symptomatic (Sx) networks, respectively. The green line indicates the threshold we chose in our study.

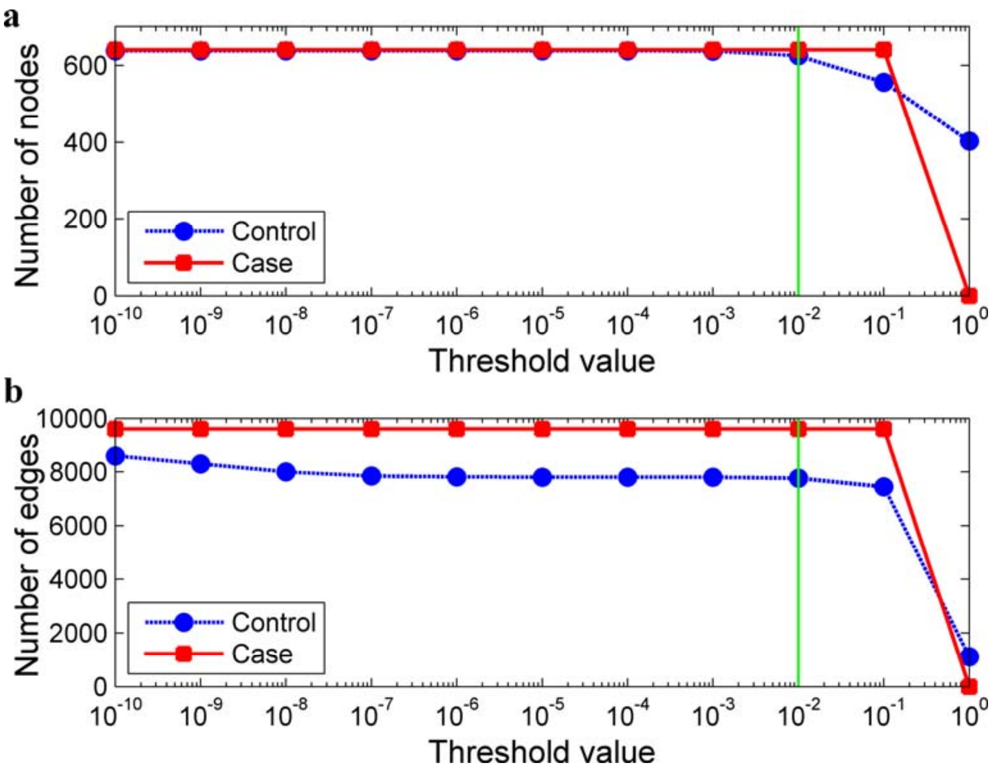

Figure S8 | The number of nodes and edges for various thresholds for the dataset GSE2565. Blue dashed line with circle markers and red solid line with square markers represent the number of nodes (a) and edges (b) in the control and case networks, respectively. The green line indicates the threshold we chose in our study.

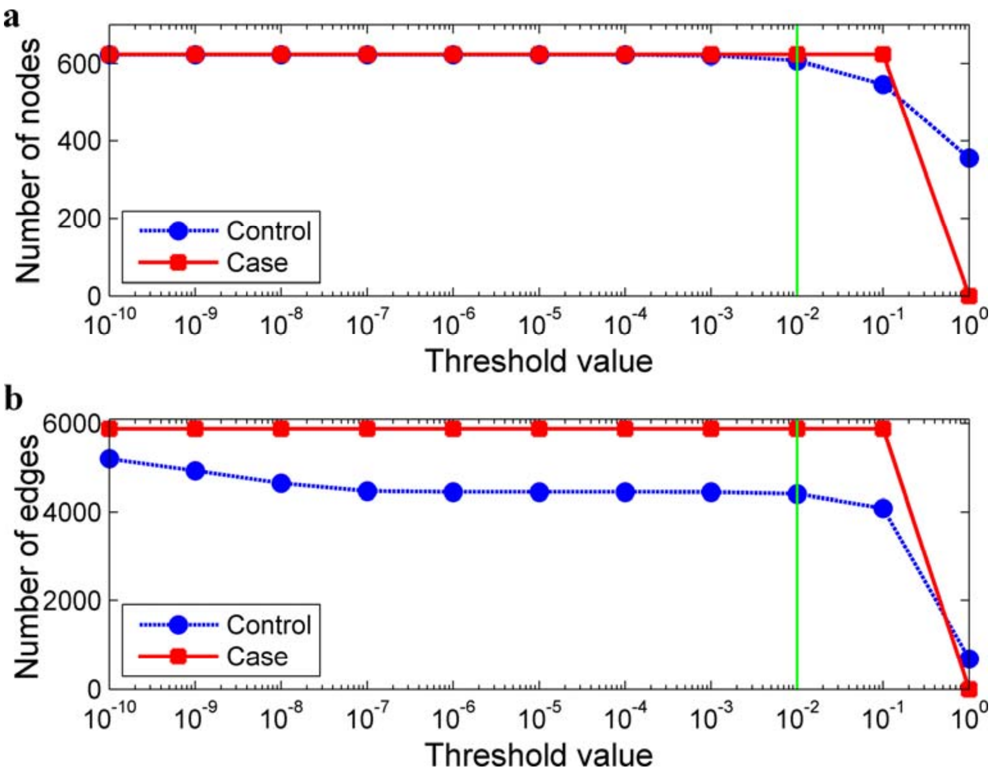

Figure S9 | The number of nodes and edges for various thresholds for the dataset GSE13268. Blue dashed line with circle markers and red solid line with square markers represent the number of nodes (a) and edges (b) in the control and case networks, respectively. The green line indicates the threshold we chose in our study.

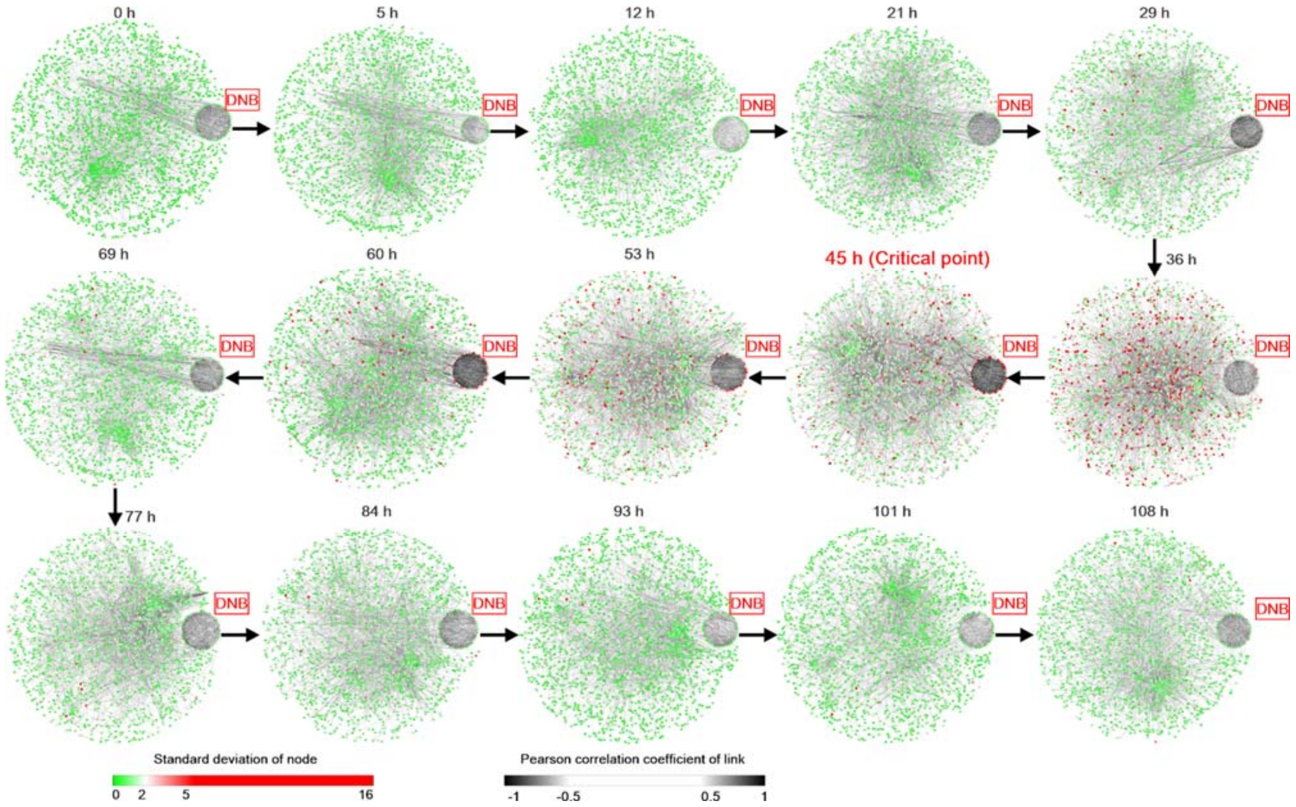

Figure S10 | Dynamical changes in the constructed symptomatic network for the dataset GSE30550, including the detected DNB during the disease progression for the live H3N2 influenza infection. The DNBs are highlighted in the small circle. We show the dynamical evolution of the network structure for the constructed molecular interaction network at 16 time points, i.e. 0h, 5h, 12h, 21h, 29h, 36h, 45h, 53h, 60h, 69h, 77h, 84h, 93h, 101h, and 108h. The color of the nodes represents the fluctuation strength of molecular expressions, and each edge represents the correlation between two nodes. It can be seen that at 45 hour, there is a strong signal to indicate the pre-disease state or pre-symptom. From these figures showing the dynamic evolution, it is obvious that the group of DNB not only provides significant signals as the system approaches the critical point, but also forms a strongly correlated observable subnetwork in terms of expression variations and network connections.

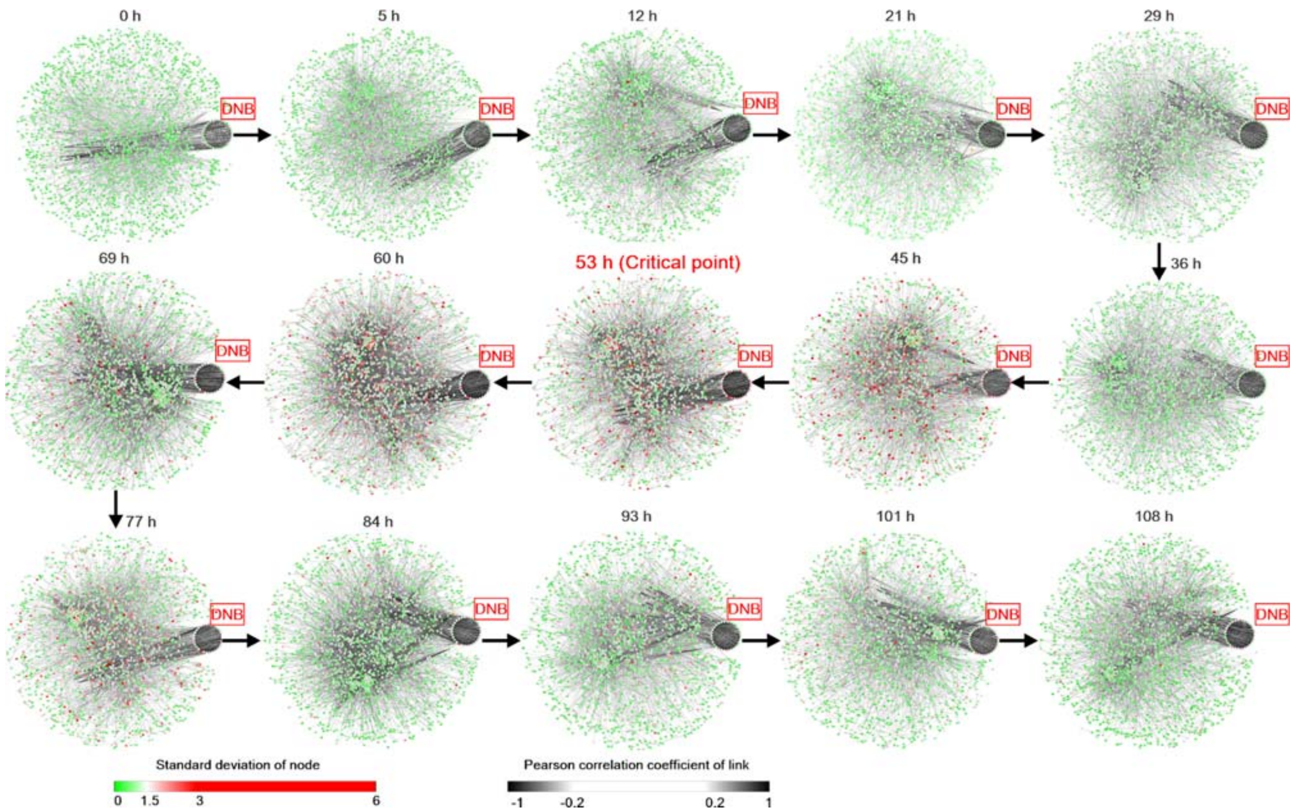

Figure S11 | Dynamical changes in the constructed symptomatic network for the dataset GSE52428, including the detected DNB during the disease progression for the live H1N1 influenza infection. The DNBs are highlighted in the small circle. We show the dynamical evolution of the network structure for the constructed molecular interaction network at 16 time points, i.e. 0h, 5h, 12h, 21h, 29h, 36h, 45h, 53h, 60h, 69h, 77h, 84h, 93h, 101h, and 108h. The color of the nodes represents the fluctuation strength of molecular expressions, and each edge represents the correlation between two nodes. It can be seen that at 53 hour, there is a strong signal to indicate the pre-disease state or pre-symptom.

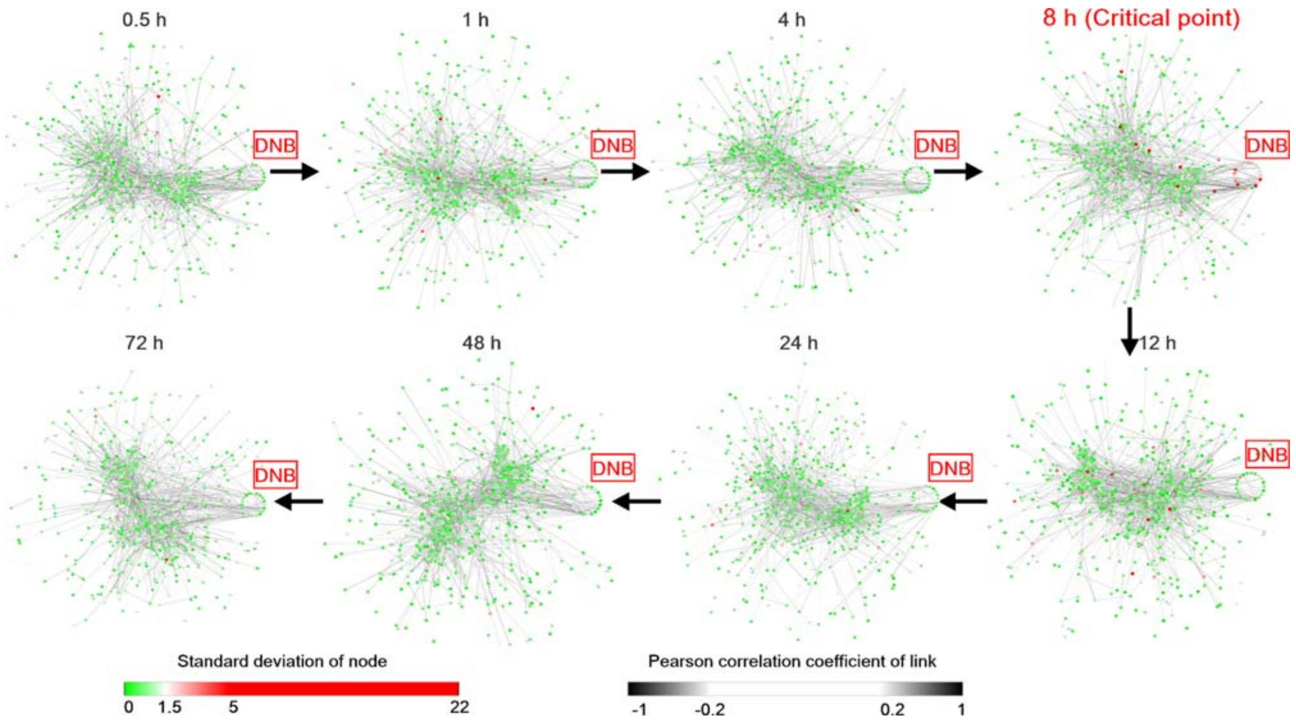

Figure S12 | Dynamical changes in the constructed case network for the dataset GSE2565, including the detected DNB during the disease progression for the acute lung injury. The DNBs are highlighted in the small circle. We show the dynamical evolution of the network structure for the constructed molecular interaction network at 8 time points, i.e. 0.5h, 1h, 4h, 8h, 12h, 24h, 48h, and 72h. The color of the nodes represents the fluctuation strength of molecular expressions, and each edge represents the correlation between two nodes. It can be seen that at 8 hour, there is a strong signal to indicate the pre-disease state.

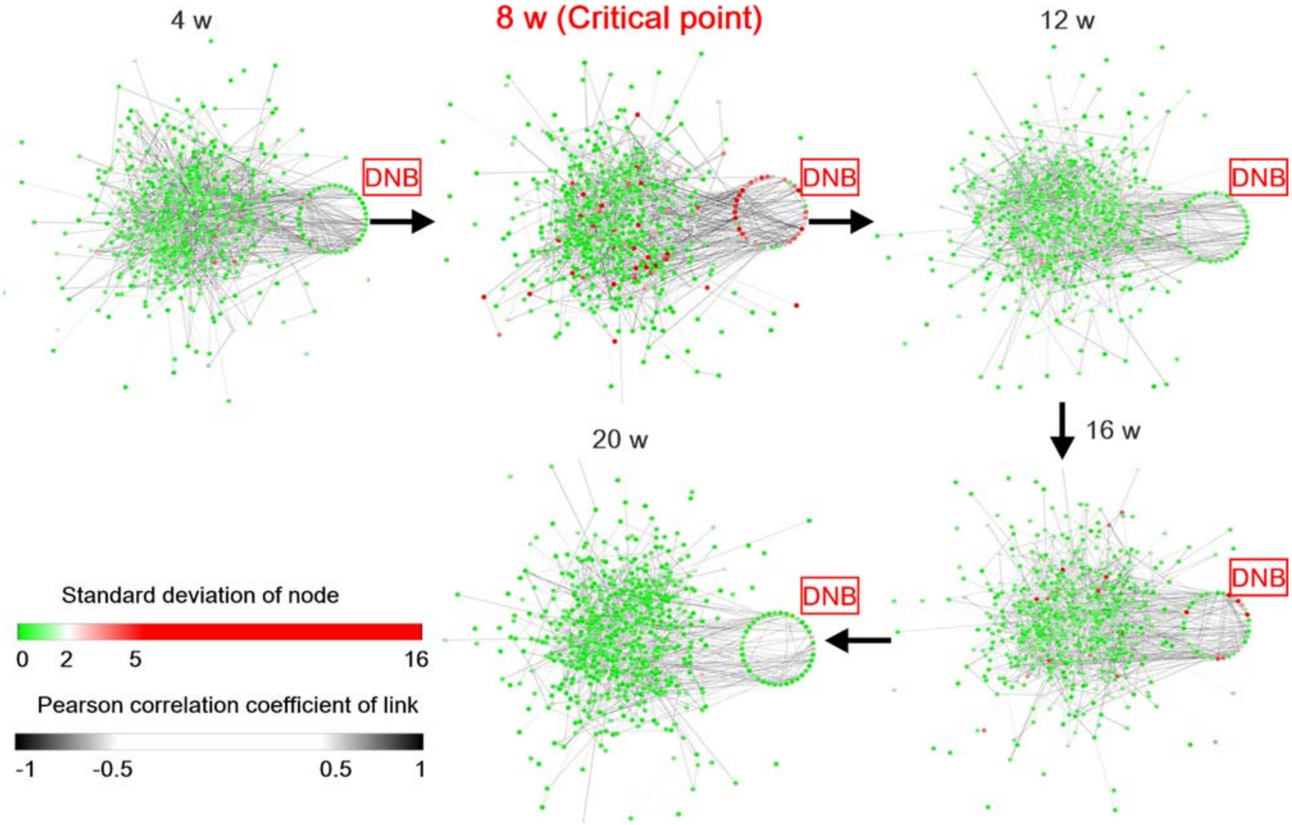

Figure S13 | Dynamical changes in the constructed case network for the dataset GSE13268, including the detected DNB during the disease progression for the type 2 diabetes mellitus. The DNBs are highlighted in the small circle. We show the dynamical evolution of the network structure for the constructed molecular interaction network at 5 time period, i.e. 4 week, 8 week, 12 week, 16 week, and 20 week. The color of the nodes represents the fluctuation strength of molecular expressions, and each edge represents the correlation between two nodes. It can be seen that at the 8 week, there is a strong signal to indicate the pre-disease state.

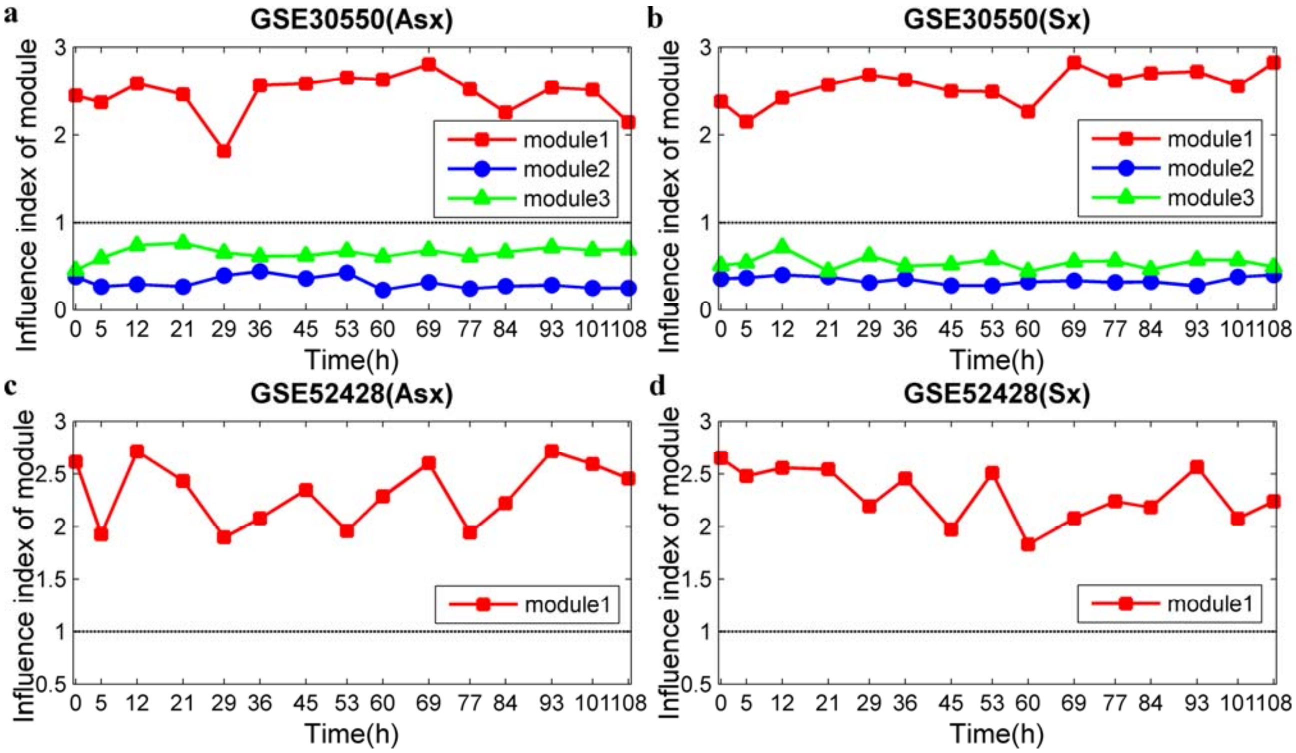

Figure S14 | Influence index of a module (IIM) of common modules respectively for dataset GSE30550 and GSE52428. (a) Three modules in the asymptomatic network (Asx) for GSE30550 dataset. (b) Three modules in the symptomatic network (Sx) for GSE30550 dataset. (c) One module in the asymptomatic network (Asx) for GSE52428 dataset. (d) One module in the symptomatic network (Sx) for GSE52428 dataset.

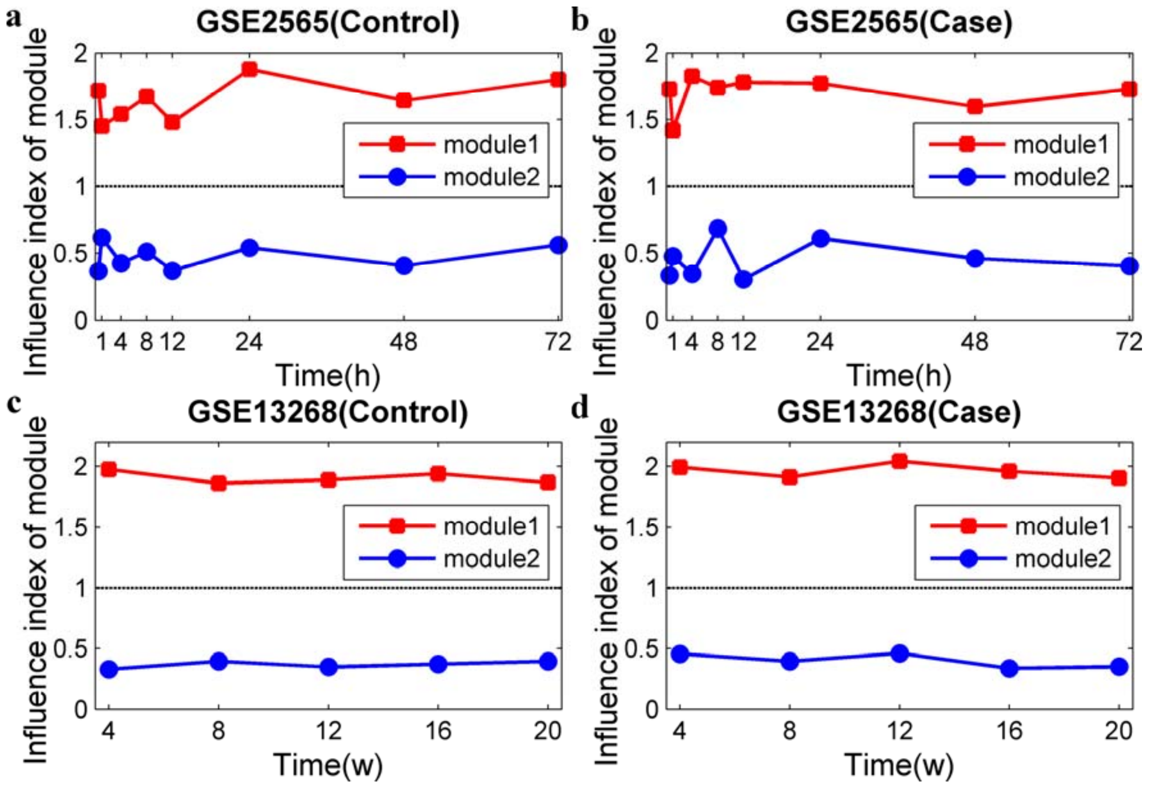

Figure S15 | Influence index of a module (IIM) of common modules respectively for the datasets GSE2565 and GSE13268. (a) Two modules in the control network for the GSE2565 dataset. (b) Two modules in the case network for the GSE2565 dataset. (c) Two modules in the control network for the GSE13268 dataset. (d) Two modules in the case network for the GSE13268 dataset.

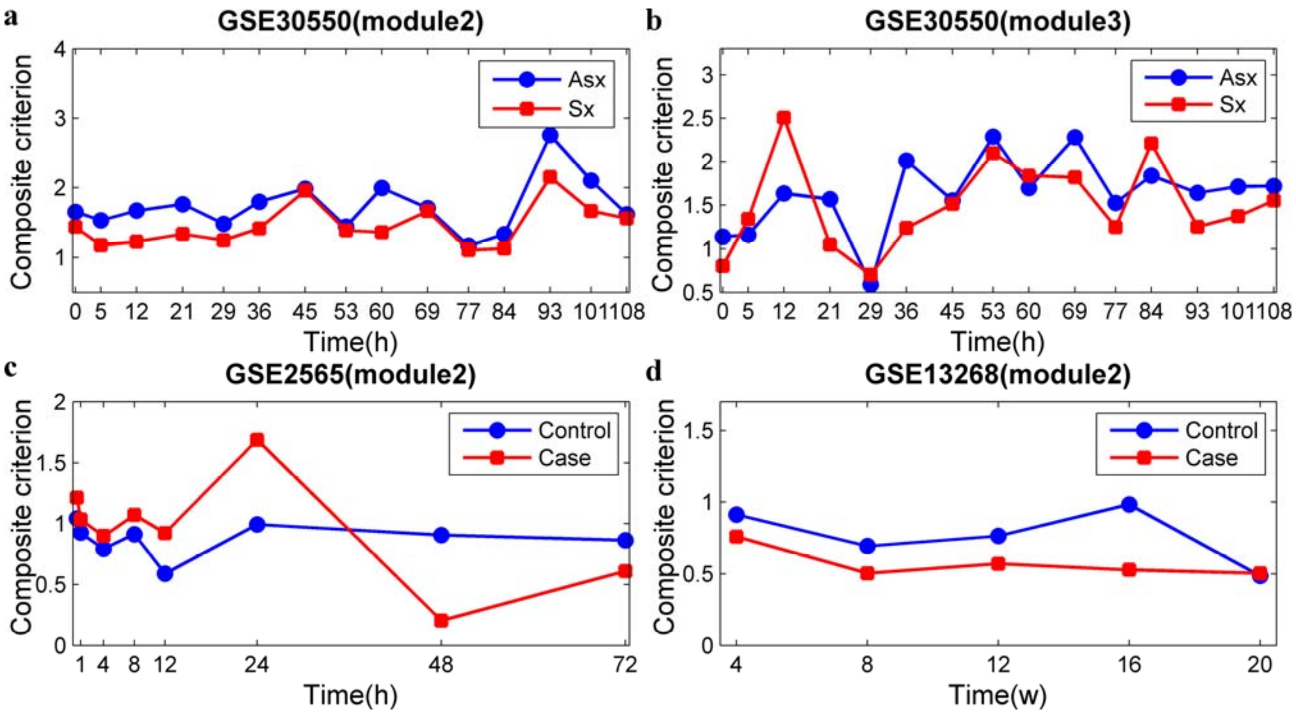

Figure S16 | Comparisons of composite criterions (CCs) of other common modules between the control (Asx) and case (Sx) networks for the four sets of high-throughput experimental data. (a) Module 2 in the GSE30550 dataset; (b) Module 3 in the GSE30550 dataset; (c) Module 2 in the GSE2565 dataset; (d) Module 2 in the GSE13268 dataset. Blue line with circle markers and red line with square markers represent composite criterion of the control (asymptomatic (Asx)) and case (symptomatic (Sx)) networks, respectively. There are no strong and significant early-warning signals of these modules before the diseases are eventually deteriorated.

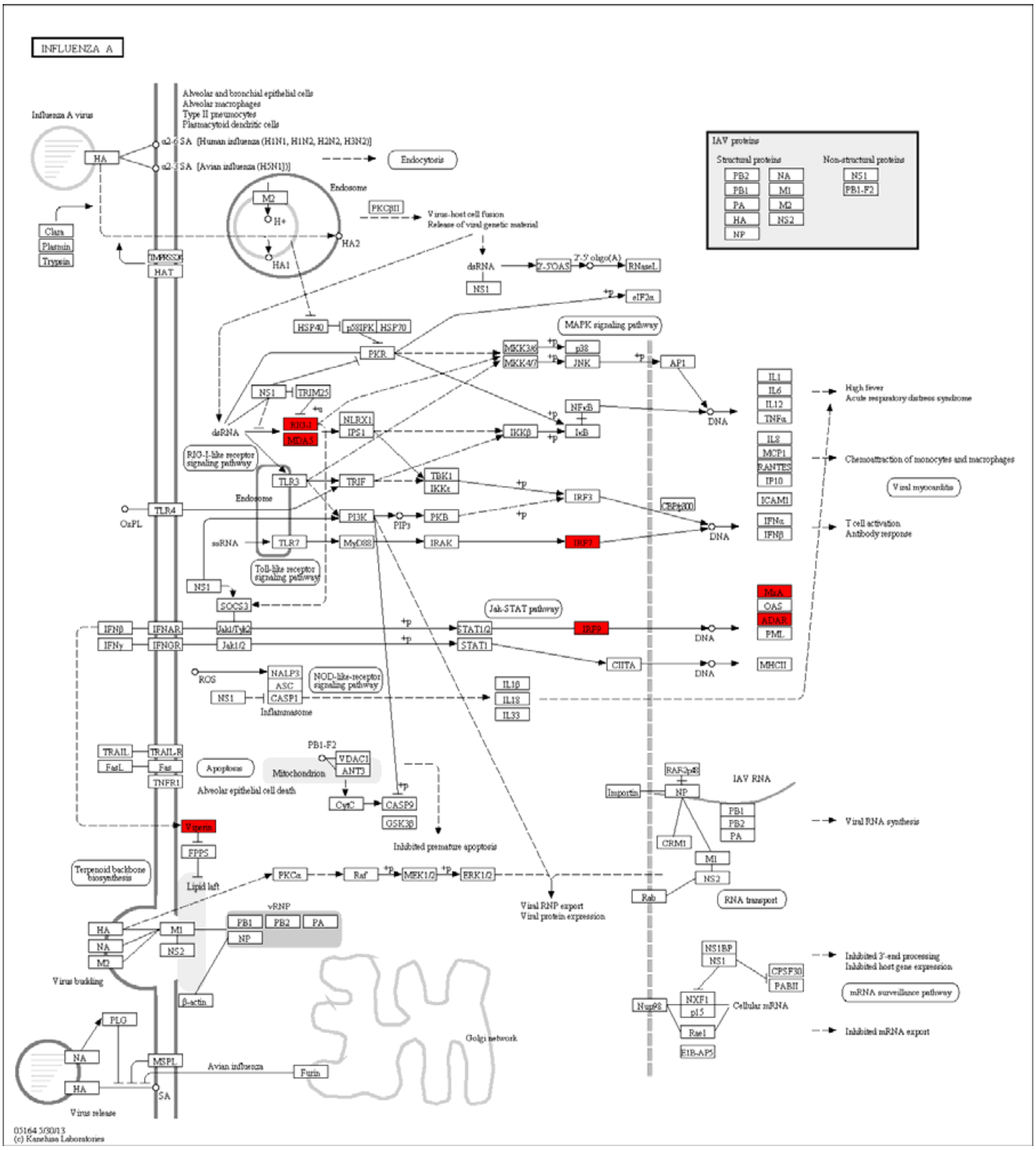

Figure S17 | Influenza A pathway enriched by DNB genes in red color. The red marked genes are DDX58 (RIG-I), MX1, OAS1, OAS2, OAS3, IFIH1 (MDA5) and RASD2 (Viperin).

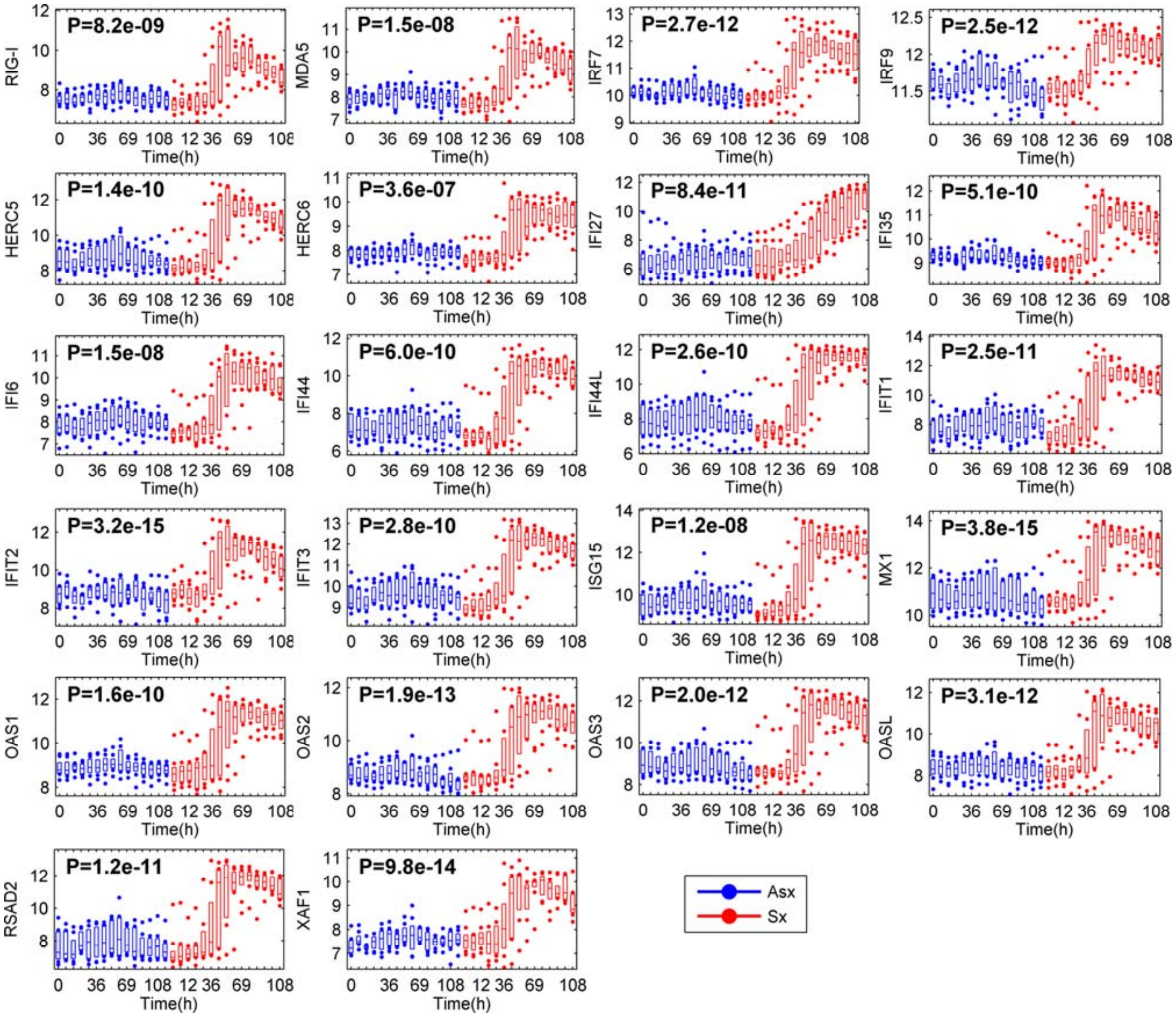

Figure S18 | Comparison of expression values of the identified DNBs in the symptomatic infection (Sx) with those in the asymptomatic infection (Asx) for the H3N2 strain. Blue and red lines indicate Asx and Sx, respectively. P-values are from a two-tailed Wilcoxon rank sum test. The results reveal that DNBs exhibit significant higher levels in the symptomatic infections than those in the asymptomatic infections (P-values < 0.0001).

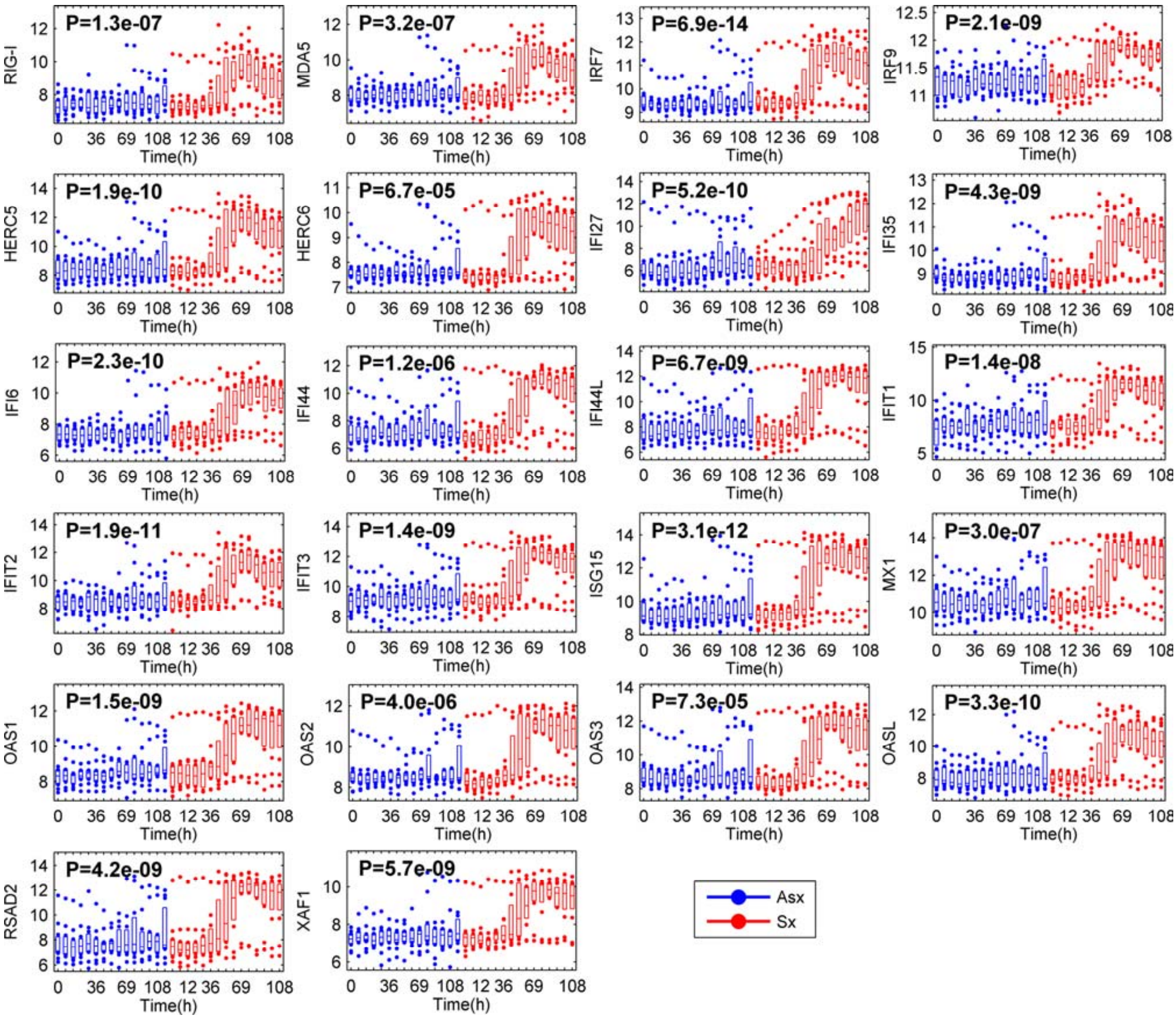

Figure S19 | Comparison of expression values of the identified DNBs in the symptomatic infection (Sx) with those in the asymptomatic infection (Asx) for the H1N1 strain. Blue and red lines indicate Asx and Sx, respectively. P-values are from a two-tailed Wilcoxon rank sum test. The results reveal that DNBs exhibit significant higher levels in the symptomatic infections than those in the asymptomatic infections (P-values < 0.0001).

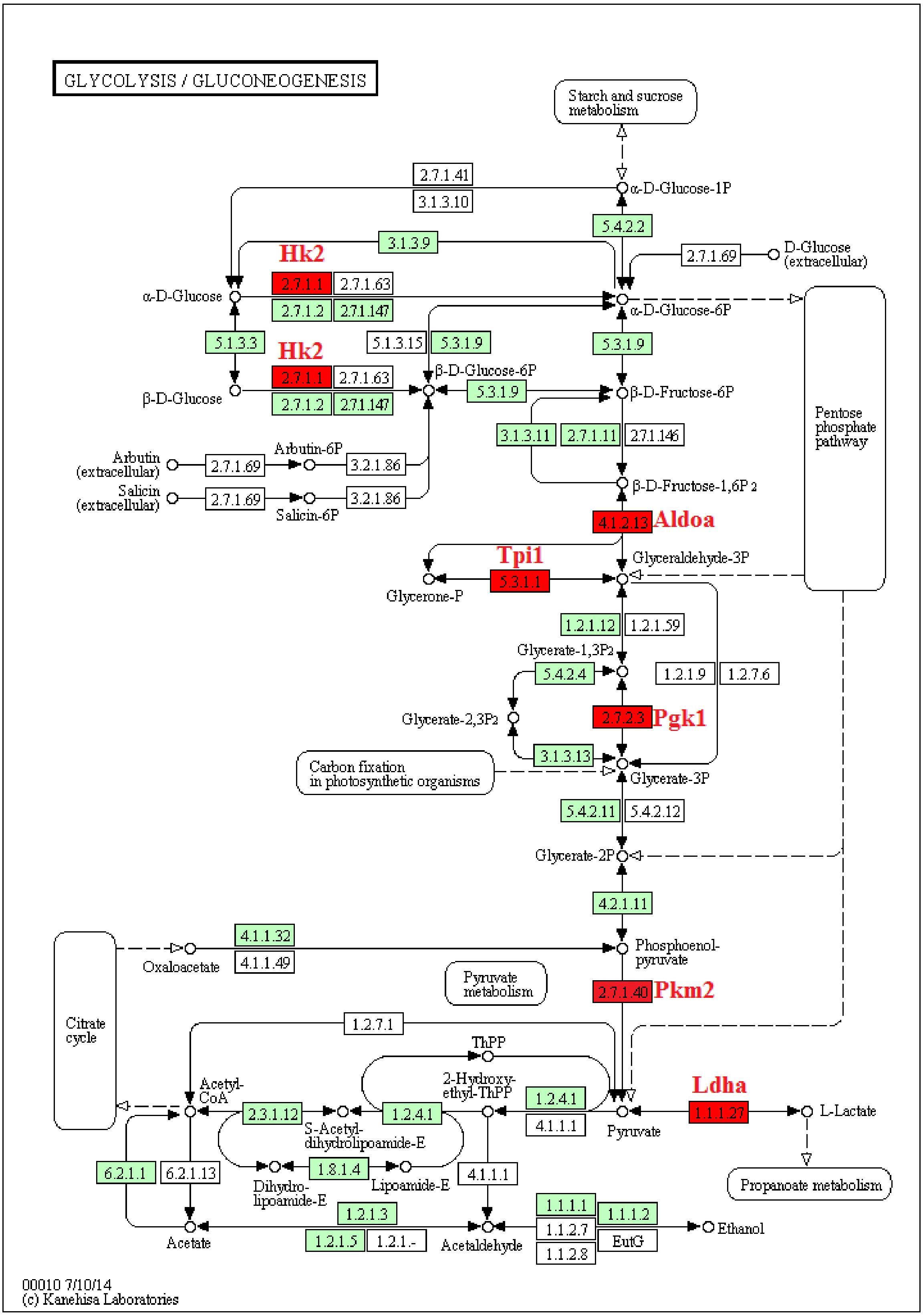

Figure S20 | Glycolysis / Gluconeogenesis pathway enriched by DNB genes in red color. The red marked genes in the figure are Hk2, Aldoa, Ldha, Pgk1, Pkm2 and Tpi1.

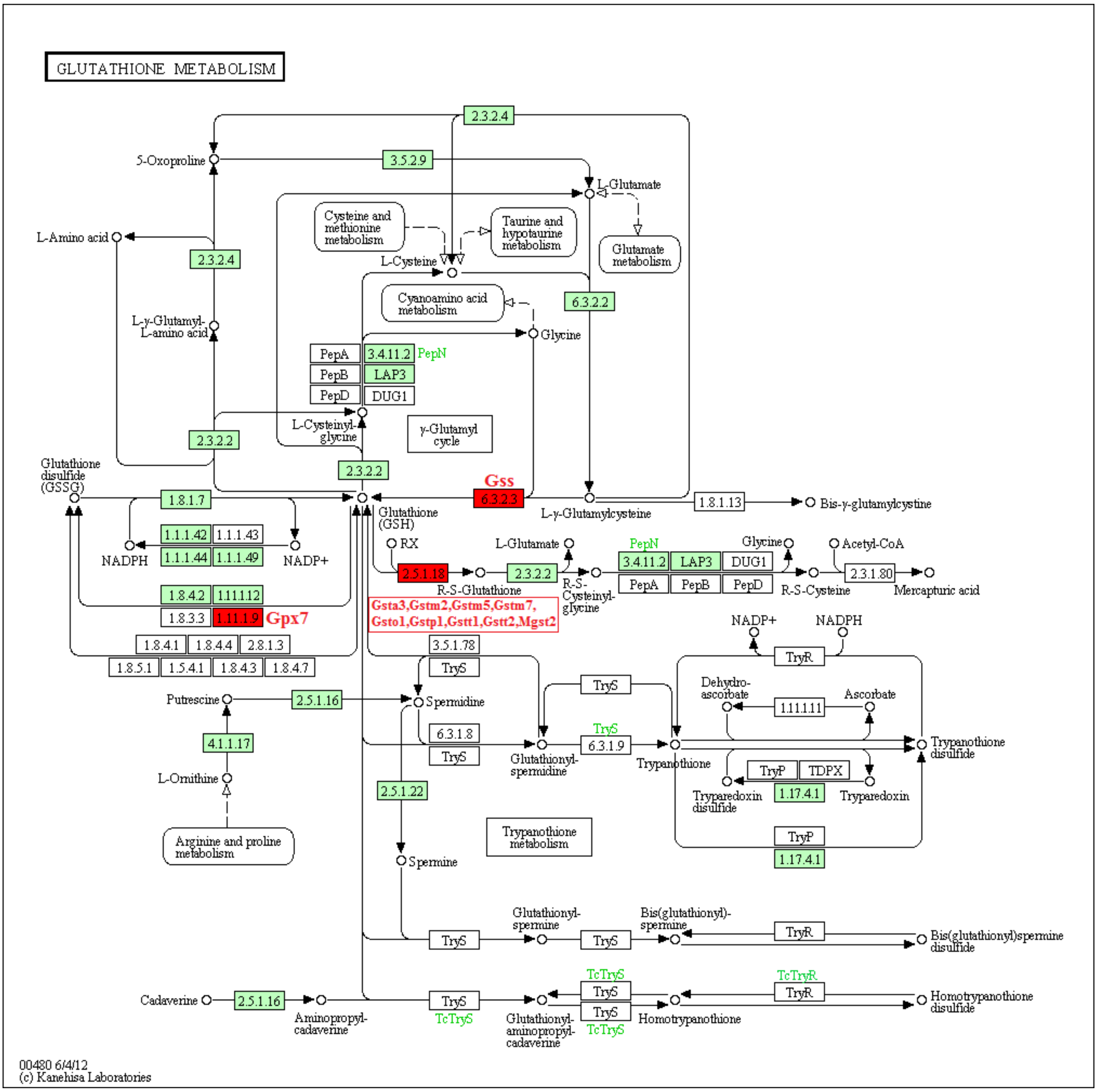

Figure S21 | Glutathione metabolism pathway enriched by DNB genes in red color. The red marked genes in the figure are Gpx7, Gss, Gsta3, Gstm2, Gstm5, Gstm7, Gsto1, Gstp1, Gstt1, Gstt2 and Mgst2.

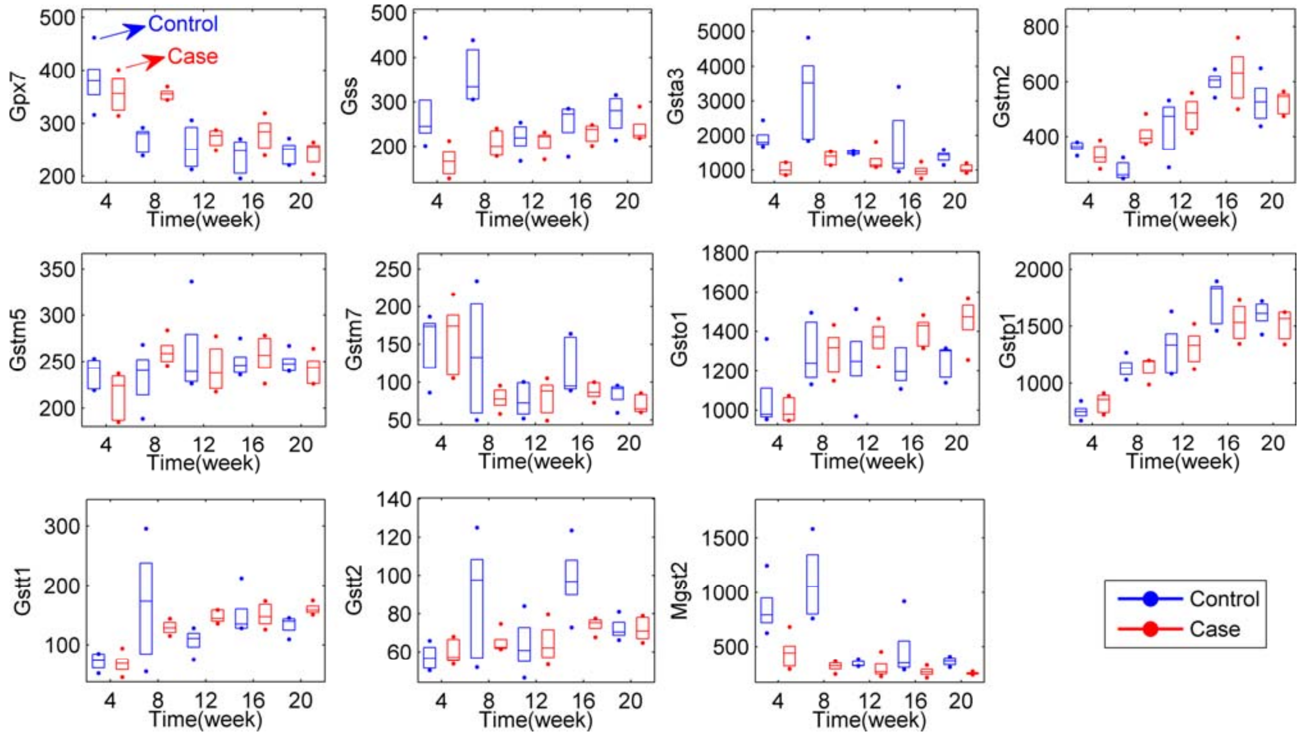

Figure S22 | Comparison of expression values of the DNB genes (enriched in the Glutathione metabolism pathway) in the control with those in the case for the type 2 diabetes mellitus. Blue and red lines indicate control and case, respectively. The results reveal that most of these genes exhibit lower concentrations at almost each period in the diabetic subjects than those in the control subjects, except for Gpx7, Gstm2, Gsto1 and Gstt1.

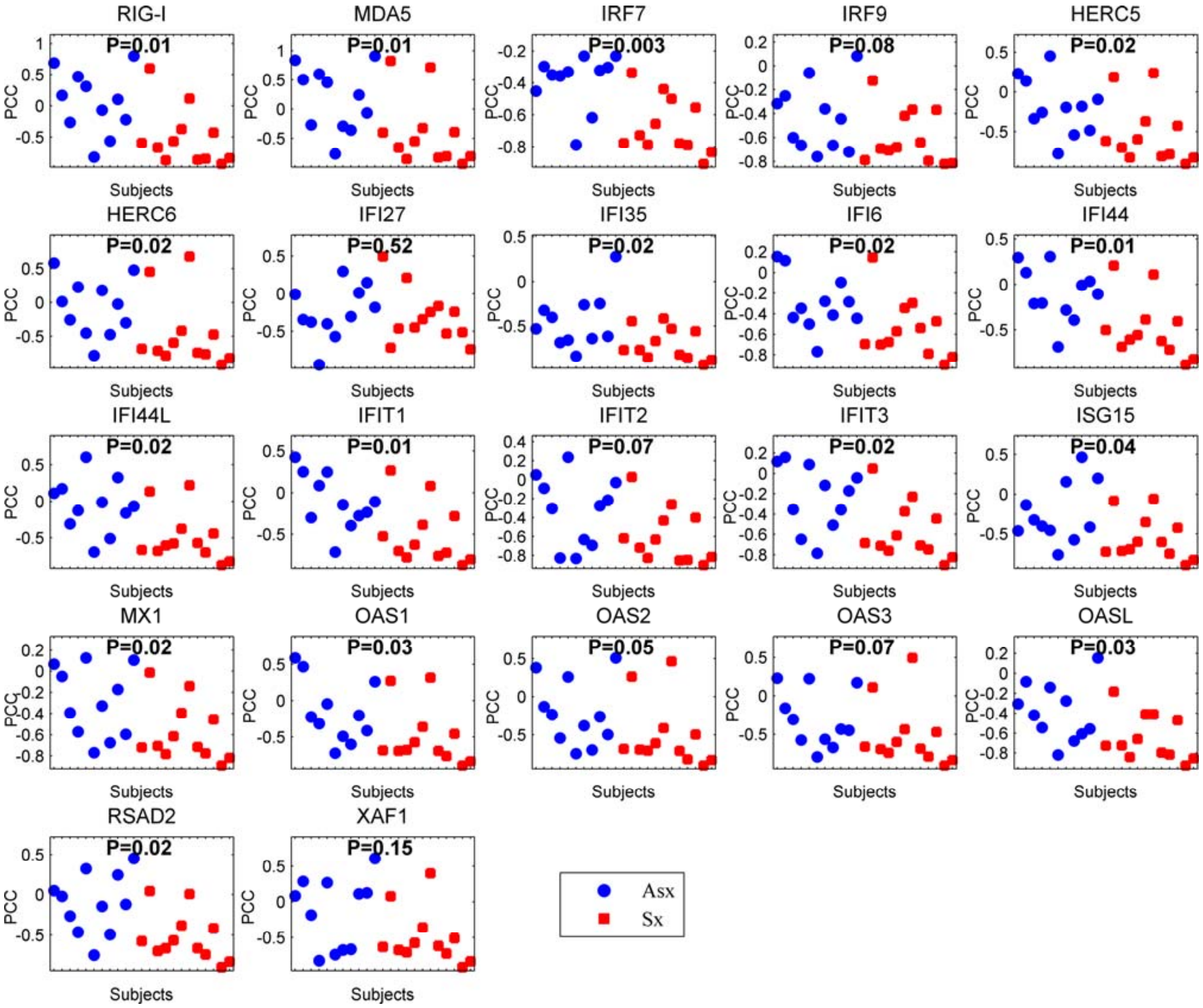

Figure S23 | Comparison of the distributions of correlations between the expression values of S1PR1 and those of DNBs for the H1N1 strain. Blue circles and red squares indicate Pearson correlation coefficient (PCC) between the expression values of S1PR1 and those of DNBs in the asymptomatic infection (Asx) with those in the symptomatic infection (Sx), respectively. X-axis represents the Asx or Sx subjects, namely control or case groups. Each data point corresponds to a correlation in one subject. P-values are from a two-tailed Wilcoxon rank sum test.

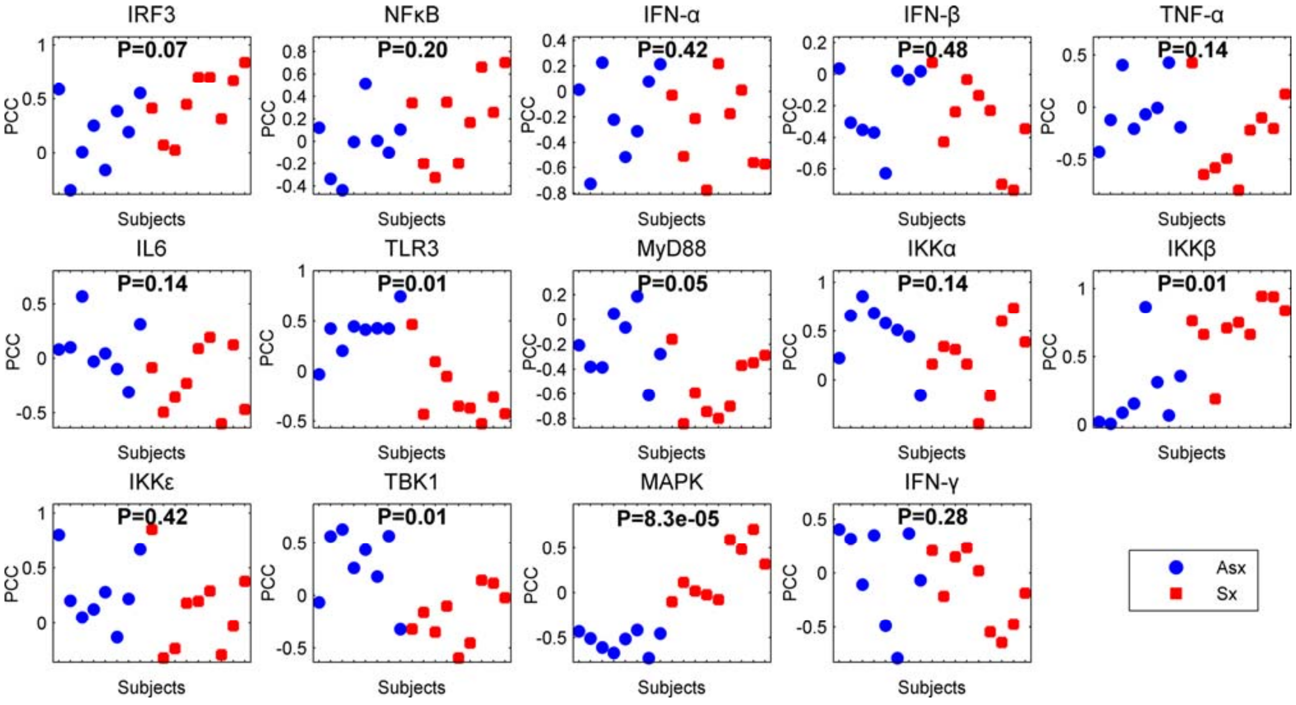

Figure S24 | Comparison of the distributions of correlations between the expression values of S1PR1 and those of other 14 critical proteins for the H3N2 strain. Blue circles and red squares indicate Pearson correlation coefficient (PCC) between the expression values of S1PR1 and those of other 14 critical proteins of innate immunity response in the asymptomatic infection (Asx) with those in the symptomatic infection (Sx), respectively. P-values are from a two-tailed Wilcoxon rank sum test. The difference between the distributions of correlations of the two clinical outcomes was not significant for the H3N2 strain (most of the P-values are larger than 0.05).

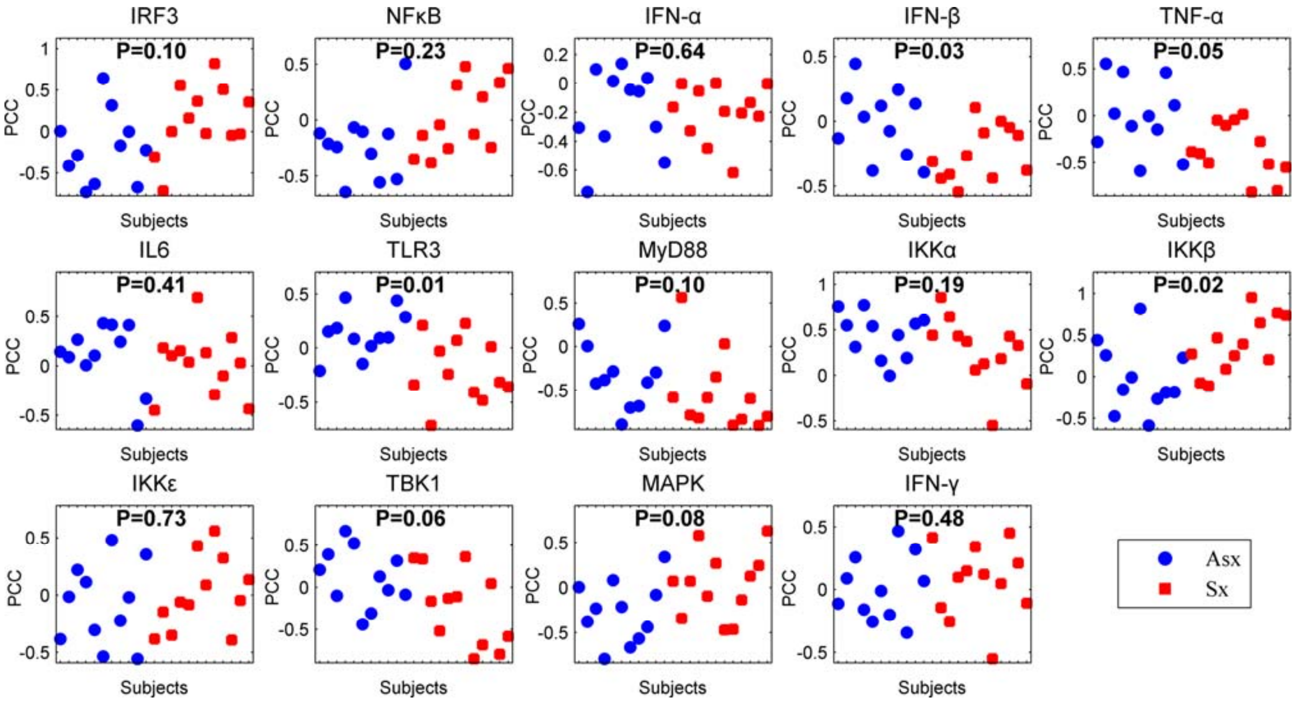

Figure S25 | Comparison of the distributions of correlations between the expression values of S1PR1 and those of other 14 critical proteins for the H1N1 strain. Blue circles and red squares indicate Pearson correlation coefficient (PCC) between the expression values of S1PR1 and those of other 14 critical proteins of innate immunity response in the asymptomatic infection (Asx) with those in the symptomatic infection (Sx), respectively. P-values are from a two-tailed Wilcoxon rank sum test. The difference between the distributions of correlations of the two clinical outcomes was not significant for the H1N1 strain (most of the P-values are larger than 0.05).

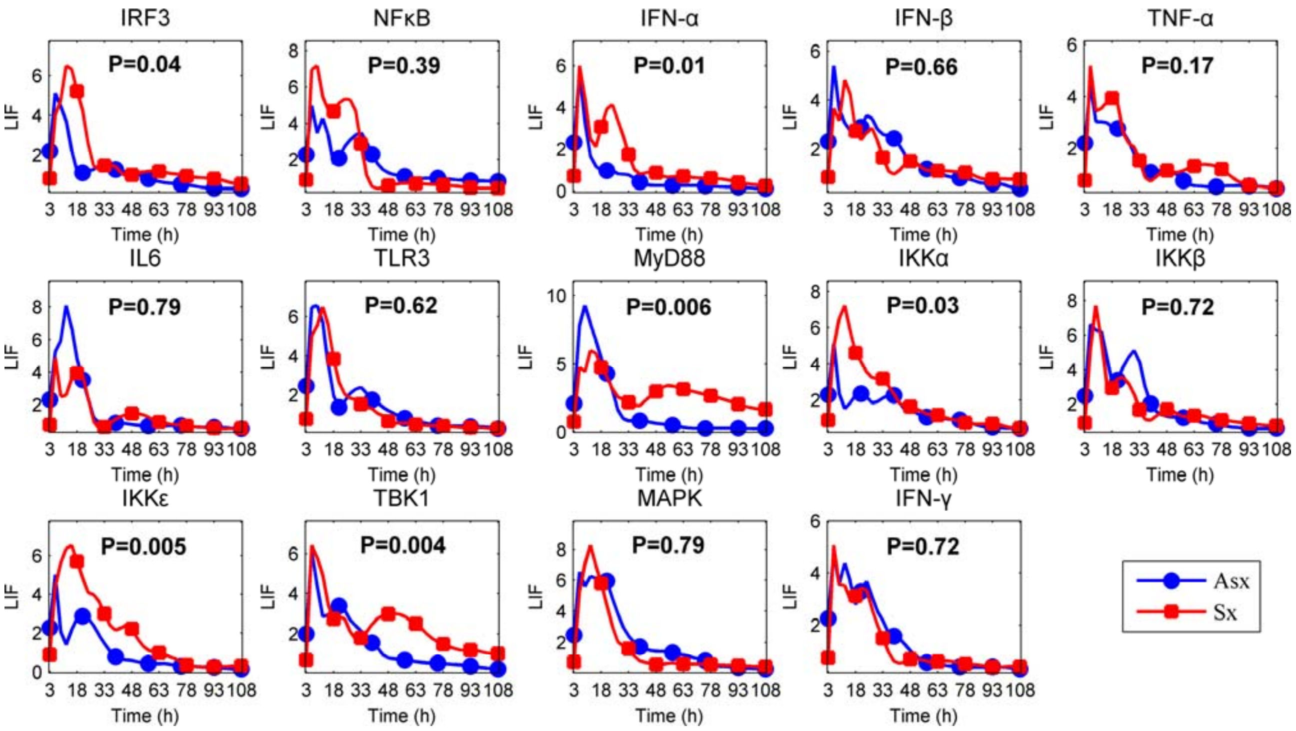

Figure S26 | Comparison of the local information flows (LIF) transmission from virus to other 14 key proteins of innate immunity response in the symptomatic subjects (Sx) and the asymptomatic subjects (Asx) infected by the H3N2 influenza strain. The blue and red lines denote the local information flows in Asx and Sx, respectively. Through performing a two-sample t-test, we showed that the local information flows exhibited no significantly differences between Asx and Sx, except for MyD88, IKKε and TBK1.

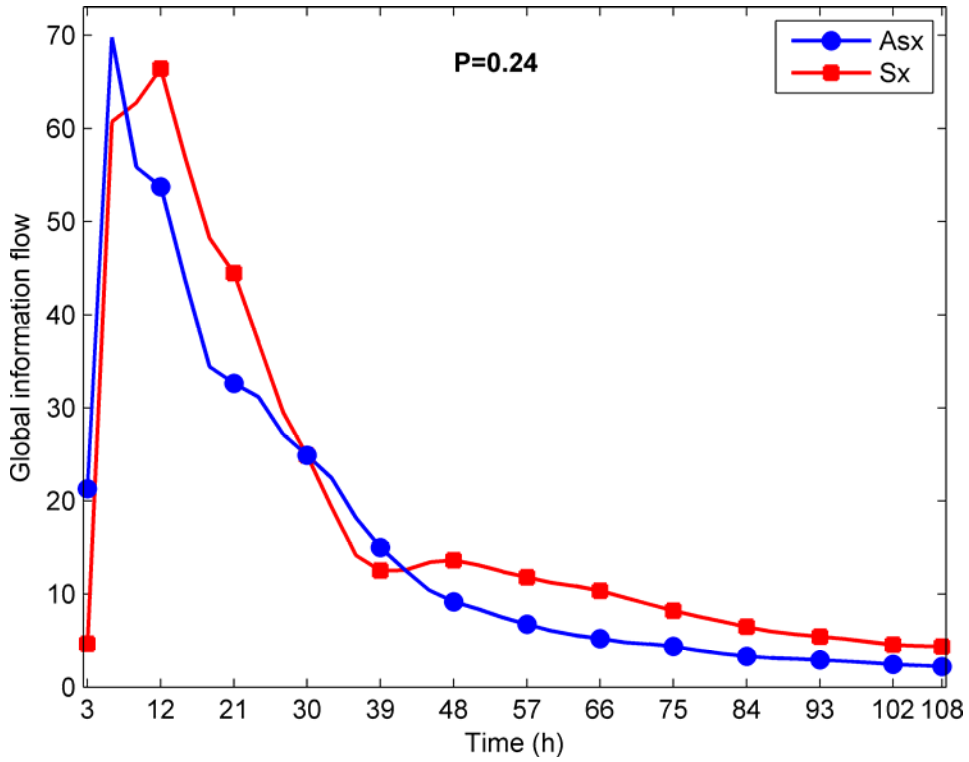

Figure S27 | Comparison of the global information flows transmission from virus to other 14 key proteins of innate immunity response in the symptomatic subjects (Sx) and the asymptomatic subjects (Asx) infected by the H3N2 influenza strain. P-value is from a two-sample t-test. The blue and red lines denote the global information flows in Asx and Sx, respectively. The result showed that there are not significant differences in the global information flows between Asx and Sx (P-value = 0.24).

Supplementary Tables:

Table S1 The 2400 proteins selected in the datasets GSE30550 and GSE52428

Symbol	Symbol	Symbol	Symbol	Symbol	Symbol	Symbol	Symbol
ABCA5	CCBL1	ECHS1	HIST1H4L	MICB	PEX3	RPL8	TCEA2
ABCB1	CCBL2	ECI2	HIST3H3	MICU1	PEX5	RPLP1	TCEAL1
ABCB11	CCDC130	ECM1	HIVEP3	MIF	PFAS	RPLP2	TCEAL4
ABCB4	CCDC33	EDC4	HK3	MIIP	PFKFB3	RPP14	TCERG1
ABCB7	CCDC9	EDEM1	HLA-A	MINA	PFKM	RPS11	TCF20
ABCB8	CCL11	EDEM2	HLTF	MIOS	PFN1	RPS13	TCF25
ABCC1	CCL13	EDF1	HMG20A	MIPEP	PFN2	RPS14	TCF7
ABCC3	CCL2	EDNRA	HMGN1	MIS12	PGAP3	RPS16	TCF7L2
ABCC5	CCL22	EDNRB	HMGN3	MKKS	PGM5	RPS19	TCL1A
ABCD1	CCL25	EED	HMHA1	MKL2	PHACTR2	RPS2	TCN2
ABCD2	CCL5	EEF1D	HMOX1	MKLN1	PHB2	RPS20	TCP1
ABCD4	CCL7	EEF2	HMOX2	MKNK2	PHF10	RPS21	TCP11
ABCE1	CCL8	EFHC1	HN1L	MKRN2	PHF11	RPS23	TDP1
ABHD10	CCNA1	EFHC2	HNF1A	MLH1	PHF15	RPS24	TDRD3
ABLIM1	CCNC	EFNB1	HNRNPA0	MLLT11	PHF16	RPS3	TDRD7
ABLIM3	CCND2	EGF	HNRNPA1	MLLT3	PHF21A	RPS4X	TENC1
ABT1	CCNG1	EGFL7	HNRNPC	MLXIP	PHLDA2	RPS5	TERF1
ABTB2	CCNG2	EGLN2	HNRNPD	MMS19	PHLPP2	RPS6	TESPA1
ACAA2	CCNL2	EGR1	HNRNPDL	MOAP1	PI4KA	RPS6KA2	TEX10
ACACA	CCR1	EGR3	HNRNPK	MOCS2	PIAS2	RPS6KA3	TFAP2B
ACAD8	CCR10	EGR4	HNRNPR	MORC4	PIBF1	RPS6KA5	TFB2M
ACADS	CCR6	EHBP1	HNRNPUL1	MORF4L1	PIK3C2B	RPS9	TFEC
ACAP1	CCR7	EHD4	HOMER1	MPDU1	PIK3R1	RRAS	TGDS
ACAT2	CCSER2	EI24	HOXA3	MPHOSPH10	PIK3R4	RRAS2	TGFB1I1
ACKR2	CCT3	EIF1AX	HOXB2	MPI	PIKFYVE	RRBP1	TGIF1
ACKR3	CCT4	EIF2AK2	HOXC6	MPP5	PILRA	RREB1	TGM4
ACO1	CCT7	EIF2AK3	HPGD	MPRIP	PILRB	RRM1	THADA
ACP2	CD101	EIF2B3	HPSE	MRFAP1L1	PIN1	RRP15	THAP11
ACP6	CD14	EIF2D	HPSE2	MRPL20	PIP4K2C	RRP1B	THBS1
ACTG1	CD163	EIF3A	HR	MRPL24	PJA1	RRS1	THEMIS2
ACTL6A	CD19	EIF3D	HS2ST1	MRPL3	PKIA	RSAD1	THY1
ACTL6B	CD2	EIF3E	HSD11B1	MRPL48	PKMYT1	RSAD2	TICAM1
ACTR1B	CD209	EIF3G	HSD17B12	MRPL9	PKP4	RSBN1	TIMM10
ACTR2	CD22	EIF3H	HSD17B4	MRPS15	PLA2G15	RSL1D1	TIMM22
ACTR3B	CD226	EIF3J	HSD17B8	MRPS2	PLAC8	RSRC1	TIMM9
ACTR5	CD244	EIF3K	HSF2	MRPS27	PLAG1	RTCB	TIMP2
ACTR6	CD247	EIF3L	HSP90AB1	MRPS31	PLAUR	RTN2	TK1
ACYP1	CD27	EIF4A1	HSPA14	MRTO4	PLCB3	RTP4	TLE2
ADAP2	CD28	EIF4A2	HSPA8	MS4A1	PLCG1	RUVBL1	TLR5
ADAR	CD38	EIF4ENIF1	HSPA9	MSH2	PLCL2	RWDD3	TLR6
ADCK3	CD3D	EIF4G2	HSPB11	MSH5	PLEKHA1	RYK	TLR7

ADCY6	CD3E	EIF5	HTATIP2	MSH6	PLEKHA4	RYR2	TLR8
ADD3	CD3G	ELF1	HTATSF1	MSL2	PLEKHA5	S100A4	TLX1
ADH6	CD5	ELF4	HTR7	MSR1	PLEKHB1	S1PR1	TLX2
ADK	CD52	ELOVL2	HYAL2	MST1R	PLIN3	S1PR2	TMA16
ADM	CD6	EMD	IARS	MST4	PLSCR1	SACS	TMC6
ADNP	CD68	EMG1	IARS2	MT1G	PLTP	SAE1	TMCC2
ADRA1A	CD7	EML3	ICAM1	MT1X	PLXNB2	SAMD4A	TMED10
ADRA1D	CD72	EML4	ICK	MT2A	PLXNB3	SAMD9	TMED3
ADRA2C	CD79A	ENO2	ICOS	MTA1	PML	SAMHD1	TMEM62
ADRBK2	CD79B	ENO3	ID3	MTCH1	PMM1	SAMM50	TMEM66
AES	CD81	EP400	IDH1	MTCP1	PMPCA	SAP30	TMEM97
AFF1	CD8A	EPAS1	IDH2	MTERFD1	PMPCB	SARS	TNFAIP1
AFF4	CD8B	EPB41	IDO1	MTM1	PMS1	SART3	TNFAIP6
AFP	CDC14A	EPB41L1	IFI16	MTMR1	PNMA1	SASH3	TNFRSF1B
AGFG2	CDC16	EPB41L2	IFI27	MTMR11	PNN	SAT1	TNFRSF25
AGGF1	CDC23	EPB41L3	IFI30	MTMR2	PNOC	SATB1	TNFSF10
AGL	CDC25B	EPB41L5	IFI35	MTMR3	PNRC1	SAV1	TNFSF15
AGPAT1	CDC25C	EPHB1	IFI44	MTMR9	PNRC2	SBNO2	TNIK
AGPAT2	CDC37L1	EPHB2	IFI44L	MTOR	POGZ	SCAMP3	TNK2
AGPAT3	CDC42EP2	EPHX2	IFI6	MTR	POLA1	SCAP	TNKS
AGRN	CDC42EP4	EPM2A	IFIH1	MTRF1	POLD2	SCARA3	TNNI3
AGTPBP1	CDC45	EPM2AIP1	IFIT1	MUC1	POLD3	SCARB2	TNNT2
AHCTF1	CDC7	EPN1	IFIT2	MUS81	POLDIP2	SCARF1	TNNT3
AHNAK	CDCA3	EPN3	IFIT3	MVP	POLE3	SCCPDH	TNPO1
AHSA1	CDCA4	EPS8L1	IFIT5	MX1	POLM	SCLY	TNPO2
AIP	CDH6	ERAL1	IFITM1	MX2	POLR1C	SCMH1	TOB1
AIPL1	CDK13	ERBB2	IFT20	MXD1	POLR1E	SCO2	TOMM20
AK5	CDK14	ERCC3	IFT57	MYB	POLR2B	SDCCAG3	TOMM22
AKAP1	CDK4	ERCC5	IGHD	MYBL1	POLR2G	SDR39U1	TOMM34
AKAP11	CDK5	ERCC6L	IGHM	MYBL2	POLR2H	SEC24B	TOMM70A
AKAP13	CDKN1A	ERI3	IKBKAP	MYBPC3	POLR2I	SEC31B	TONSL
AKAP8	CDKN1B	ERP44	IKZF4	MYC	POLR3B	SEC61A1	TOP1
AKAP9	CDKN1C	ESPL1	IL10	MYD88	POLR3E	SEC61B	TOP2B
AKR1A1	CDKN2B	ESYT1	IL10RA	MYH6	POLR3F	SEC63	TOR1A
AKR1B1	CDR2	ETAA1	IL11RA	MYH9	POMP	SECTM1	TOR1B
AKR1C4	CDV3	ETV6	IL13	MYO10	PON2	SEH1L	TP53I3
AKT2	CDX1	ETV7	IL15	MYO15A	POP5	SEMA6B	TPD52
ALCAM	CDX4	EVL	IL15RA	MYO1D	POU2AF1	SENP3	TPI1
ALDH18A1	CEACAM1	EXOSC2	IL16	MYO7A	PPAP2A	SENP6	TPMT
ALDH1A2	CEACAM3	EXOSC8	IL18RAP	MYO9A	PPARD	SEPHS1	TPP1
ALDH3B1	CEACAM7	EXPH5	IL1B	MYOD1	PPAT	SEPT11	TPR
ALDH9A1	CELA3B	EXT1	IL1RL2	MYOF	PPIE	SEPT4	TPT1
ALDOC	CENPC	EXTL2	IL1RN	MZF1	PPIH	SEPT6	TRA2B
ALG13	CENPQ	EZH1	IL27RA	MZT2B	PPP1CA	SEPT9	TRAC
ALMS1	CEP192	F11R	IL2RA	N4BP1	PPP1CC	SERBP1	TRAF1

ALPI	CEP57	F2RL2	IL32	NAA50	PPP1R13B	SERP1	TRAF3IP1
AMMECR1	CEP76	FABP3	IL4R	NACA	PPP1R15A	SERPINE1	TRAF3IP3
ANAPC10	CERK	FADS2	IL7	NADK	PPP1R16B	SERPING1	TRAF5
ANAPC5	CERS2	FADS3	IL7R	NAE1	PPP1R8	SERTAD2	TRAM1
ANGPT2	CETN3	FAF1	ILF2	NAGK	PPP2R4	SESN1	TRAPPC10
ANKFY1	CETP	FAIM3	ILF3	NAP1L1	PPP2R5E	SET	TRAPPC2
ANKHD1	CFB	FAM120A	IMP3	NAP1L2	PPP3CA	SETD3	TRAPPC6A
ANKLE2	CFLAR	FAM13B	IMPDH2	NAP1L3	PPP3CB	SETD5	TRAT1
ANKRD28	CFTR	FAM162A	INADL	NAP1L4	PPP3CC	SF3A3	TRBC1
ANP32B	CGGBP1	FAM168B	INPP4B	NAPA	PPP6C	SF3B1	TREX1
ANP32C	CHCHD7	FAM178A	INPP5E	NARS	PPP6R2	SF3B5	TRIM13
ANXA2	CHD1L	FAM208B	INPP5F	NARS2	PPP6R3	SFI1	TRIM2
ANXA3	CHD3	FAM46A	INPP5J	NAT10	PPRC1	SFMBT1	TRIM21
ANXA5	CHD9	FAM64A	INSL3	NAT6	PRDM1	SFRP1	TRIM22
ANXA7	CHEK2	FAN1	INSR	NAT8B	PRDM10	SGK1	TRIM23
APBA2	CHN1	FANCA	INTS8	NBEA	PRDM2	SGSM2	TRIM25
APBB1IP	CHP2	FANCE	IPCEF1	NBN	PRDM4	SH2B1	TRIM26
APEH	CHPF	FANCF	IPO5	NCALD	PREPL	SH2D1A	TRIM28
APEX1	CHRNA6	FANCG	IPP	NCAM1	PRICKLE3	SH2D3A	TRIM32
APEX2	CIAO1	FARS2	IQCB1	NCAPD3	PRIM1	SH2D4A	TRIM38
APITD1	CIB1	FASTKD1	IRAK3	NCAPH	PRIM2	SH3BP2	TRIM5
APOA2	CIR1	FASTKD2	IRF1	NCBP1	PRKACB	SH3BP4	TRIP11
APOBEC3G	CIRBP	FAU	IRF2	NCBP2	PRKCA	SH3BP5	TRIP6
APOBR	CKAP2	FBL	IRF7	NCK1	PRKCD	SH3GLB1	TRIT1
APOL1	CKAP4	FBP2	IRF9	NCKAP1L	PRKCG	SHC3	TRMT11
APOL6	CKM	FBXO11	IRS2	NCL	PRKCH	SHMT2	TRMT112
APPL1	CLASP2	FBXO21	ISCU	NCOA4	PRKCI	SHQ1	TRMT61B
APPL2	CLCN2	FBXO3	ISG15	NCR3	PRKCQ	SIAH1	TRPM2
APRT	CLDN18	FBXO46	ISG20	NCSTN	PRKCSH	SIGIRR	TRPV4
APTX	CLEC2D	FBXW4	ITGA6	NDC1	PRKCZ	SIGLEC1	TRPV5
AQP3	CLINT1	FCER1A	ITGA7	NDRG3	PRKD3	SIK1	TRPV6
AQR	CLK2	FCER1G	ITGAE	NDST1	PRKDC	SIN3B	TRRAP
ARF3	CLOCK	FCHO1	ITGB3	NDUFA10	PRKRA	SIRPG	TSC1
ARFGEF1	CLPB	FCN1	ITGB3BP	NDUFB11	PRKRIP1	SIRT1	TSC22D4
ARFIP2	CLPP	FFAR2	ITGB4	NDUFB8	PRMT1	SIRT3	TSN
ARHGAP15	CLSTN1	FGF16	ITK	NDUFS3	PRMT2	SIT1	TSPYL1
ARHGAP26	CLTC	FGF5	ITM2C	NDUFV1	PRNP	SIVA1	TSPYL4
ARHGAP5	CMPK1	FGF9	ITPK1	NEBL	PRPF31	SIX3	TSPYL5
ARHGDIB	CMTR1	FGFR3	ITPKB	NELFA	PRPF39	SKAP1	TSSC1
ARHGEF11	CNGA1	FGL2	ITPKC	NELL2	PRPF4B	SKIV2L2	TTC19
ARHGEF17	CNIH1	FH	ITPR1	NET1	PRPF8	SKP1	TTC27
ARHGEF18	CNNM3	FHL3	ITPR3	NEU3	PRPS1	SKP2	TTC3
ARHGEF6	CNOT7	FIBP	ITSN1	NEURL	PRR16	SLAMF1	TTC37
ARHGEF7	CNP	FIG4	IVNS1ABP	NFATC2IP	PRR5L	SLAMF8	TTLL5
ARHGEF9	CNPY2	FKBP14	JAK2	NFATC3	PRSS21	SLBP	TTN

ARIH2	COCH	FKBP15	JMJD7-PLA2G4B	NFE2L1	PSEN1	SLC11A2	TUBA4A
ARL1	COG2	FKBP3	JOSD1	NFIL3	PSEN2	SLC15A3	TUBA8
ARL5A	COG5	FLT3LG	JUND	NFKBIB	PSIP1	SLC16A1	TUBB
ARMC1	COIL	FLT4	KANK1	NFKBIE	PSMB2	SLC16A3	TUBGCP3
ARMC8	COL4A3	FN3KRP	KAT6B	NFS1	PSMB4	SLC19A2	TUBGCP4
ARPC5L	COL8A2	FNBP1	KCND1	NFU1	PSMB9	SLC1A3	TUBGCP5
ARSA	COMMD3	FNBP4	KCNH6	NFX1	PSMD5	SLC22A11	TXK
ARSB	COPS7B	FNTA	KCNJ2	NFYB	PSME1	SLC22A4	TXN2
ARSD	COPS8	FOLR2	KCNJ6	NGDN	PSME2	SLC25A12	TXNRD3
ASB1	COPZ1	FOSL2	KCNMA1	NGLY1	PSPN	SLC25A17	TYMP
ASB9	COQ9	FOXJ3	KCNMB1	NHP2	PTBP2	SLC25A3	UAP1
ASF1A	CORO1B	FOXN2	KDM3A	NHP2L1	PTCD2	SLC25A32	UAP1L1
ASGR2	COX11	FOXO1	KDM3B	NIF3L1	PTCD3	SLC25A5	UBA2
ASH1L	COX4I1	FOXP3	KDM4C	NIPSNAP1	PTCRA	SLC31A1	UBA5
ASH2L	COX6C	FSTL1	KDM5B	NISCH	PTDSS1	SLC33A1	UBA7
ASIP	CPD	FTO	KHDRBS1	NIT2	PTGES2	SLC35C1	UBASH3A
ASNS	CPNE7	FTSJ1	KHK	NKIRAS2	PTGES3	SLC35D2	UBE2C
ASUN	CPSF3L	FUBP3	KIAA0226	NKTR	PTGIR	SLC35F6	UBE2D2
ASXL1	CPSF7	FURIN	KIAA1279	NKX3-2	PTK2B	SLC37A1	UBE2G1
ATAD2	CR2	FUT4	KIAA1598	NKX6-1	PTOV1	SLC38A1	UBE2G2
ATAD3A	CRBN	FXN	KIF13B	NLK	PTPLAD1	SLC39A1	UBE2I
ATF3	CRCT1	FXYD5	KIF1C	NLRP1	PTPLB	SLC39A14	UBE2L6
ATF5	CREBZF	FYB	KIF20B	NME3	PTPN11	SLC39A6	UBE3A
ATF7IP2	CRHR2	FYCO1	KIF21B	NMI	PTPN21	SLC3A2	UBE4A
ATG14	CRIM1	FYN	KIF3C	NMT2	PTPN22	SLC4A7	UBE4B
ATG3	CRLF1	FZD2	KIFAP3	NNT	PTPN7	SLC6A12	UBR5
ATG5	CRNN	FZD6	KIR3DL3	NOC3L	PTPRC	SLC6A4	UBTF
ATG7	CRTAM	G0S2	KIT	NOD1	PTPRCAP	SLC7A6	UBXN4
ATIC	CRY1	GABRD	KLF2	NOL11	PTPRK	SLC7A7	UBXN7
ATM	CRYBB3	GADD45B	KLF4	NOL3	PTPRM	SLC8A1	UCKL1
ATP13A2	CRYZ	GADD45G	KLF5	NONO	PTPRN	SLC9A3R1	UFC1
ATP1A1	CSDE1	GALE	KLF9	NOP56	PTPRU	SLC9A5	UFL1
ATP1B3	CSE1L	GALK1	KLHDC2	NOS3	PUM1	SLC9A6	UFM1
ATP5A1	CSH1	GALNT4	KLHDC3	NOSIP	PUM2	SMAD1	UFSP2
ATP5B	CSNK1D	GALT	KLHL20	NOTCH4	PURA	SMAD2	UGDH
ATP5G2	CSNK1G3	GAP43	KLHL22	NPAT	PUS1	SMARCA2	UMPS
ATP5H	CSNK2A2	GAR1	KLHL3	NPBWR2	PUS7	SMARCA4	UNC93B1
ATP6AP2	CST4	GAS6	KLRB1	NPC1	PVRL2	SMARCC1	UPB1
ATPIF1	CSTF1	GAS7	KLRG1	NPEPPS	PWP1	SMARCD3	UPF3A
ATRX	CTCF	GATA3	KLRK1	NPHP4	QARS	SMARCE1	UPF3B
ATXN10	CTDSP1	GATM	KNTC1	NPM1	QPRT	SMC2	URI1
ATXN2	CTNNA1	GBE1	KPNA1	NPM3	QRSL1	SMC4	USE1
AUH	CTNND1	GBP1	KPNB1	NPPA	QTRT1	SMC6	USO1
AVPR2	CTSE	GBP2	KRBOX4	NPRL2	RAB11FIP2	SMCR7L	USP13
AXL	CTSL	GCFC2	KRIT1	NPY4R	RAB22A	SMEK1	USP14

AZI1	CUL1	GCG	KRR1	NR2C1	RAB3D	SMPD2	USP16
AZIN1	CUL3	GCH1	KRT10	NR2E1	RAB40B	SMURF1	USP20
B3GALT2	CUL4A	GCHFR	KYNU	NR3C1	RAB4A	SMYD2	USP21
B3GNT1	CUL4B	GCN1L1	L1CAM	NR3C2	RAB6A	SMYD3	USP22
B4GALT5	CUL5	GCNT1	L2HGDH	NR4A1	RAB8B	SNCB	USP24
BACE2	CUTA	GDI2	L3MBTL1	NRF1	RABEP2	SNCG	USP25
BACH2	CUTC	GDPD2	LAGE3	NRP2	RABGEF1	SNRNP200	USP32
BAG2	CUX1	GEMIN4	LAIR1	NSD1	RABGGTB	SNRNP40	USP47
BAG3	CX3CR1	GFPT1	LANCL1	NSG1	RAD1	SNRNP70	USP48
BAG5	CXCL10	GGA1	LAP3	NSMAF	RAD17	SNRPB	USP7
BAHCC1	CXCL6	GGA2	LARP4	NSMCE4A	RAD50	SNRPF	USP9X
BAHD1	CXCR3	GGH	LARP4B	NT5E	RAE1	SNTB1	UXS1
BAI1	CXCR4	GGT1	LAT	NTSR1	RALB	SNX4	UXT
BAK1	CXCR5	GGT5	LAX1	NUCB1	RALGAPA1	SNX5	VAMP1
BANK1	CXCR6	GHSR	LBR	NUCB2	RALGPS1	SOCS1	VCPIP1
BAP1	CXorf57	GIF	LCK	NUDCD3	RALY	SOCS2	VDAC3
BATF3	CYB5A	GIMAP5	LDB1	NUDT9	RANBP2	SOCS3	VDR
BAZ2A	CYBB	GJA3	LDHB	NUFIP1	RANBP9	SOCS5	VEGFB
BBS1	CYFIP1	GLI3	LDLRAP1	NUP153	RAP2B	SOD1	VIPR1
BBS10	CYFIP2	GLMN	LDOC1	NUP155	RARA	SON	VPRBP
BBS9	CYP11B2	GLO1	LEF1	NUP160	RASGRP1	SORT1	VPREB3
BCAP31	CYP17A1	GLOD4	LEMD3	NUP210	RASGRP2	SOSTDC1	VPS13A
BCKDHB	CYP1A1	GLP1R	LETMD1	NUP43	RASSF1	SOX1	VPS13C
BCL11A	CYR61	GLS	LGALS3BP	NUP50	RBBP7	SOX17	VPS51
BCL11B	DAAM1	GLTSCR2	LGALS8	NUP54	RBCK1	SP1	VRK1
BCL2L1	DAP3	GLUL	LGALS9	NUP88	RBFA	SP100	VRK2
BCL2L10	DARS	GMEB2	LIAS	NUPL2	RBFOX1	SP110	VWA8
BCL2L14	DBF4	GMPPA	LIF	NVL	RBL2	SPAG16	WARS
BCL7B	DCAF11	GNA15	LILRB1	NXF1	RBM12	SPCS1	WAS
BCL9	DCAF8	GNB2L1	LILRB2	NXT1	RBM14	SPDL1	WBP11
BCLAF1	DCTD	GNB5	LILRB3	OAS1	RBM15	SPECC1L	WDR12
BCR	DCTPP1	GNE	LILRB4	OAS2	RBM25	SPG7	WDR3
BCS1L	DDAH2	GNG7	LIMA1	OAS3	RBM26	SPHK1	WDR48
BDH1	DDB1	GNL3L	LIME1	OASL	RBM34	SPIB	WDR59
BDH2	DDIT4	GNPAT	LIMK2	OFD1	RBM39	SPOCK2	WDR6
BEND5	DDO	GNRH1	LIN7A	OGDH	RBM4B	SPR	WDR62
BHLHE41	DDX1	GNS	LIPT1	OGFR	RBM5	SPSB1	WDR7
BIN1	DDX10	GOLGA1	LLGL2	OGT	RBM8A	SPTAN1	WDR73
BIRC3	DDX18	GOLGA3	LMBRD1	OLA1	RBMS2	SPTB	WDR77
BLK	DDX27	GOLGA4	LMNA	OPA1	RCAN3	SPTLC2	WDR83OS
BLMH	DDX28	GOLGA7	LMNB1	OPRL1	RCN2	SQRDL	WHSC1
BLVRA	DDX39B	GOLGA8A	LMO2	OR2C1	RDH8	SRBD1	WNT11
BMP8B	DDX42	GORASP1	LMX1B	OR51E2	REC8	SRC	WNT7B
BMPR1A	DDX46	GORASP2	LPCAT4	ORC2	RECK	SRGAP2	WRN
BMPR2	DDX47	GOT1	LPIN1	ORC5	RELB	SRPK2	WWP1

BMS1	DDX5	GP1BA	LPXN	ORM1	REM1	SRPRB	XAF1
BPTF	DDX50	GP9	LRBA	OSBP	RENBP	SRPX	XBP1
BRCC3	DDX56	GPATCH2	LRIG1	OSBPL10	REPIN1	SRRM1	XPA
BRD1	DDX58	GPATCH8	LRMP	OSBPL3	RERE	SRRT	XPC
BRD8	DDX60	GPD1L	LRPPRC	OSGEPL1	RET	SRSF1	YAF2
BRDT	DEDD	GPD2	LRRC1	OSM	REV1	SRSF10	YEATS4
BRIX1	DENND1A	GPER1	LRRC40	OTOF	REV3L	SRSF11	YES1
BRWD1	DENND4A	GPHN	LRRC47	OTUB1	RFC1	SRSF2	YIPF1
BSDC1	DES	GPR171	LRRC59	OXA1L	RFC3	SRSF3	YLPM1
BST1	DFFB	GPR18	LSM14A	OXCT1	RFC4	SRSF5	YTHDC1
BST2	DGKA	GPR183	LSM2	OXSM	RFC5	SRSF6	YTHDC2
BTAF1	DGKD	GPR21	LSM4	P2RX1	RFX1	SRSF7	YTHDF1
BTBD1	DHPS	GPRASP1	LSM7	P2RX7	RFX2	SRSF8	YWHAQ
BTG1	DHRS3	GPT	LTB	P2RY10	RFX7	SRSF9	YWHAZ
BTG3	DHRS9	GPX4	LTBP3	P2RY6	RGCC	SS18L1	YY1
BUB3	DHX15	GPX7	LTBR	P4HTM	RGL1	SSBP2	ZAP70
C10orf10	DHX16	GRAMD1C	LUC7L	PAAF1	RGS12	SSR2	ZBED4
C10orf137	DHX30	GRIK1	LUC7L2	PABPC3	RGS3	SSR4	ZBP1
C10orf88	DHX58	GRM4	LUC7L3	PABPC4	RGS7	SSRP1	ZBTB14
C11orf57	DHX9	GRM5	LY6E	PAFAH1B2	RHD	ST3GAL2	ZBTB18
C11orf73	DIABLO	GRN	LY6G6D	PAFAH2	RHO	ST3GAL4	ZBTB20
C12orf43	DIAPH2	GSDMB	LY9	PAICS	RHOF	ST3GAL5	ZBTB24
C14orf1	DIDO1	GSK3A	LYRM4	PALB2	RHOG	ST6GAL1	ZBTB25
C14orf166	DIEXF	GSN	LZTFL1	PAN2	RHOH	ST8SIA1	ZBTB5
C15orf39	DIMT1	GSPT2	MAF	PANK2	RIN1	STAG1	ZBTB7A
C16orf80	DIS3	GSTO1	MAFB	PANK4	RIN2	STAR	ZBTB7B
C17orf70	DISC1	GTF2A1	MAGED1	PAPD7	RINT1	STARD8	ZC3H12A
C19orf60	DKC1	GTF2A2	MAGEH1	PARK7	RIOK2	STAT1	ZC3H14
C19orf66	DKK1	GTF2F2	MAK16	PARN	RIPK1	STAT2	ZC3HAV1
C1GALT1	DKKL1	GTF2H1	MAL	PARP1	RIPK2	STAT3	ZCCHC11
C1QA	DLAT	GTF3C2	MALT1	PARP11	RLBP1	STAT4	ZCCHC14
C1QB	DLG1	GTF3C3	MAN1B1	PARP12	RMDN3	STAU2	ZCCHC2
C1QBP	DLST	GTF3C5	MAN1C1	PARP2	RNASE1	STC2	ZEB1
C2	DNAH3	GTPBP1	MAN2A1	PARP3	RNASEH1	STK25	ZFP36
C2orf44	DNAH7	GTPBP3	MAP2K4	PASK	RNASEH2B	STK38	ZHX2
C3AR1	DNAJA1	GTPBP8	MAP2K5	PATZ1	RNASEL	STK39	ZKSCAN7
C4BPA	DNAJA3	GUCA1A	MAP2K6	PAX6	RNF103	STMN1	ZMAT3
C5	DNAJB1	GUCY1A3	MAP3K14	PAX7	RNF113A	STT3A	ZMYM2
C6orf48	DNAJB12	GUCY2F	MAP3K4	PAXIP1	RNF115	STUB1	ZMYND11
C7orf26	DNAJB6	GVINP1	MAP3K6	PBX2	RNF122	STX12	ZNF10
C7orf49	DNAJB9	GYG1	MAP4K1	PBX3	RNF19B	STX16	ZNF107
C8orf33	DNAJC10	GZMA	MAP4K3	PBXIP1	RNF220	STX18	ZNF131
CA9	DNAJC16	GZMK	MAP4K5	PCBP2	RNF31	STXBP1	ZNF133
CAB39	DNAJC2	H1FX	MAPK14	PCCA	RNF38	STYXL1	ZNF135
CABIN1	DNAJC3	H2AFJ	MAPK9	PCDHGA11	RNF4	SUCLA2	ZNF14

CACNA1A	DNAL4	H2AFV	MAPKAPK5	PCID2	RNF6	SUCLG1	ZNF140
CACNA1G	DNASE2	HABP4	MARCH3	PCK2	RNGTT	SUGP2	ZNF142
CACNA1S	DNMT3L	HADH	MARCKS	PCM1	RNH1	SUN1	ZNF146
CACYBP	DOCK4	HADHA	MARCKSL1	PCMTD2	RNMT	SUN2	ZNF174
CADPS	DOK3	HAMP	MAST2	PCNP	RNMTL1	SUPT16H	ZNF184
CALCOCO2	DOLPP1	HARS	MATR3	PCNT	RNPS1	SUPT7L	ZNF202
CALD1	DONSON	HAUS3	MBD4	PCNXL4	ROM1	SUPV3L1	ZNF205
CALM1	DPH5	HBEGF	MBTPS1	PCSK6	RORA	SURF1	ZNF212
CALR	DPP4	HCFC1R1	MCCC1	PCSK7	RPA1	SURF2	ZNF217
CAMK2G	DPP8	HDAC1	MCM3	PCYT1A	RPA2	SUV420H1	ZNF232
CAMKMT	DRAP1	HDAC3	MCM4	PCYT1B	RPGRIP1	SWAP70	ZNF239
CAMLG	DRD3	HDAC9	MCM7	PDC	RPL10	SYN2	ZNF24
CAMSAP1	DROSHA	HEATR2	MCPH1	PDCD1	RPL10L	SYNE1	ZNF274
CAND1	DSC1	HECA	MDC1	PDCD11	RPL11	SYTL2	ZNF281
CANX	DSG1	HEG1	MDFIC	PDCD1LG2	RPL12	TACC1	ZNF302
CAPG	DSTYK	HEMK1	MDH2	PDCD2	RPL13	TAF11	ZNF331
CAPNS1	DUSP12	HERC1	MDK	PDCD4	RPL13A	TAF1C	ZNF337
CAPRIN2	DUSP13	HERC2	MEAF6	PDCD6	RPL14	TAF1D	ZNF354A
CAPZB	DUSP2	HERC5	MECP2	PDE4A	RPL15	TAF4	ZNF395
CARD9	DUSP22	HERC6	MED14	PDE6H	RPL18	TAF5	ZNF45
CASK	DUSP3	HERPUD1	MED22	PDE8A	RPL19	TAF9	ZNF451
CASP1	DUSP5	HEYL	MEF2C	PDGFA	RPL22	TAP1	ZNF510
CASP10	DUSP6	HFE	MEF2D	PDGFD	RPL23	TAP2	ZNF512B
CASP4	DUT	HGF	MEFV	PDIA2	RPL24	TAPBP	ZNF76
CASP5	DYNLT1	HIBCH	METAP1	PDLIM4	RPL27	TARBP1	ZNF764
CASP6	DYRK1A	HINFP	METAP2	PDS5A	RPL3	TARBP2	ZNF839
CASP7	DYRK2	HINT1	METTL17	PDS5B	RPL30	TARDBP	ZNHIT1
CASP8AP2	DYSF	HIPK3	METTL3	PDSS2	RPL35A	TASP1	ZNHIT3
CBL	DZIP3	HIST1H2AB	METTL5	PDXDC1	RPL36	TAZ	ZNHIT6
CBLB	E2F1	HIST1H2AM	MEX3C	PEBP1	RPL37	TBC1D4	ZSCAN9
CBR4	E2F5	HIST1H2BB	MFN1	PERP	RPL37A	TBCE	ZW10
CBX1	EBNA1BP2	HIST1H3D	MGA	PEX1	RPL38	TBKBP1	ZXDC
CBX7	ECE1	HIST1H4E	MGAT1	PEX11B	RPL4	TBL1XR1	ZYX
CBY1	ECHDC2	HIST1H4H	MICALL1	PEX19	RPL5	TBP	ZZZ3

Table S2 The 770 proteins selected in the dataset GSE2565

Symbol	Symbol	Symbol	Symbol	Symbol	Symbol	Symbol	Symbol
Aars	Cd3g	Eg383901	Hipk2	Loc672215	Osmr	Rfpl4	Tek
Abcc1	Cd4	Eg545124	Hk2	Loc677317	Ottmusg00000014672	Rgs11	Tex10
Abcd3	Cd48	Eg627245	Hmga1	Loc677319	Ottmusg00000015538	Rhoj	Tfpi2
Abhd14a	Cd68	Eg665937	Hmgcs1	Lox	Papolg	Rhou	Tgds
Ablim1	Cd79a	Eg666031	Hmgxb3	Lpl	Pard6b	Rin1	Tgfb1i1
Acadl	Cd9	Eg667723	Hmox1	Lrg1	Pcf11	Ripk4	Tgif1
Acadvl	Cdc45l	Egr1	Hnrnpul2	Lrp2	Pcx	Rnasek	Tgm2
Acot10	Cdca5	Egr2	Homer2	Lrrc41	Pde4b	Rnf5	Tgoln1
Acot9	Cdh2	Eid1	Hoxa4	Lrrc59	Pdgfb	Rpa1	Thbs1
Acp5	Cdh5	Eif1a	Hoxa5	Lsm4	Pdk2	Rpl22l1	Thbs2
Acta1	Cdkn1a	Eif2s2	Hp	Ltbp4	Pdk4	Rps17	Thoc4
Actn1	Cdkn1c	Eif3c	Hs1bp3	Lyve1	Pecam1	Rps27l	Timp1
Adamts4	Cds2	Eif3e	Hs6st1	Macrod1	Pfn2	Rps6ka2	Timp3
Adm	Cdt1	Eif3j	Hsbp1	Maff	Pgd	Rrm1	Tinagl1
Adss	Cdv3	Eif5	Hsd17b12	Mafk	Pgk1	Rrm2	Tiparp
Afp	Cebpd	Eif6	Hsp90aa1	Mal	Phlda1	Rrs1	Tkt
Ahcyl2	Cenpe	Ell2	Hsp90ab1	Map2k6	Phldb1	Rsad2	Tle6
Aifm1	Ces3	Eln	Hspa1a	Map3k7ip3	Phlpp	Rtkn2	Tlk2
Akr1b8	Ch25h	Elovl7	Hspa1b	March5	Phxr4	Rtn2	Tm7sf3
Akr1c13	Chi3l1	Enc1	Hspa5	March7	Pi4k2b	Rwdd2b	Tmcc2
Aldh1a1	Chic2	Ensmusg00000040078	Hspa8	Mbd1	Pim1	S100a11	Tmem119
Aldh3a1	Chmp4b	Ensmusg00000050347	Hspa9	Mboat1	Pim3	S100a14	Tmem159
Aldoa	Chordc1	Epb4.1l5	Hspb1	Mcart1	Pitpnc1	S100a6	Tmem184a
Als2cr13	Chrnb1	Epha2	Hspb8	Mcee	Pkm2	S100a8	Tmem37
Amd-ps3	Churc1	Ercc6l	Hspd1	Mcm5	Pkp2	S100a9	Tmem45a
Ampd3	Cidea	Ereg	Hspe1	Mcm7	Pkp3	Samd8	Tmem49
Angptl4	Ckb	Errfi1	Hsph1	Med8	Pla1a	Sars	Tnc
Anks1	Cks1b	Esd	Id4	Meis1	Pla2g15	Sash1	Tnfrsf12a
Anln	Cldn3	Ext2	Idi1	Melk	Pla2g4a	Sc4mol	Tnfsf12
Anxa1	Cldn4	F3	Ier2	Men1	Plaa	Scamp1	Tnpo3
Anxa3	Cldn7	F7	Ifi202b	Mest	Plaur	Scd1	Tnrc6c
Anxa4	Clec16a	Fabp4	Ifi203	Mett11d1	Plcb3	Scel	Tob2
Anxa7	Clec4a2	Fam108a	Ifrd1	Mgat5	Plekhf1	Scp2	Topbp1
Anxa8	Clec4n	Fam134b	Igj	Mif	Plk3	Sdc1	Tor1b
Aox1	Clip4	Fam69b	Igsf5	Mmp11	Plod2	Sdc3	Tpi1
Apoc2	Clu	Fanca	Il1b	Mmp15	Plp2	Sdccag3	Tpm2
Arc	Clybl	Fasn	Il1r1	Mmp3	Plscr2	Sec61a1	Trak1
Arcn1	Col3a1	Fbln2	Il1r2	Mprip	Pmaip1	Sec62	Trdmt1
Areg	Col6a3	Fbxo4	Il33	Mpzl2	Pno1	Selm	Trf
Arf3	Cops3	Fcgrt	Impact	Mrpl16	Pnrc1	Sema3f	Trib3
Arg1	Coq7	Fdps	Incenp	Mrpl28	Podxl	Sephs2	Trim35
Arg2	Cotl1	Fgfbp1	Inmt	Mrpl30	Pomp	Sept2	Trip13
Arhgap21	Cpne1	Fgfr4	Ints6	Mrpl46	Pon3	Serpina1a	Trp53inp1

Arhgef12	Creg1	Fgfrl1	Ipo4	Mrpl51	Ppa1	Serpina1b	Tsc22d1
Arid1a	Crygn	Fggy	Ipo5	Mrps12	Ppan	Serpina3n	Tsc22d3
Arid5a	Csf1r	Fgl1	Ipo7	Ms4a6d	Ppap2b	Serpinb9b	Tspyl4
Armcx4	Csf3r	Fkbp1b	Irs2	Ms4a8a	Pparg	Serpine1	Ttc39c
Arvcf	Ctla2a	Fkbp5	Itgax	Msln	Ppl	Serpine2	Tuba4a
Asns	Ctps	Flt3l	Itgb5	Msr1	Ppp2r2a	Sfxn1	Tubb2c
Atf3	Ctsh	Fmnl3	Itih4	Msrb2	Pprc1	Siah2	Tuft1
Atf4	Ctsk	Fmod	Itm2b	Mt1	Prdx1	Sin3b	Tusc3
Atl2	Ctsz	Fos	Jam2	Mt2	Prdx6	Skiv2l	Txnl1
Atp11a	Cuta	Fosl1	Jun	Mtch1	Prelp	Slc16a1	Txnrd1
Atp1a2	Cxcl16	Foxj1	Junb	Muc1	Prkcb	Slc16a7	U2af1
Atp6ap2	Cxcl17	Foxq1	Kcnb1	Mybbp1a	Prkci	Slc1a1	Ubap1
Atp6v1a	Cxcr7	Fst	Kcnk1	Myc	Prkd2	Slc20a1	Ubc
Atxn10	Cyp2a4	Fxc1	Kctd9	Myct1	Procr	Slc25a13	Ube2c
Azi2	Cyp4b1	Gadd45a	Kif22	Myd116	Prr13	Slc25a29	Ube2f
B3gnt4	Cyp51	Gadd45g	Kif23	Myo5a	Prss22	Slc25a5	Ube2k
Bach1	Cyp7b1	Gars	Kif2c	Nampt	Psat1	Slc2a1	Ubqln1
Bach2	Cyr61	Gca	Kit	Nars	Psd3	Slc31a1	Ubr3
Bag3	Cyth3	Gch1	Klf4	Nbeal2	Psma4	Slc38a4	Ubxn4
Basp1	Dab2ip	Gclc	Klf5	Nbr1	Psma5	Slc3a2	Uchl1
Bcat2	Dbi	Gclm	Klf6	Nck1	Psma7	Slco3a1	Uchl3
Bcl10	Dbnl	Gdpd2	Klhdc8a	Ncl	Psmb4	Smo	Uchl5
Bex2	Dcbld1	Ghitm	Klhl2	Ndrg1	Psmb5	Snhg1	Uhrf1
Blvrb	Dctn5	Ghr	Kras	Nedd4l	Psmb6	Snhg6	Uhrf1bp1l
Bmp6	Dctpp1	Gja1	Krt18	Nfkbia	Psmc1	Snx5	Unc84a
Bnip3	Ddi2	Gjb3	Krt19	Ngfrap1	Psmc2	Socs3	Unc84b
Bola2	Ddit3	Gla	Krt7	Nhp2	Psmc6	Sorcs2	Uqcrfs1
Brd2	Ddit4	Glg1	Krt8	Nipbl	Psmd1	Sorl1	Usp7
Btg2	Ddx39	Glul	Kti12	Nkain4	Psmd14	Spcs3	Vamp5
Bub1b	Ddx50	Gnaz	Lace1	Nkg7	Psmd3	Spnb2	Vcam1
C1r	Dedd2	Gnl3	Lama3	Nmd3	Psmd5	Spp1	Vcp
C330027c09rik	Dgat2	Gp49a	Lamc2	Nme2	Psmd6	Sqle	Vwa5a
C4b	Dhx32	Gprc5c	Lcn2	Nme7	Ptbp1	Sqrdl	Wbp5
C630004h02rik	Dmkn	Gpx2	Ldha	Notch2	Ptges	Sqstm1	Wsb1
Cacnb2	Dnaja1	Grb10	Ldlr	Nov	Ptgr1	Sri	Xbp1
Cacybp	Dnaja4	Gsr	Lgals1	Npm1	Ptgs1	Srm	Xpot
Carkd	Dnajb1	Gsta1	Lgals3	Nqo1	Ptgs2	Srxn1	Ypel3
Cbfa2t3	Dnajb4	Gsta2	Lgals3bp	Nr1d1	Ptms	Ssna1	Yrdc
Cbr3	Dnajb9	Gsta3	Lif	Nr4a1	Ptp4a1	Sssca1	Ythdf2
Ccdc117	Dnajc2	Gsta4	Litaf	Nsmce1	Ptp4a3	Stat3	Zbtb16
Ccdc88c	Dnajc21	Gtf2f2	Loc100046232	Nsun4	Ptplb	Stat5b	Zc3h18
Ccl12	Dnajc5	Gtf3c1	Loc100046344	Nup210	Ptpn14	Stk40	Zeb1
Ccl2	Dnpep	Gtlf3b	Loc100046567	Nup50	Ptpn21	Stt3a	Zeb2
Ccl6	Dpy30	Gusb	Loc100047601	Nup54	Pttg1	Sult1a1	Zfand2a
Ccng2	Dtl	H2afx	Loc100047840	Nupl1	Qsox1	Surf4	Zfp110

Ccnl1	Dusp1	Has2	Loc100047868	Nupr1	Rab31	Tacc3	Zfp207
Ccr1	Dusp16	Hbegf	Loc100047896	Obfc2a	Rabl4	Taldo1	Zfp281
Ccr2	E2f6	Hdac5	Loc100048076	Ogfrl1	Rad23b	Tapbp	Zfp292
Ccr5	Ebi3	Helz	Loc14210	Ogn	Ranbp1	Taz	Zfp36
Ccrn4l	Edem3	Hes6	Loc630729	Omd	Rarg	Tbc1d15	Zfp574
Cct3	Edn1	Hexb	Loc635418	Orf61	Rars	Tbca	Zfp90
Cct6a	Eef2k	Higd2a	Loc638798	Orm1	Rdh10	Tbx2	Zkscan1
Cd2ap	Efemp2	Hint1	Loc640441	Osgin1	Rfk	Tc2n	Zwint
Cd300a	Eg103324						

Table S3 The 755 proteins selected in the dataset GSE13268

Symbol	Symbol	Symbol	Symbol	Symbol	Symbol	Symbol	Symbol
Aanat	Cald1	Dnm1	Gss	LOC679958	Parm1	RGD1307682	Sppl2a
Abca3	Calr	Dnm2	Gsta3	LOC681004	Pax8	RGD1308059	Spry2
Abhd3	Caly	Dnm3	Gsta5	LOC682360	PCOLCE2	RGD1308117	Sptbn1
Acbd4	Cant1	Dpep1	Gstm2	LOC682469	Pdc	RGD1308448	Sra1
Acly	Capza1	Dpysl4	Gstm5	LOC682999	Pde1b	RGD1308772	Srpx
Acot2	Car5b	Dusp3	Gstm7	LOC685277	Pde3b	RGD1308782	Ssfa2
Acox1	Card9	Dync1h1	Gsto1	LOC685707	Pde6d	RGD1309350	Stk32c
Acpl2	Cars	Ebf1	Gsto2	LOC686774	Pdgfrl	RGD1309534	Stmn4
Acsf2	Cast	Ednrb	Gstp1	LOC687090	Pdhb	RGD1309540	Strn
Acsl4	Cbx3	Egfr	Gstt1	LOC688228	Pdhx	RGD1309651	Sts
Acsl5	Ccdc127	Egr1	Gstt2	LOC689147	Pdia3	RGD1309676	Sulf2
Acss2	Ccdc17	Ehd1	Gtf2i	LOC689329	Pdia6	RGD1310348	Sult1a1
Actr1a	Ccl24	Ehd2	Guk1	LOC691170	Pdlim2	RGD1310351	Svep1
Actr1b	Ccl9	Eif3d	Gys2	Loxl2	Pdzrn3	RGD1310587	Synj1
Adamts15	Ccna2	Eif5a2	Hbp1	Ltv1	Pfkfb3	RGD1559909	Synj2
Adcy4	Ccnb2	Eif5b	Hcca2	Lyplal1	Pgcp	RGD1560011	Tac1
Adfp	Ccnd2	Emilin2	Hcrtr2	Lztr1	Pgm5	RGD1562284	Tada1l
Adhfe1	Cd163	Enah	Hes3	Man1b1	Pgrmc1	RGD1562717	Tbata
Aga	Cd209g	Entpd4	Hist2h2aa3	Map2k1	Pias3	RGD1563422	Tbc1d9b
Agap2	Cd55	Epb4.1l2	Hist3h2a	Map2k2	Picalm	RGD1563684	Tbl1x
Agpat4	Cd5l	Epc2	Hmgcs2	Map4k4	Pigq	RGD1563863	Tcf25
Ahcyl2	Cd74	Ephx2	Hnrph1	Map7d1	Pik3r3	RGD1564228	Tcf7l2
Ahnak	Cd9	Erh	Homer2	Mbd3	Pim3	RGD1564541	Tect2
Aig1	Cd99	Ermp1	Hprt1	Mdfic	Pip5k1b	RGD1565002	Tes
Ak3	Cdc20	Esrra	Hpx	Me1	Pir	RGD1565432	Tex15
Ak3l1	Cdc5l	Etnk2	Hs2st1	Mecr	Pitpnb	Rgs7bp	Tgfb1i1
Akap12	Cdh13	Etv1	Hsd11b2	Med4	Pja2	Rhbdf1	Tgfbr3
Akr1b7	Cdk105	Fabp5	Hsd17b12	Meis2	Plbd1	Rmnd5b	Tgm2
Akt1	Cdr2	Fabp6	Hsd17b8	Meox2	Plcb1	Rraga	Tgoln1
Akt2	Cela1	Fah	Hsf2	Mettl6	Plce1	Rragc	Tinagl1
Aldh1a1	Cenpk	Faim	Hspa12a	Mfn2	Pld1	Rrm2	Tk1
Aldh1a2	Cep70	Fam103a1	Hspa4l	MGC109340	Pld2	Scand1	Tkt
Alg11	Ces3	Fam105a	Hspe1	MGC72955	Plekhg3	Scara5	Tmem120a
Anapc11	Cetn2	Fam107a	Ica1	MGC72974	Plin	Scd1	Tmem150c
Ang1	Cfp	Fam117b	Idh1	Mgll	Plp2	Scn7a	Tmem160
Angptl4	Cgref1	Fam171a1	Ifrd2	Mgmt	Plxna2	Scpep1	Tmem195
Ankrd52	Chpt1	Fat1	Ift20	Mgst2	Pmepa1	Sdf2	Tmem206
Ankrd6	Chrdl1	Fbln2	Igfbp3	Mipep	Pmm1	Sec62	Tmem208
Ano10	Chrna1	Fbn1	Igfbp6	Mki67	Pnpla3	Sel1l	Tmem43
Ano6	Cirbp	Fbxo23	Igsf1	Mmp2	Pola2	Selenbp1	Tmem45b
Anxa1	Clcn4-2	Fbxo4	Il13ra2	Mospd1	Pold2	Selm	Tmem50b
Anxa5	Clec10a	Fcrls	Il17re	Mpzl2	Polr1c	Senp6	Tmsbl1
Anxa6	Clec7a	Fdx1l	Il18bp	Mrp63	Pols	Sept8	Tnfaip1

Ap2a2	Cltb	Fgf11	Il34	Mrpl21	Ppap2c	Serf1	Tp53i13
Ap2s1	Cma1	Fgfr1	Ipmk	Mrpl32	Ppapdc1b	Serpina12	Trdmt1
Ap3m2	Cmc1	Fgl2	Iscu	Mrpl37	Ppie	Serpinb9	Trhde
Apex1	Cmtm6	Fhl1	Isoc1	Msh2	Ppm1k	Serpinf1	Trip10
Apln	Cndp1	Fitm2	Itgam	Mt1a	Ppp1cc	Serpinh1	Tshr
Apln	Cnih4	Fkbp11	Itgb2	Mterf	Ppp2r2b	Setd3	Tslp
Aplp1	Cnksr2	Fkbp1a	Itgb5	Mtmr10	Ppp4r2	Sft2d2	Tsnax
Aplp2	Cnot1	Fmo1	Kcnd3	Mxd4	Pqlc1	Sfxn1	Tspan12
Appl2	Cnr1	Fmo2	Kcnip1	Mxra8	Prdx3	Sgms1	Tspan9
Arhgdib	Col15a1	Fmo3	Kcns3	Myeov2	Prdx4	Sh2d4a	Ttc1
Arhgef15	Col4a1	Fn1	Kctd10	N6amt2	Prl6a1	Sh3bgrl	Ttc25
Arhgef18	Col4a2	Fndc1	Kctd9	Nanos3	Prom1	Sh3bgrl3	Ttc27
Armc9	Col5a2	Folr1	Kifap3	Nck2	Prpsap2	Sh3tc1	Tubb3
Armcx1	Col5a3	Foxn3	Kifc1	Ndufb3	Prss23	Shmt1	Tubb5
Asf1a	Col8a1	Frmd5	Klf15	Ndufc1	Psma4	Siglec5	Tusc4
Asgr2	Copb2	Frs2	Klf9	Neo1	Psma6	Slain2	Tusc5
Asna1	Cp	Frzb	Klhdc5	Nfia	Psme2	Slc12a4	Twsg1
Atf5	Cpeb1	Fstl1	Lamb2	Nfkb1	Pter	Slc17a5	Uap1l1
Atl2	Cpne8	Ftsj3	Lamp1	Nfkbia	Ptprm	Slc20a1	Uchl1
Atp2b3	Crebl2	Fxyd5	Larp5	Nfyc	Ptprr	Slc25a1	Ulk2
Atp4a	Crebzf	Fzd1	Lcmt1	Nit1	Ptprz1	Slc25a36	Unc93b1
Atp4b	Crim1	G6pd	Ldhb	Nkain3	Purg	Slc2a3	Usf2
Atp6v0c	Crlf2	Gbe1	Lef1	Nkd1	Pwwp2a	Slc31a2	Usp39
Atpif1	Crot	Gbp2	Lgals3	Nkx3-1	Pxdn	Slc35a1	Utp15
Atrn	Csf2ra	Gcgr	Lgr4	Nlk	Pxk	Slc38a10	Utp3
Atxn2	Csgalnact1	Gdap2	Lhx5	Nme1	Pygl	Slc38a7	Uxs1
Atxn2l	Ctbp2	Geft	Lipa	Nnmt	Rab13	Slc39a8	Vamp1
Avpr2	Cthrc1	Gfpt2	Litaf	Npdc1	Rab22a	Slc41a2	Vkorc1l1
B3gntl1	Ctnnbip1	Gif	Lix1l	Npr3	Rab4a	Slc43a1	Vma21
B4galt5	Ctnnbl1	Gja1	Lmf2	Nptn	Rab7l1	Slc43a2	Vps29
B4galt6	Ctps2	Gja7	LOC100360120	Npy1r	Rap1gap	Slc9a3	Wasl
Bace2	Ctsa	Glb1l2	LOC100360205	Nr4a1	Rapgef4	Slco2b1	Wbp2
Basp1	Ctsb	Glrx1	LOC100361444	Nr4a2	Rarres2	Slco3a1	Wbp5
Basp1	Ctsh	Glt8d1	LOC100362324	Nr4a3	Rasal2	Slmap	Wdfy2
Bat2d1	Ctsz	Gnai1	LOC100362346	Nsg1	Rassf4	Smarca1	Wdr18
Bckdhb	Cttn	Gnb1	LOC100363005	Nudt7	Rbm3	Smarce1	Wdr37
Bcl2l13	Cul1	Gnb2	LOC100363014	Nup37	Rbms3	Smoc2	Xbp1
Blcap	Cyp11a1	Gnb3	LOC100363743	Nxt1	Rbmx	Smpd2	Xdh
Blnk	Cyp11b1	Gnb5	LOC100366017	Ocrl	Rbp7	Smpd3	Ykt6
Bloc1s1	Cyp4f5	Gng11	LOC100366259	Odf3b	Rcan2	Snx8	Zadh2
Bnip2	Dab1	Golga3	LOC24906	Olah	Rcn3	Snx9	Zbtb24
Brd7	Dapk1	Golsyn	LOC296884	Olfml3	Rdh12	Soat1	Zdhhc20
Bre	Dbil5	Gpam	LOC304903	Olr1	Recql	Sorbs2	Zdhhc4
Brms1l	Dcakd	Gpc4	LOC305633	Omd	Reep5	Sox17	Zfp180
Brp44l	Ddc	Gpld1	LOC306766	Orc5l	Rem1	Sp1	Zfp189

Brsk1	Ddr2	Gpr116	LOC363060	Osbpl5	Rft1	Sparc	Zfp398
Btnl1	Ddt	Gprasp1	LOC363331	p18	RGD1304595	Spire1	Zfp423
Bub3	Ddx19a	Gpsm2	LOC494529	P4ha1	RGD1307218	Spire2	Zfp426l
Bysl	Dgkz	Gpx7	LOC500046	Pank1	RGD1307220	Spns2	Zfp469
C1galt1c1	Dlk1	Grb10	LOC501091	Papss2	RGD1307315	Spop	Zh16
C6	Dnajc12	Grb14	LOC502894	Parg	RGD1307396	Spp1	Zwilch
Cabin1	Dnajc30	Gria3					

Table S4 Basic information of dynamic networks for the influenza H3N2 data (GSE30550)

GSE30550	number of nodes		number of edges	
	asymptomatic	symptomatic	asymptomatic	symptomatic
**initial**	2400	2400	16498	16498
**0h**	2093	2120	8519	9143
**5h**	2031	2139	7829	8932
**12h**	2017	2095	7166	8173
**21h**	2063	2132	7065	8749
**29h**	2066	2096	7769	8504
**36h**	1985	2161	6993	9325
**45h**	1965	2147	7169	10017
**53h**	2079	2183	7680	10498
**60h**	2048	2150	7908	10363
**69h**	2049	2047	6855	7995
**77h**	2062	2107	7693	8743
**84h**	2084	2080	7974	8661
**93h**	2011	2087	7320	8507
**101h**	1965	2042	7073	8264
**108h**	2114	2152	7688	8445

Table S5 Basic information of dynamic networks for the influenza H1N1 data (GSE52428)

GSE52428	number of nodes		number of edges	
	asymptomatic	symptomatic	asymptomatic	symptomatic
**initial**	2400	2400	16498	16498
**0h**	2037	2060	7911	8541
**5h**	1963	2019	6845	8113
**12h**	1967	2004	6874	7967
**21h**	1943	2025	7050	8436
**29h**	2004	2041	7562	8687
**36h**	1967	2094	7259	9677
**45h**	1991	2138	7297	10453
**53h**	1924	2172	6951	11409
**60h**	1960	2191	7388	11404
**69h**	2087	2083	8400	10119
**77h**	2005	2176	7778	11138
**84h**	2064	2082	7872	9877
**93h**	2013	2035	7918	9289
**101h**	1926	2022	6911	8474
**108h**	2023	2117	7250	8829

Table S6 Basic information of dynamic networks for the acute lung injury data (GSE2565)

GSE2565	number of nodes		number of edges	
	control	case	control	case
**initial**	770	770	5890	5890
**0h**	640	640	4586	4624
**0.5h**	640	638	4553	4586
**1h**	641	637	4504	4561
**8h**	638	639	4496	4521
**12h**	638	639	4489	4502
**24h**	640	636	4463	4492
**48h**	634	636	4427	4443
**72h**	637	639	4409	4433

Table S7 Basic information of dynamic networks for the type 2 diabetes mellitus data (GSE13268)

GSE13268	number of nodes		number of edges	
	control	case	control	case
**initial**	755	755	4054	4054
**4w**	624	623	2684	2799
**8w**	619	621	2407	2505
**12w**	624	625	2272	2366
**16w**	621	623	2124	2268
**20w**	620	624	2038	2176

Table S8 Protein modules appearing in each time point in the asymptomatic networks for the influenza H3N2 data (GSE30550)

	the proteins in module							
	DDX58	HERC5	HERC6	IFI27	IFI35	IFI44	IFI44L	IFI6
**module 1**	IFIH1	IFIT1	IFIT2	IFIT3	IRF7	IRF9	ISG15	MX1
	OAS1	OAS2	OAS3	OASL	RSAD2	XAF1	…	
**module 2**	CD19	CD22	CD72	CD79A	CD79B	IGHM	MS4A1	POU2AF1
**module 3**	PDS5B	RNGTT	PDS5A	STAG1				
**module 4**	DIS3	EXOSC2	EXOSC8	GTPBP1	SKIV2L2	ZFP36	SUPV3L1	
**module 5**	C14orf166	DDX1	RTCB					
**module 6**	TIMM10	TIMM22	TIMM9	TOMM20	TOMM70A			

Table S9 Protein modules appearing in each time point in the symptomatic networks for the influenza H3N2 data (GSE30550)

	the proteins in module							
module 1	ADAR	DDX58	DDX60	GBP1	GBP2	HERC6	IFI27	IFI35
	IFI44	IFI44L	IFI6	IFIH1	IFIT1	IFIT2	IFIT3	IFIT5
	IFITM1	IRF2	IRF7	IRF9	ISG15	ISG20	MX1	MX2
	OAS1	OAS2	OAS3	OASL	RSAD2	RTP4	XAF1 …	
module 2	CD19	CD22	CD72	CD79A	CD79B	IGHM	MS4A1	POU2AF1
module 3	PDS5B	RNGTT	PDS5A	STAG1				
module 4	CDX4	NAP1L1	NAP1L2	NAP1L3	NAP1L4			
module 5	AZI1	CEP192	ALMS1	CEP57	CEP76	OFD1	PCNT	SFI1
	TUBGCP3	TUBGCP4	TUBGCP5					

Table S10 Protein modules appearing in each time point in the asymptomatic networks for the influenza H1N1 data (GSE52428)

	the proteins in module							
module 1	ADAR	DDX58	GBP1	GBP2	HERC6	IFI27	IFI35	IFI44
	IFI44L	IFI6	IFIH1	IFIT1	IFIT2	IFIT3	IFITM1	IRF1
	IRF2	IRF7	IRF9	ISG15	ISG20	MX1	MX2	OAS1
	OAS2	OAS3	OASL	RSAD2	RTP4	XAF1	…	
module 2	PDS5B	PDS5A	RNGTT	STAG1				

Table S11 Protein modules appeared in each time point in symptomatic networks for influenza H1N1 data (GSE52428)

	the proteins in module							
module 1	GBP1	GBP2	ADAR	IFI27	IFI35	IFI44	IFI44L	IFI6
	IFIH1	IFIT1	IFIT2	IFIT3	IFITM1	IRF1	IRF2	IRF7
	IRF9	ISG15	ISG20	MX1	MX2	OAS1	OAS2	OAS3
	OASL	RSAD2	RTP4	XAF1	…			
module 2	CDX4	NAP1L1	NAP1L2	NAP1L3	NAP1L4			

Table S12 Protein modules appearing in each time point in the asymptomatic networks for the acute lung injury data (GSE2565)

	the proteins in module							
module 1	Bnip3	Hk2	Aldoa	Ldha	Pgd	Pgk1	Pkm2	Slc2a1
	Taldo1	Tkt	Tpi1	Arhgef12	Pard6b	Prkci	Rhoj	Rhou
	Gtf2f2	Pcf11	Papolg	Ptbp1	U2af1			
module 2	Akr1b8	Aldh3a1	Gclc	Gclm	Gpx2	Gsr	Gsta1	Gsta2
	Gsta3	Gsta4	Loc100047868	Mafk	Nqo1	Sult1a1	Txnrd1	
module 3	Dnajc2	Dnajc21	Eif1a	Gnl3	Kti12	Nmd3	Nsun4	Rrs1
module 4	Cldn3	Cldn4	Cldn7	Kcnk1	Krt18	Krt19	Krt7	Krt8

Table S13 Protein modules appearing in each time point in the case networks for the acute lung injury data (GSE2565)

	the proteins in module							
	Bnip3	Hk2	Aldoa	Ldha	Pgd	Pgk1	Pkm2	Slc2a1
**module 1**	Taldo1	Tkt	Tpi1	Arhgef12	Pard6b	Prkci	Rhoj	Rhou
	Gtf2f2	Pcf11	Papolg	Ptbp1	U2af1			
**module 2**	Ensmusg00000050347	Lgals1	Lgals3	Plp2	S100a11	Anxa8	S100a6	
**module 3**	Akr1b8	Aldh3a1	Gclc	Gclm	Gpx2	Gsr	Gsta1	Gsta2
	Gsta3	Gsta4	Loc100047868	Mafk	Nqo1	Sult1a1	Txnrd1	
**module 4**	Actn1	Acta1	Spnb2	Tnc	Tpm2			

Table S14 Protein modules appearing in each time point in the control networks for the type 2 diabetes mellitus data (GSE13268)

	the proteins in module							
	Akr1b7	Fmo1	Glrx1	Gpx7	Gss	Gsta3	Gstm2	Gstm5
	Gstm7	Gsto1	Gsto2	Gstp1	Gstt1	Gstt2	Me1	Mgst2
**module 1**	Prdx3	Sult1a1	Xdh	Ankrd6	Frzb	Fzd1	Acpl2	Lef1
	Nkd1	Nlk	Sox17	C6	Ermp1	Ppap2c	Sgms1	Smpd2
	Smpd3	Cyp11a1	Cyp11b1	Fdx11	Hsd11b2	Tmem50b		
**module 2**	Adcy4	Anxa1	Ccl9	Cnr1	Ednrb	Gcgr	Geft	Gnai1
	Gnb2	Gnb3	Gng11	Hcrtr2	Npy1r	Pik3r3	Plcb1	Tac1
**module 3**	Acox1	Acot2	Acsf2	Acsl4	Acsl5	Adfp	Angptl4	Fabp5
	Lyplal1	Mgll	Plin	Pnpla3	Scd1			

Table S15 Protein modules appearing in each time point in the case networks for the type 2 diabetes mellitus data (GSE13268)

	the proteins in module							
	Akr1b7	Fmo1	Glrx1	Gpx7	Gss	Gsta3	Gstm2	Gstm5
	Gstm7	Gsto1	Gsto2	Gstp1	Gstt1	Gstt2	Me1	Mgst2
**module 1**	Prdx3	Sult1a1	Xdh	Ankrd6	Frzb	Fzd1	Acpl2	Lef1
	Nkd1	Nlk	Sox17	C6	Ermp1	Ppap2c	Sgms1	Smpd2
	Smpd3	Cyp11a1	Cyp11b1	Fdx11	Hsd11b2	Tmem50b		
**module 2**	Adcy4	Anxa1	Ccl9	Cnr1	Ednrb	Gcgr	Geft	Gnai1
	Gnb2	Gnb3	Gng11	Hcrtr2	Npy1r	Pik3r3	Plcb1	Tac1
**module 3**	Agpat4	Dgkz	Chpt1	Etnk2	Gpam	Gpld1	Pld1	Pld2
	Ppap2c							
	Apex1	Ctnnbl1	Ddx19a	Erh	Ftsj3	Hspe1	Ltv1	Mrpl21
**module 4**	Mrpl32	Mt1a	Nup37	Nxt1	Polr1c	Psma4	Psma6	Ttc27
	Utp15	Utp3	Wdr18					
**module 5**	Adamts15	Cela1	Fmo2	Pmm1	Serpina12			

Table S16 All protein modules appearing in each time point in both the asymptomatic and symptomatic networks for the influenza H3N2 data (GSE30550)

	the proteins in module							
**module 1 (HM1)**	DDX58	HERC5	HERC6	IFI27	IFI35	IFI44	IFI44L	IFI6
	IFIH1	IFIT1	IFIT2	IFIT3	IRF7	IRF9	ISG15	MX1
	OAS1	OAS2	OAS3	OASL	RSAD2	XAF1	…	
**module 2**	CD19	CD22	CD72	CD79A	CD79B	IGHM	MS4A1	POU2AF1
**module 3**	PDS5B	RNGTT	PDS5A	STAG1				

Table S17 All protein modules appearing in each time point in both the asymptomatic and symptomatic networks for the influenza H1N1 data (GSE52428)

	the proteins in module							
**module 1 (HM2)**	ADAR	DDX58	GBP1	GBP2	HERC6	IFI27	IFI35	IFI44
	IFI44L	IFI6	IFIH1	IFIT1	IFIT2	IFIT3	IFITM1	IRF1
	IRF2	IRF7	IRF9	ISG15	ISG20	MX1	MX2	OAS1
	OAS2	OAS3	OASL	RSAD2	RTP4	XAF1		

Table S18 All protein modules appearing in each time point in both the asymptomatic and symptomatic networks for the acute lung injury data (GSE2565)

	the proteins in module							
**module 1 (HM3)**	Bnip3	Hk2	Aldoa	Ldha	Pgd	Pgk1	Pkm2	Slc2a1
	Taldo1	Tkt	Tpi1	Arhgef12	Pard6b	Prkci	Rhoj	Rhou
	Gtf2f2	Pcf11	Papolg	Ptbp1	U2af1			
**module 2**	Akr1b8	Aldh3a1	Gclc	Gclm	Gpx2	Gsr	Gsta1	Gsta2
	Gsta3	Gsta4	Loc100047868	Mafk	Nqo1	Sult1a1	Txnrd1	

Table S19 All protein modules appearing in each time point in both the asymptomatic and symptomatic networks for the type 2 diabetes mellitus data (GSE13268)

	the proteins in module							
**module 1 (HM4)**	Akr1b7	Fmo1	Glrx1	Gpx7	Gss	Gsta3	Gstm2	Gstm5
	Gstm7	Gsto1	Gsto2	Gstp1	Gstt1	Gstt2	Me1	Mgst2
	Prdx3	Sult1a1	Xdh	Ankrd6	Frzb	Fzd1	Acpl2	Lef1
	Nkd1	Nlk	Sox17	C6	Ermp1	Ppap2c	Sgms1	Smpd2
	Smpd3	Cyp11a1	Cyp11b1	Fdx1l	Hsd11b2	Tmem50b		
**module 2**	Adcy4	Anxa1	Ccl9	Cnr1	Ednrb	Gcgr	Geft	Gnai1
	Gnb2	Gnb3	Gng11	Hcrtr2	Npy1r	Pik3r3	Plcb1	Tac1

Table S20 Descriptions of the HM1 of the influenza H3N2 data (GSE30550)

Symbol	Descriptions
IFIH1	interferon induced with helicase C domain 1
HERC6	hect domain and RLD 6
OAS3	2'-5'-oligoadenylate synthetase 3, 100kDa
HERC5	hect domain and RLD 5
RSAD2	radical S-adenosyl methionine domain containing 2
IFI44L	interferon-induced protein 44-like
IFI44	interferon-induced protein 44
OAS1	2',5'-oligoadenylate synthetase 1, 40/46kDa
OAS2	2'-5'-oligoadenylate synthetase 2, 69/71kDa
IFI35	interferon-induced protein 35
DDX58	DEAD (Asp-Glu-Ala-Asp) box polypeptide 58
IFIT3	interferon-induced protein with tetratricopeptide repeats 3
IRF9	interferon regulatory factor 9
IFIT2	interferon-induced protein with tetratricopeptide repeats 2
IFIT1	interferon-induced protein with tetratricopeptide repeats 1
IFI27	interferon, alpha-inducible protein 27
OASL	2'-5'-oligoadenylate synthetase-like
ISG15	ISG15 ubiquitin-like modifier
IRF7	interferon regulatory factor 7
XAF1	XIAP associated factor 1
MX1	myxovirus (influenza virus) resistance 1, interferon-inducible protein p78 (mouse)
IFI6	interferon, alpha-inducible protein 6

Table S21 Descriptions of the HM2 of the influenza H1N1 data (GSE52428)

Symbol	Descriptions
IFIH1	interferon induced with helicase C domain 1
RTP4	receptor (chemosensory) transporter protein 4
IFITM1	interferon induced transmembrane protein 1 (9-27)
OAS3	2'-5'-oligoadenylate synthetase 3, 100kDa
RSAD2	radical S-adenosyl methionine domain containing 2
IFI44L	interferon-induced protein 44-like
OAS1	2',5'-oligoadenylate synthetase 1, 40/46kDa
OAS2	2'-5'-oligoadenylate synthetase 2, 69/71kDa
IFI35	interferon-induced protein 35
ISG20	interferon stimulated exonuclease gene 20kDa
ISG15	ISG15 ubiquitin-like modifier
XAF1	XIAP associated factor 1
MX1	myxovirus (influenza virus) resistance 1, interferon-inducible protein p78 (mouse)
MX2	myxovirus (influenza virus) resistance 2 (mouse)
HERC6	hect domain and RLD 6
IFI44	interferon-induced protein 44
DDX58	DEAD (Asp-Glu-Ala-Asp) box polypeptide 58
IRF9	interferon regulatory factor 9
IFIT3	interferon-induced protein with tetratricopeptide repeats 3
IFIT2	interferon-induced protein with tetratricopeptide repeats 2
OASL	2'-5'-oligoadenylate synthetase-like
IFI27	interferon, alpha-inducible protein 27
IFIT1	interferon-induced protein with tetratricopeptide repeats 1
IRF7	interferon regulatory factor 7
IRF1	interferon regulatory factor 1
IRF2	interferon regulatory factor 2
IFI6	interferon, alpha-inducible protein 6
GBP2	guanylate binding protein 2, interferon-inducible
ADAR	adenosine deaminase, RNA-specific
GBP1	guanylate binding protein 1, interferon-inducible, 67kDa

Table S22 Descriptions of the HM3 of the acute lung injury data (GSE2565)

Symbol	Descriptions
Rhoj	ras homolog gene family, member J
Pkm2	predicted gene 6560; predicted gene 2124; predicted gene 6992; pyruvate kinase,muscle;similar to M2-type pyruvate kinase
U2af1	predicted gene 10232; predicted gene 6978; U2 small nuclear ribonucleoprotein auxiliary factor (U2AF) 1
Ptbp1	polypyrimidine tract binding protein 1; predicted gene 4900
Pgk1	phosphoglycerate kinase 1; predicted gene, EG668435
Rhou	ras homolog gene family, member U
Arhgef12	predicted gene 7281; predicted gene 5831; similar to SP140 nuclear body exchange factor (GEF) 12 protein (predicted);Rhoguanine nucle otide
Tkt	transketolase
Tpi1	triosephosphate isomerase 1; similar to triosephosphate isomerase 1
Gtf2f2	general transcription factor IIF, polypeptide 2
Pard6b	par-6 (partitioning defective 6) homolog beta (C. elegans)
Pcf11	cleavage and polyadenylation factor subunit homolog (S. cerevisiae)
Aldoa	aldolase A, fructose-bisphosphate; predicted gene 8767; predicted gene 7556
Taldo1	transaldolase 1
Prkci	protein kinase C, iota
Ldha	predicted gene 7997; lactate dehydrogenase A; predicted gene 5452
Bnip3	predicted gene 14506; BCL2/adenovirus E1B interacting protein 3; predicted gene 6532; similar to E1B 19K/B cl-2-binding protein homolog
Slc2a1	solute carrier family 2 (facilitated glucose transporter), member 1
Papolg	poly(A) polymerase gamma
Hk2	hexokinase 2
Pgd	phosphogluconate dehydrogenase

Table S23 Descriptions of the HM4 of the type 2 diabetes mellitus data (GSE13268)

Symbol	Descriptions
Fmo1	flavin containing monooxygenase 1
C6	complement component 6
Fdx1l	ferredoxin 1-like
Xdh	xanthine dehydrogenase
Gss	glutathione synthetase
Gstp1	glutathione-S-transferase, pi 1
Tmem50b	transmembrane protein 50B
Sgms1	sphingomyelin synthase 1
Gsta3	glutathione S-transferase A3
Nkd1	naked cuticle homolog 1 (Drosophila)
Mgst2	microsomal glutathione S-transferase 2
Sox17	SRY (sex determining region Y)-box 17
Gpx7	glutathione peroxidase 7
Cyp11b1	cytochrome P450, subfamily 11B, polypeptide 1
Gstt1	glutathione S-transferase theta 1
Gstt2	glutathione S-transferase, theta 2
Prdx3	peroxiredoxin 3
Lef1	lymphoid enhancer binding factor 1
Nlk	nemo like kinase
Frzb	frizzled-related protein
Gstm2	glutathione S-transferase mu 2
Ankrd6	ankyrin repeat domain 6
Ppap2c	phosphatidic acid phosphatase type 2c
Smpd3	sphingomyelin phosphodiesterase 3, neutral
Smpd2	sphingomyelin phosphodiesterase 2, neutral
Me1	malic enzyme 1, NADP(+)-dependent, cytosolic
Fzd1	frizzled homolog 1 (Drosophila)
Akr1b7	aldo-keto reductase family 1, member B7
Gsto2	glutathione S-transferase omega 2
Gsto1	glutathione S-transferase omega 1
Acpl2	acid phosphatase-like 2
Ermp1	endoplasmic reticulum metallopeptidase 1
Hsd11b2	hydroxysteroid 11-beta dehydrogenase 2
Cyp11a1	cytochrome P450, family 11, subfamily a, polypeptide 1
Glrx1	glutaredoxin 1 (thioltransferase)
Sult1a1	sulfotransferase family, cytosolic, 1A, phenol-preferring, member 1
Gstm5	glutathione S-transferase, mu 5
Gstm7	glutathione S-transferase mu 3; glutathione S-transferase mu 4; glutathione S-transferase, mu 7

Table S24 Functional enrichment of GO biological processes in the identified DNB of the influenza H3N2 data (GSE30550)

Genes in DNB	GO Term	Description	P-Value
{DDX58,IRF9,IFIH1,ISG15,IRF7,RSAD2,IFI44,MX1,IFI35}	GO:0009615	response to virus	2.23E-13
{DDX58,IRF9,IFIH1,ISG15,IRF7,RSAD2,IFI44,MX1,IFI35}	GO:0051707	response to other organism	5.97E-10
{DDX58,IRF9,IFIH1,ISG15,IRF7,RSAD2,IFI44,MX1,IFI35}	GO:0009607	response to biotic stimulus	5.47E-09
{DDX58,IFIH1,OASL,OAS3,RSAD2,IFI44L,OAS1,OAS2,IFI6,IFI35}	GO:0006955	immune response	2.58E-08
{DDX58,IRF9,IFIH1,ISG15,IRF7,RSAD2,IFI44,MX1,IFI35}	GO:0051704	multi-organism process	4.65E-07
{DDX58,IFIH1,OASL,OAS3,RSAD2,IFI44L,OAS1,OAS2,IFI6,IFI35}	GO:0002376	immune system process	6.17E-07
{IFIH1,OAS3,RSAD2,IFI44L,IFI44,OAS1,OAS2,IFI35,DDX58,IRF9,OASL,ISG15,IRF7,MX1,IFI6}	GO:0050896	response to stimulus	1.02E-06

Table S25 Functional enrichment of GO biological processes in the identified DNB of the influenza H1N1 data (GSE52428)

Genes in DNB	GO Term	Description	P-Value
{DDX58,IRF9,IFIH1,ISG15,IRF7,RSAD2,IFI44,MX1,MX2,IFI35,ISG20}	GO:0009615	response to virus	1.45E-15
{DDX58,IRF9,IFIH1,IFITM1,ISG15,IRF7,RSAD2,IFI44, MX1,MX2,IFI35,ISG20}	GO:0009607	response to bioticstimulus	1.66E-11
{DDX58,IRF9,IFIH1,ISG15,IRF7,RSAD2,IFI44,MX1,MX2,IFI35,ISG20}	GO:0051707	response to other organism	2.86E-11
{DDX58,IFIH1,OASL,OAS3,RSAD2,IFI44L,OAS1,OAS2, GBP2,IFI6,IFI35,GBP1}	GO:0006955	immune response	8.37E-09
{IFIH1,RTP4,IFITM1,OAS3,RSAD2,IFI44L,IFI44,OAS1, OAS2,IFI35,ISG20,DDX58,IRF9,OASL,ISG15,IRF7,MX1,MX2,IFI6,GBP2,GBP1}	GO:0050896	response to stimulus	1.04E-08
{IFIH1,OAS3,RSAD2,IFI44L,OAS1,OAS2,IFI35,DDX58, OASL,IRF1,GBP2,IFI6, GBP1}	GO:0002376	immune system process	3.21E-08
{DDX58,IRF9,IFIH1,ISG15,IRF7,RSAD2,IFI44,MX1,MX2,IFI35,ISG20}	GO:0051704	multi-organism process	1.08E-07

Table S26 Functional enrichment of GO biological processes in the identified DNB of the acute lung injury data (GSE2565)

Genes in DNB	GO Term	Description	P-Value
{ALDOA,TPI1,LDHA,TALDO1,PKM2,PGD,HK2,TKT,PGK1}	GO:0006007	glucose catabolic process	6.14E-15
{ALDOA,TPI1,LDHA,TALDO1,PKM2,PGD,HK2,TKT,PGK1}	GO:0046365	monosaccharide catabolic process	8.22E-15
{ALDOA,TPI1,LDHA,TALDO1,PKM2,PGD,HK2,TKT,PGK1}	GO:0019320	hexose catabolic process	8.22E-15
{ALDOA,TPI1,LDHA,TALDO1,PKM2,PGD,HK2,TKT,PGK1}	GO:0046164	alcohol catabolic process	4.41E-14
{ALDOA,TPI1,LDHA,TALDO1,PKM2,PGD,HK2,TKT,PGK1}	GO:0044275	cellular carbohydrate catabolic process	4.41E-14
{ALDOA,TPI1,LDHA,TALDO1,PKM2,PGD,HK2,TKT,PGK1}	GO:0016052	carbohydrate catabolic process	5.70E-13
{ALDOA,TPI1,LDHA,TALDO1,PKM2,PGD,HK2,TKT,PGK1}	GO:0006006	glucose metabolic process	6.64E-11
{ALDOA,TPI1,LDHA,TALDO1,PKM2,PGD,HK2,TKT,PGK1}	GO:0019318	hexose metabolic process	2.08E-10
{ALDOA,TPI1,LDHA,TALDO1,PKM2,PGD,HK2,TKT,PGK1}	GO:0005996	monosaccharide metabolic process	5.64E-10
{ALDOA,TPI1,LDHA,PKM2,HK2,PGK1}	GO:0006096	glycolysis	4.69E-09
{TPI1,LDHA,TALDO1,PGD,TKT}	GO:0046496	nicotinamide nucleotide metabolic process	3.36E-07
{TPI1,LDHA,TALDO1,PGD,TKT}	GO:0006769	nicotinamide metabolic process	3.71E-07
{TPI1,LDHA,TALDO1,PGD,TKT}	GO:0019362	pyridine nucleotide metabolic process	4.09E-07
{TPI1,LDHA,TALDO1,PGD,TKT}	GO:0009820	alkaloid metabolic process	4.09E-07
{TPI1,TALDO1,PGD,TKT}	GO:0006098	pentose-phosphate shunt	4.54E-07
{TPI1,LDHA,TALDO1,PGD,TKT}	GO:0006733	oxidoreduction coenzyme metabolic process	8.90E-07
{TPI1,TALDO1,PGD,TKT}	GO:0006739	NADP metabolic process	1.25E-06
{TPI1,LDHA,TALDO1,PGD,TKT}	GO:0043603	cellular amide metabolic process	1.29E-06
{TPI1,LDHA,TALDO1,PGD,TKT}	GO:0019748	secondary metabolic process	8.47E-06
{TPI1,PGD,TKT,PGK1}	GO:0046364	monosaccharide biosynthetic process	1.76E-05
{ALDOA,TPI1,LDHA,PKM2,HK2,PGK1}	GO:0006091	generation of precursor metabolites and energy	1.97E-05
{TPI1,LDHA,PKM2,PGK1}	GO:0006090	pyruvate metabolic process	3.53E-05
{TPI1,PGD,TKT,PGK1}	GO:0046165	alcohol biosynthetic process	3.53E-05
{TPI1,LDHA,TALDO1,PGD,TKT}	GO:0006732	coenzyme metabolic process	7.82E-05
{TPI1,PGD,TKT,PGK1}	GO:0034637	cellular carbohydrate biosynthetic process	1.14E-04
{TALDO1,PGD,TKT}	GO:0019321	pentose metabolic process	1.88E-04
{TPI1,LDHA,TALDO1,PGD,TKT}	GO:0051186	cofactor metabolic process	1.93E-04
{TPI1,PGD,TKT,PGK1}	GO:0016051	carbohydrate biosynthetic process	3.63E-04

Table S27 Functional enrichment of GO biological processes in the identified DNB of the type 2 diabetes mellitus data (GSE13268)

Genes in DNB	GO Term	Description	P-Value
{GSTM2,GSTA3,SULT1A1,GSTP1,GSTM7}	GO:0006805	xenobiotic metabolic process	2.63E-07
{GSTM2,GSTA3,SULT1A1,GSTP1,GSTM7}	GO:0009410	response to xenobiotic stimulus	4.32E-07
{GSS,GSTA3,GSTT1,GSTT2,GSTP1}	GO:0006749	glutathione metabolic process	5.82E-07
{ME1,XDH,ERMP1,CYP11B1,C6,SGMS1,PRDX3,GSTM5,GLRX1, GSTM7,GSS,GSTM2,FMO1,SULT1A1,GSTO2,GSTO1,GSTA3, CYP11A1,NLK,LEF1,GSTT1,GSTT2,AKR1B7,HSD11B2,SMPD3, GSTP1,SMPD2}	GO:0008152	metabolic process	2.47E-06
{GSS,GSTA3,SULT1A1,GSTT1,GSTT2,GSTP1}	GO:0006790	sulfur metabolic process	5.40E-06
{GSS,GSTA3,GSTT1,GSTT2,GSTP1}	GO:0006518	peptide metabolic process	5.91E-06
{GSS,GSTA3,SULT1A1,GSTT1,GSTT2,GSTP1}	GO:0006575	cellular amino acid derivative metabolic process	4.21E-05
{ME1,XDH,AKR1B7,CYP11A1,FMO1,CYP11B1,HSD11B2,PRDX3,GL RX1}	GO:0055114	oxidation reduction	6.44E-05
{ME1,GSTM2,CYP11A1,SULT1A1,HSD11B2,LEF1,GSTT1,PRDX3,GS TM7, MGST2}	GO:0010033	response to organic substance	2.05E-04
{CYP11A1,CYP11B1,SULT1A1,HSD11B2}	GO:0034754	cellular hormone metabolic process	4.32E-04
{CYP11A1,CYP11B1,SULT1A1,HSD11B2,SMPD3}	GO:0010817	regulation of hormone levels	4.84E-04
{GSS,GSTA3,GSTT1,GSTT2,GSTP1}	GO:0006732	coenzyme metabolic process	5.20E-04

Table S28 Pearson correlation coefficient (PCC) between the expression values of S1PR1 and those proteins of interest in the innate immunity pathways in 9 symptomatic subjects infected by the H3N2 influenza virus strain

	Symptomatic (Sx) subjects								
	1	5	6	7	8	10	12	13	15
**MDA5**	-0.378	-0.786	-0.545	-0.735	-0.79	-0.686	-0.535	-0.523	-0.643
MAPK	-0.101	0.112	0.019	-0.024	-0.077	0.59	0.481	0.705	0.318
**HERC6**	0.015	-0.806	-0.622	-0.766	-0.746	-0.648	-0.377	-0.558	-0.534
**HERC5**	-0.344	-0.819	-0.607	-0.781	-0.852	-0.701	-0.646	-0.654	-0.618
**RIG-I**	-0.43	-0.807	-0.508	-0.721	-0.788	-0.727	-0.657	-0.569	-0.629
**IFI44L**	-0.045	-0.794	-0.619	-0.749	-0.837	-0.671	-0.442	-0.61	-0.561
**OAS2**	-0.121	-0.805	-0.719	-0.806	-0.809	-0.675	-0.563	-0.66	-0.606
**RSAD2**	-0.186	-0.801	-0.603	-0.772	-0.824	-0.706	-0.556	-0.646	-0.665
**IRF7**	-0.207	-0.844	-0.734	-0.781	-0.803	-0.689	-0.489	-0.653	-0.522
**IFIT3**	-0.271	-0.839	-0.616	-0.785	-0.865	-0.719	-0.576	-0.653	-0.626
**OAS1**	-0.112	-0.787	-0.653	-0.795	-0.824	-0.678	-0.522	-0.617	-0.579
**OAS3**	-0.222	-0.816	-0.732	-0.782	-0.817	-0.694	-0.544	-0.673	-0.58
**IFI35**	-0.38	-0.843	-0.772	-0.787	-0.763	-0.707	-0.665	-0.657	-0.592
**IFI6**	-0.133	-0.789	-0.835	-0.798	-0.782	-0.69	-0.63	-0.678	-0.566
**IFI44**	-0.084	-0.767	-0.627	-0.697	-0.803	-0.666	-0.463	-0.547	-0.574
**MX1**	-0.296	-0.831	-0.745	-0.772	-0.841	-0.696	-0.582	-0.7	-0.604
**IFIT1**	-0.264	-0.803	-0.558	-0.764	-0.839	-0.694	-0.599	-0.64	-0.684
**XAF1**	-0.127	-0.715	-0.559	-0.745	-0.812	-0.697	-0.45	-0.467	-0.528
TLR3	0.465	-0.433	0.094	-0.052	-0.352	-0.371	-0.526	-0.261	-0.427
TBK1	-0.322	-0.162	-0.35	-0.104	-0.594	-0.452	0.142	0.116	-0.022
**ISG15**	-0.084	-0.801	-0.658	-0.776	-0.795	-0.713	-0.451	-0.655	-0.634
IKKβ	0.763	0.662	0.187	0.709	0.751	0.663	0.943	0.939	0.836
**IFIT2**	-0.541	-0.838	-0.516	-0.768	-0.809	-0.724	-0.696	-0.605	-0.66
MyD88	-0.159	-0.841	-0.593	-0.743	-0.798	-0.7	-0.371	-0.35	-0.289
**OASL**	-0.271	-0.85	-0.628	-0.823	-0.842	-0.722	-0.623	-0.695	-0.665
IRF3	0.413	0.071	0.026	0.448	0.698	0.699	0.315	0.669	0.837
**IRF9**	-0.329	-0.84	-0.495	-0.765	-0.866	-0.664	-0.194	-0.603	-0.417
TNF-α	0.425	-0.646	-0.58	-0.492	-0.793	-0.218	-0.101	-0.202	0.125
IL6	-0.086	-0.494	-0.357	-0.231	0.089	0.192	-0.601	0.123	-0.469
IKKα	0.162	0.342	0.313	0.16	-0.452	-0.165	0.602	0.736	0.389
NFκB	0.339	-0.202	-0.326	0.347	-0.2	0.166	0.66	0.257	0.702
**IFI27**	0.566	-0.646	-0.388	-0.617	-0.662	-0.395	-0.125	-0.372	-0.283
IFN-γ	0.209	-0.216	0.15	0.234	0.02	-0.543	-0.64	-0.473	-0.185
IFN-α	-0.03	-0.512	-0.212	-0.777	0.22	-0.175	0.01	-0.56	-0.572
IKKε	0.85	-0.316	-0.231	0.179	0.196	0.29	-0.286	-0.029	0.377
IFN-β	0.075	-0.427	-0.239	-0.033	-0.135	-0.23	-0.697	-0.734	-0.345

Note: The identified DNBs are marked in red and bold. Proteins with significant differences of correlations between the symptomatic and asymptomatic subjects are labeled in green background. The results indicate that most of the DNBs exhibit significant differences in the distributions of correlations between the two different clinical outcomes (marked in red and green background).

Table S29 Pearson correlation coefficient (PCC) between the expression values of S1PR1 and those proteins of interest in the innate immunity pathways in 8 asymptomatic subjects infected by the H3N2 influenza virus strain

	Asymptomatic (Asx) subjects								
	2	3	4	9	11	14	16	17	**P-value**
**MDA5**	-0.215	0.611	0.134	0.616	0.71	0.245	0.444	-0.204	0.000082
MAPK	-0.431	-0.508	-0.609	-0.666	-0.513	-0.417	-0.724	-0.455	0.000082
**HERC6**	-0.181	0.27	0.104	0.741	0.745	0.48	0.482	0.328	0.000165
**HERC5**	-0.407	-0.032	-0.202	0.407	0.12	-0.105	-0.047	-0.531	0.000329
**RIG-I**	-0.513	0.363	0.253	0.43	0.534	0.001	-0.009	-0.453	0.000576
**IFI44L**	-0.44	0.37	0.017	0.485	0.444	0.237	0.124	-0.524	0.000576
**OAS2**	-0.513	-0.342	-0.399	0.113	0.44	-0.012	0.039	0.091	0.000576
**RSAD2**	-0.507	0.306	-0.223	-0.103	0.237	0.158	0.032	-0.439	0.000576
**IRF7**	-0.433	-0.216	-0.159	0.46	-0.234	0.318	0.128	-0.244	0.000987
**IFIT3**	-0.51	-0.33	-0.195	0.146	-0.281	-0.032	-0.316	-0.525	0.001563
**OAS1**	-0.54	-0.175	-0.255	0.771	0.345	-0.03	0.197	-0.38	0.001563
**OAS3**	-0.576	-0.359	-0.387	-0.001	0.042	-0.009	-0.017	-0.353	0.001563
**IFI35**	-0.58	-0.36	-0.372	0.444	-0.674	0.104	-0.069	-0.425	0.002468
**IFI6**	-0.494	0.212	-0.202	0.544	-0.284	0.051	-0.025	-0.659	0.002468
**IFI44**	-0.46	0.647	0.031	0.573	0.616	-0.105	0.237	-0.616	0.002468
**MX1**	-0.519	-0.61	-0.412	-0.006	-0.196	-0.218	-0.159	-0.327	0.002468
**IFIT1**	-0.487	0.371	-0.027	0.16	-0.038	-0.283	0.227	-0.705	0.005512
**XAF1**	-0.62	-0.193	-0.373	-0.03	0.618	0.028	0.121	-0.259	0.005512
TLR3	-0.032	0.424	0.204	0.446	0.415	0.427	0.423	0.747	0.005512
TBK1	-0.067	0.564	0.629	0.262	0.436	0.179	0.565	-0.321	0.005512
**ISG15**	-0.41	0.518	-0.095	0.514	-0.121	-0.152	0.141	-0.668	0.007898
IKKβ	0.02	0.006	0.086	0.153	0.866	0.312	0.066	0.357	0.007898
**IFIT2**	-0.721	-0.38	-0.567	-0.414	-0.598	-0.451	-0.328	-0.555	0.01522
MyD88	-0.207	-0.383	-0.387	0.048	-0.065	0.187	-0.61	-0.277	0.046401
**OASL**	-0.67	-0.717	-0.448	0.137	-0.696	-0.308	-0.165	-0.432	0.074455
IRF3	0.591	-0.348	0.006	0.253	-0.16	0.387	0.192	0.556	0.074455
**IRF9**	-0.525	-0.522	-0.257	0.376	-0.54	-0.176	-0.496	-0.357	0.113945
TNF-α	-0.426	-0.12	0.406	-0.206	-0.066	-0.007	0.428	-0.191	0.138791
IL6	0.082	0.101	0.569	-0.028	0.044	-0.097	-0.311	0.311	0.138791
IKKα	0.224	0.66	0.858	0.685	0.584	0.512	0.446	-0.159	0.138791
NFκB	0.119	-0.336	-0.438	-0.005	0.512	0.004	-0.102	0.104	0.199589
**IFI27**	0.148	-0.364	-0.098	-0.442	-0.59	-0.245	-0.196	-0.155	0.23587
IFN-γ	0.404	0.314	-0.106	0.351	-0.487	-0.782	0.367	-0.067	0.276594
IFN-α	0.013	-0.727	0.228	-0.222	-0.516	-0.312	0.078	0.215	0.423447
IKKε	0.803	0.201	0.05	0.123	0.28	-0.127	0.218	0.668	0.423447
IFN-β	0.038	-0.306	-0.352	-0.368	-0.627	0.023	-0.033	0.021	0.480708

Note: The identified DNBs are marked in red and bold. Proteins with significant differences of correlations between the symptomatic and asymptomatic subjects are labeled in green background. The results indicate that most of the DNBs exhibit significant differences in the distributions of correlations between the two different clinical outcomes (marked in red and green background, P-value < 0.05).

Table S30 Pearson correlation coefficient (PCC) between the expression values of S1PR1 and those proteins of interest in the innate immunity pathways in 12 symptomatic subjects infected by the H1N1 influenza virus strain

	Symptomatic (Sx) subjects											
	2	3	6	7	8	9	10	12	13	17	19	20
**IRF7**	-0.778	-0.338	-0.727	-0.787	-0.656	-0.44	-0.5	-0.779	-0.788	-0.555	-0.906	-0.833
**RIG-I**	-0.599	0.6	-0.667	-0.874	-0.567	-0.371	0.116	-0.863	-0.848	-0.429	-0.931	-0.837
TLR3	-0.342	0.21	-0.718	-0.028	-0.242	0.07	0.227	-0.409	-0.484	0.012	-0.318	-0.359
**MDA5**	-0.401	0.825	-0.661	-0.856	-0.55	-0.328	0.713	-0.831	-0.809	-0.392	-0.939	-0.809
**IFI44**	-0.501	0.21	-0.686	-0.607	-0.558	-0.386	0.111	-0.624	-0.719	-0.405	-0.884	-0.825
**IFIT1**	-0.524	0.267	-0.699	-0.779	-0.626	-0.384	0.082	-0.759	-0.72	-0.279	-0.869	-0.803
**IFI44L**	-0.668	0.137	-0.682	-0.608	-0.578	-0.376	0.221	-0.574	-0.701	-0.444	-0.875	-0.826
**IFIT3**	-0.686	0.049	-0.712	-0.762	-0.611	-0.373	-0.228	-0.71	-0.748	-0.445	-0.902	-0.824
**RSAD2**	-0.577	0.044	-0.699	-0.663	-0.566	-0.386	0.007	-0.663	-0.743	-0.421	-0.896	-0.828
**IFI35**	-0.769	-0.446	-0.769	-0.854	-0.669	-0.416	-0.532	-0.822	-0.859	-0.557	-0.933	-0.882
**HERC5**	-0.621	0.185	-0.703	-0.829	-0.6	-0.371	0.237	-0.813	-0.786	-0.429	-0.909	-0.827
**MX1**	-0.72	-0.013	-0.706	-0.784	-0.615	-0.395	-0.141	-0.716	-0.777	-0.453	-0.892	-0.819
**HERC6**	-0.683	0.452	-0.714	-0.788	-0.595	-0.418	0.679	-0.745	-0.767	-0.475	-0.919	-0.827
**IFI6**	-0.695	0.145	-0.699	-0.675	-0.571	-0.345	-0.296	-0.538	-0.79	-0.475	-0.895	-0.821
IKKβ	0.271	-0.079	-0.113	0.464	0.091	0.252	0.389	0.952	0.645	0.203	0.764	0.736
IFN-β	-0.309	-0.44	-0.409	-0.545	-0.265	0.107	-0.088	-0.438	0	-0.047	-0.108	-0.378
**OAS1**	-0.686	0.271	-0.691	-0.679	-0.573	-0.358	0.316	-0.692	-0.762	-0.456	-0.893	-0.837
**OASL**	-0.727	-0.183	-0.723	-0.842	-0.659	-0.411	-0.41	-0.798	-0.817	-0.469	-0.926	-0.854
**ISG15**	-0.726	-0.086	-0.716	-0.695	-0.599	-0.353	-0.059	-0.602	-0.75	-0.422	-0.894	-0.83
TNF-α	-0.385	-0.404	-0.505	-0.05	-0.104	-0.043	0.014	-0.815	-0.275	-0.515	-0.801	-0.552
**OAS2**	-0.687	0.265	-0.697	-0.708	-0.61	-0.411	0.463	-0.711	-0.823	-0.495	-0.904	-0.834
TBK1	0.348	0.336	-0.168	-0.518	-0.131	-0.112	0.363	-0.85	-0.684	0.045	-0.794	-0.582
**IFIT2**	-0.62	0.03	-0.719	-0.826	-0.632	-0.431	-0.262	-0.853	-0.849	-0.399	-0.902	-0.816
**OAS3**	-0.663	0.109	-0.694	-0.742	-0.601	-0.434	0.493	-0.689	-0.791	-0.471	-0.907	-0.836
**IRF9**	-0.789	-0.123	-0.693	-0.706	-0.682	-0.418	-0.367	-0.641	-0.794	-0.367	-0.82	-0.815
MAPK	0.071	-0.342	0.074	0.581	-0.094	0.273	-0.469	-0.462	-0.136	0.128	0.247	0.631
IRF3	-0.307	-0.716	0	0.555	0.163	0.366	-0.023	0.818	0.507	-0.047	-0.031	0.355
MyD88	-0.581	0.564	-0.783	-0.816	-0.583	-0.347	0.031	-0.901	-0.831	-0.593	-0.903	-0.798
**XAF1**	-0.627	0.075	-0.669	-0.702	-0.572	-0.363	0.399	-0.616	-0.72	-0.508	-0.906	-0.826
IKKα	0.439	0.857	0.645	0.432	0.373	0.059	0.126	-0.552	0.183	0.429	0.327	-0.09
NFκB	-0.352	-0.137	-0.383	-0.045	-0.257	0.312	0.477	-0.129	0.205	-0.246	0.334	0.461
IL6	-0.446	0.182	0.103	0.153	0.039	0.692	0.133	-0.29	-0.101	0.286	0.031	-0.434
IFN-γ	0.414	-0.144	-0.256	0.098	0.149	0.34	0.122	-0.551	0.047	0.449	0.211	-0.108
**IFI27**	0.495	-0.718	-0.463	0.211	-0.45	-0.338	-0.245	-0.162	-0.529	-0.241	-0.513	-0.738
IFN-α	-0.164	-0.003	-0.328	-0.053	-0.45	0.001	-0.194	-0.618	-0.206	-0.134	-0.228	-0.002
IKKε	-0.384	-0.146	-0.352	-0.061	-0.085	0.429	0.088	0.561	0.324	-0.393	-0.046	0.136

Note: The identified DNBs are marked in red and bold. Proteins with significant differences of correlations between the symptomatic and asymptomatic subjects are labeled in green background. The results indicate that most of the DNBs exhibit significant differences in the distributions of correlations between the two different clinical outcomes (marked in red and green background).

Table S31 Pearson correlation coefficient (PCC) between the expression values of S1PR1 and those proteins of interest in the innate immunity pathways in 11 asymptomatic subjects infected by the H1N1 influenza virus strain.

	Asymptomatic (Asx) subjects											
	1	4	5	11	14	15	16	18	21	22	23	**P-value**
**IRF7**	-0.451	-0.297	-0.35	-0.356	-0.331	-0.787	-0.232	-0.617	-0.323	-0.304	-0.232	0.003
**RIG-I**	0.689	0.169	-0.264	0.467	0.313	-0.821	-0.067	-0.565	0.103	-0.22	0.8	0.007
TLR3	-0.209	0.152	0.186	0.469	0.085	-0.147	0.019	0.093	0.098	0.441	0.288	0.009
**MDA5**	0.838	0.503	-0.271	0.599	0.456	-0.765	-0.292	-0.362	0.243	-0.064	0.91	0.011
**IFI44**	0.298	0.133	-0.211	-0.205	0.308	-0.688	-0.283	-0.391	-0.009	0.037	-0.104	0.013
**IFIT1**	0.426	0.252	-0.297	0.086	0.251	-0.711	-0.142	-0.394	-0.276	-0.232	-0.107	0.013
**IFI44L**	0.11	0.173	-0.311	-0.124	0.603	-0.695	-0.011	-0.511	0.327	-0.16	-0.067	0.015
**IFIT3**	0.117	0.159	-0.354	-0.648	0.088	-0.785	-0.116	-0.509	-0.356	-0.17	-0.043	0.015
**RSAD2**	0.052	-0.02	-0.266	-0.466	0.325	-0.749	-0.145	-0.496	0.249	-0.12	0.454	0.015
**IFI35**	-0.53	-0.323	-0.402	-0.686	-0.656	-0.839	-0.261	-0.641	-0.247	-0.616	0.279	0.018
**HERC5**	0.232	0.14	-0.337	-0.258	0.454	-0.777	-0.195	-0.542	-0.182	-0.485	-0.093	0.021
**MX1**	0.067	-0.049	-0.393	-0.573	0.126	-0.771	-0.328	-0.676	-0.172	-0.595	0.104	0.021
**HERC6**	0.581	0.015	-0.258	0.228	-0.452	-0.783	0.179	-0.473	-0.024	-0.299	0.479	0.025
**IFI6**	0.155	0.116	-0.437	-0.347	-0.501	-0.769	-0.279	-0.414	-0.094	-0.285	-0.445	0.025
IKKβ	0.44	0.258	-0.474	-0.155	-0.007	0.816	-0.587	-0.263	-0.188	-0.185	0.229	0.025
IFN-β	-0.131	0.179	0.446	0.036	-0.378	0.12	-0.074	0.246	-0.257	0.136	-0.392	0.029
**OAS1**	0.588	0.469	-0.221	-0.316	-0.045	-0.724	-0.489	-0.599	-0.202	-0.411	0.261	0.034
**OASL**	-0.306	-0.083	-0.417	-0.544	-0.139	-0.819	-0.276	-0.679	-0.606	-0.556	0.156	0.034
**ISG15**	-0.462	-0.136	-0.324	-0.402	-0.457	-0.765	0.157	-0.574	0.466	-0.414	0.202	0.039
TNF-α	-0.278	0.554	0.021	0.467	-0.109	-0.587	-0.005	-0.146	0.459	0.11	-0.519	0.045
**OAS2**	0.381	-0.129	-0.233	-0.543	0.259	-0.749	-0.379	-0.698	-0.262	-0.495	0.51	0.053
TBK1	0.208	0.394	-0.101	0.669	0.524	-0.438	-0.311	0.129	-0.034	0.318	-0.087	0.06
**IFIT2**	0.052	-0.09	-0.303	-0.827	0.237	-0.833	-0.631	-0.693	-0.273	-0.217	-0.029	0.069
**OAS3**	0.228	-0.16	-0.307	-0.573	0.224	-0.794	-0.566	-0.673	-0.431	-0.448	0.171	0.069
**IRF9**	-0.315	-0.251	-0.599	-0.664	-0.059	-0.759	-0.361	-0.665	-0.444	-0.718	0.083	0.079
MAPK	0.007	-0.378	-0.231	-0.791	0.084	-0.212	-0.661	-0.565	-0.438	-0.08	0.343	0.079
IRF3	0.006	-0.41	-0.284	-0.729	-0.633	0.64	0.318	-0.17	0.001	-0.671	-0.224	0.103
MyD88	0.258	0.007	-0.42	-0.381	-0.28	-0.889	-0.696	-0.679	-0.409	-0.296	0.238	0.103
**XAF1**	0.083	0.286	-0.187	-0.816	0.269	-0.733	-0.67	-0.66	0.109	0.123	0.607	0.148
IKKα	0.759	0.549	0.313	0.775	0.542	0.164	-0.004	0.444	0.192	0.567	0.605	0.186
NFκB	-0.12	-0.213	-0.242	-0.646	-0.065	-0.103	-0.303	-0.558	-0.123	-0.529	0.503	0.23
IL6	0.145	0.091	0.267	0.01	0.107	0.431	0.417	0.244	0.413	-0.602	-0.33	0.406
IFN-γ	-0.113	0.091	0.259	-0.159	-0.256	-0.009	-0.202	0.467	-0.343	0.322	0.068	0.479
**IFI27**	-0.007	-0.342	-0.377	-0.943	-0.401	-0.571	0.298	-0.302	0.012	0.148	-0.182	0.518
IFN-α	-0.306	-0.753	0.099	-0.365	0.015	0.137	-0.044	-0.054	0.036	-0.3	-0.548	0.644
IKKε	-0.383	-0.015	0.222	0.114	-0.303	-0.536	0.48	-0.221	-0.02	-0.556	0.358	0.735

Note: The identified DNBs are marked in red and bold. Proteins with significant differences of correlations between the symptomatic and asymptomatic subjects are labeled in green background. The results indicate that most of the DNBs exhibit significant differences in the distributions of correlations between the two different clinical outcomes (marked in red and green background, P-value < 0.05).

Supplementary MATLAB source codes

****

Remark: We list two important M files for constructing the dynamic networks. The detailed MATLAB source codes are available at: http://maths.whu.edu.cn/teach/lj/?title=xfzou, or, http://maths.whu.edu.cn/d/file/shizililiang/lj/2015-01-26/a35859758c8cc953516fb2edfe73f732.pdf. If the readers are interested in all the source data and codes in this paper, they can contact with Prof. Xiufen Zou: xfzou@whu.edu.cn. We can provide all the executable M files.

****

Function LP_OP.m

```
% Function LP_OP.m: the optimization problem is transformed into a standard linear programming
% (LP) problem.
function [J,f1]=LP_OP(Y,X,lamda1,lamda2,beta)
%Y is the expression value of target gene
%X is the expression values of genes interact with target gene
%lamda1 is the regularization parameter for guarantee the sparseness
%lamda2 is the regularization parameter for guarantee the continuity
%beta is a priori information about interactions
%J is the strength of interaction relations
%f1 is the optimization of objective function values
[n,m]=size(Y);%Y is a row vector, n=1,m is the number of samples
[p,q]=size(X);%X is a matrix, p is the number of genes, q is the number of samples
c=n*m;
h=n*p;
f=[ones(1,2*c),lamda1*ones(1,2*h),lamda2*ones(1,2*h)]';
%f is a column vector, the first 2c lines are u, v coefficient, after 2h lines
%are ksai, yeta coefficient
A1=sparse(1:c,1:c,ones(1,c),c,c);
A2=sparse(1:c,1:c,-ones(1,c),c,c);
Z=X';%a line is a sample, a column is a gene
Z_1=1/2*Z;
Y_1=Y';%Y_1 is a column vector
beq=Y_1-Z_1*beta;
beq=[beq;beta];
Z_2=-Z_1;
Aeq1=full([A1,A2,Z_1,Z_2,Z_1,Z_2]);
B=zeros(p,c);
B_1=sparse(1:p,1:p,ones(1,p),p,p);
B_2=sparse(1:p,1:p,-ones(1,p),p,p);
Aeq2=[B,B,full(B_1),full(B_2),full(B_2),full(B_1)];
Aeq=[Aeq1;Aeq2];
Aeq=sparse(Aeq);
clear A1 A2 Z_1 Z_2 Z Y_1;
lb=zeros(2*(c+h+h),1);
[x,f1]=linprog(f,[],[],Aeq,beq,lb);
```

```
x=x';
s=sparse(n,p);
t=sparse(n,p);
s1=sparse(n,p);
t1=sparse(n,p);
J=sparse(n,p);
J1=sparse(n,p);
    for k=1:p
        s(1,k)=x(2*m+k);
        t(1,k)=x(2*m+p+k);
        s1(1,k)=x(2*m+2*p+k);
        t1(1,k)=x(2*m+3*p+k);
    end
J=s-t;
J=full(J);
end
```

Function ODE.m

```
% Function ode_net.m: Inferring dynamic strength matrices of control(asymptomatic) and
%case(symptomatic) samples
function [As_ode_net,S_ode_net]=ode_net(lamda1,lamda2,nt,na,ns)
 %lamda1 is the regularization parameter for guarantee the sparseness
 %lamda2 is the regularization parameter for guarantee the continuity
 %nt is the number of time points
 %na is the number of control(asymptomatic) samples
 %ns is the number of case(symptomatic) samples
 %As_ode_net is a cell array of strength matrices of control(asymptomatic)
 %S_ode_net is a cell array of strength matrices of case(symptomatic)
 load As_cen_diff.mat;%gene second-order central difference expression of control(asymptomatic)
 load S_cen_diff.mat;%gene second-order central difference expression of case(symptomatic)
 load As_adj.mat;%the simplified control(asymptomatic) network based on MI
 load S_adj.mat;%the simplified case(symptomatic) network based on MI
 load data_GC.mat;%sequence of gene expression data
 [m,~]=size(data_GC);
 % construction asymptomatic(control) dynamic network
 As_ode_net{1}=As_adj{1};
 for t=1:nt-1
     As_ode_net{t+1}=zeros(m,m);
     A=As_adj{t};
     X=[];
     for i=1:m
         for s=1:na
            Y(i,s)=As_cen_diff{s}(i,t);
         end
         k=0;
         for j=1:m
             if A(i,j)==1
```

```
                k=k+1;
                X(k,:)=data_CG(j,1+t:nt:na*nt);
                beta(k,1)=As_ode_net{t}(i,j);
            end
        end
        if k~=0
            [J,f1]=LP_OP(Y(i,:),X,lamda1,lamda2,beta);
            k=0;
            for j=1:m
                if A(i,j)==1
                    k=k+1;
                    As_ode_net{t+1}(i,j)=J(k);
                end
            end
            X=[];
            beta=[];
        end
    end
end
save As_ode_net.mat As_ode_net;
%% construction symptomatic(case) dynamic network
S_ode_net{1}=S_adj{1};
for t=1:nt-1
    S_ode_net{t+1}=zeros(m,m);
    A=S_adj{t};
    X=[];
    for i=1:m
        for s=1:ns
            Y(i,s)=S_cen_diff{s}(i,t);
        end
        k=0;
        for j=1:m
            if A(i,j)==1
                k=k+1;
                X(k,:)=data_CG(j,na*nt+1+t: nt:(ns+na)*nt);
                beta(k,1)=S_ode_net{t}(i,j);
            end
        end
        if k~=0
            [J,f1]=LP_OP(Y(i,:),X,lamda1,lamda2,beta);
            k=0;
            for j=1:m
                if A(i,j)==1
                    k=k+1;
                    S_ode_net{t+1}(i,j)=J(k);
                end
            end
```

```
            X=[];
            beta=[];
        end
    end
end
save S_ode_net.mat S_ode_net;
```
